# Supplementary material for: Glycosyl Formates: Glycosylations with Neighboring-Group Participation
Source: Molecules. 2022 Sep 22;27(19):6244. doi: 10.3390/molecules27196244 (PMC9572138; doi:10.3390/molecules27196244)

Supporting Information

Glycosyl Formates: Glycosylations with Neighboring Group Participation

Contents

General information 4

Synthetic procedures and characterization of compounds 5

Synthesis of glucose derivatives 5

Synthesis of Formyl-2-O-benzoyl-3,4,6-tri-O-benzyl-β-D-glucopyranoside(**D1**) 6

Synthesis of 1-Cyclohexyl-2-O-(benzoyl-3,4,6-tri-O-Benzyl-D-glucopyranoside (G1) 7

Synthesis of 1-Adamantyl-2-O-(benzoyl-3,4,6-tri-O-Benzyl-D-glucopyranoside (G2) 8

Synthesis of Methyl 2,3,4-tri-O-benzyl-6-O-(2-benzoyl-3,4,6-tri-O-benzyl-β-D-glucopyranosyl)-α-D-glucopyranoside(G3) 8

Synthesis of Methyl 2,3,6-tri-O-benzyl-4-O-(2-benzoyl-3,4,6-tri-O-benzyl-β-D-glucopyranosyl)-α-D-glucopyranoside (G4) 9

General procedure for the derivatives of galactose 10

The synthesis of Penta-O-benzoyl-D-galactopyranose(S5) 10

Synthesis of 2,3,4,6-Tetra-O-benzoyl-α-D-galactopyranosyl bromide(S6) 11

The synthesis of 1,2-O-(Methoxyphenylmethylene)-3,4,6-tris-O-Benzoyl-α-D-galactopyranose 11

The synthesis of 1,2-O-(Methoxyphenylmethylene)-α-D-galactopyranose 12

The synthesis of 1,2-O-(Methoxyphenylmethylene)-3,4,6-tris-O-Benzyl-α-D-galactopyranose(S9).. 12

Synthesis of Formyl-2-O-benzoyl-3,4,6-tri-O-benzyl-β-D-galactopyranoside(D2) 13

Synthesis of 1-Cyclohexyl-2-O-(benzoyl-3,4,6-tri-O-Benzyl-D-galactopyranoside(G5) 14

Synthesis of 1-Adamantyl-2-O-(benzoyl-3,4,6-tri-O-Benzyl-D-glucopyranoside(G6) 14

Synthesis of Methyl 2,3,4-tri-O-benzyl-6-O-(2-benzoyl-3,4,6-tri-O-benzyl-β-D-galactopyranosyl)-α-D-glucopyranoside(G7) 15

Synthesis of Methyl 2,3,6-tri-O-benzyl-4-O-(2-benzoyl-3,4,6-tri-O-benzyl-β-D-galactopyranosyl)-α-D-glucopyranoside(G8) 16

General procedure for the derivatives of mannose 17

Synthesis of Penta-O-benzoyl-D-mannopyranose(S10) 17

Synthesis of 2,3,4,6-Tetra-O-benzoyl-α-D-mannopyranosyl bromide(S11) 17

Synthesis of 1,2-O-(α-methoxybenzylidene)-3,4,6-tri-O-benzoyl-β-D-mannopyranose (S12) 18

The synthesis of 1,2-O-(Methoxyphenylmethylene)-3,4,6-tri-O-benzyl-α-D-mannopyranose 18

Synthesis of Formyl-2-O-benzoyl-3,4,6-tri-O-benzyl-α-D-mannopyranoside 19

The synthesis of 1-Cyclohexyl-2-O-(benzoyl-3,4,6-tri-O-benzyl-D-mannopyranoside(........ 20

The synthesis of adamantyl 2-O-benzoyl-(3,4,6-tri-O-benzyl)-D-mannopyranoside 21

The synthesis of Methyl 2,3,4-tri-O-benzyl-6-O-(2-benzoyl-3,4,6-tri-O-benzyl-β-D-mannopyranosyl)-α-D-glucopyranoside(G11) 21

The synthesis of Methyl 2,3,6-tri-O-benzyl-4-O-(2-benzoyl-3,4,6-tri-O-benzyl-β-D-mannopyr anosyl)-α-D-glucopyranoside(G12) 22

General procedure for the derivatives of 2-amino-2-deoxy-D-glucose 23

The synthesis of 2-N-alloc-2-deoxy-D-glucopyranose (S14) 23

The synthesis of 2-N-Alloc-2-deoxy-1,3,4,6-tetraacetyl-D-glucopyranose(S15) 23

The synthesis of 2-deoxy-2-[[(2-propenyloxy)carbonyl]amino]-, 3,4,6-triacetate(S16) 24

Synthesis of formyl 2-deoxy-2-N-Alloc-3,4,6-tri-O-acetyl D-glucopyranose (**D4**) 25

Reference 26

Copies of NMR spectra 28

General information

Chemicals were aquired from commericial sources and used without further purification. The solvents (THF, DMF, MECN, MePh, DCM) were of HPLC quality and provided by a solvent system (Innovative Technologies PS-MD-05 solvent drying system). TLC used reation monitoring was aluminum silica gel plates and the developing agent used was 10% H_2SO_4 in ethanol. Purification was performed using flasch column chromatography. Specific rotations were measured on an Anton Paar polarimeter.

NMR spectroscopy was performed at 500 MHz. Chemical shifts were reported relative to TMS (δ 0.00) or solvent residual signals. High-resolution Mass spectrometry was performed on a Bruker SolarX XR 7T E81/MALDI-FTICR-MS.

Synthetic procedures and characterization of compounds

Synthesis of glucose derivatives

The synthesis of 1,2-O-(Methoxyphenylmethylene)-3,4,6-tris-O-(benzyl)-α-D-glucopyranose (1)

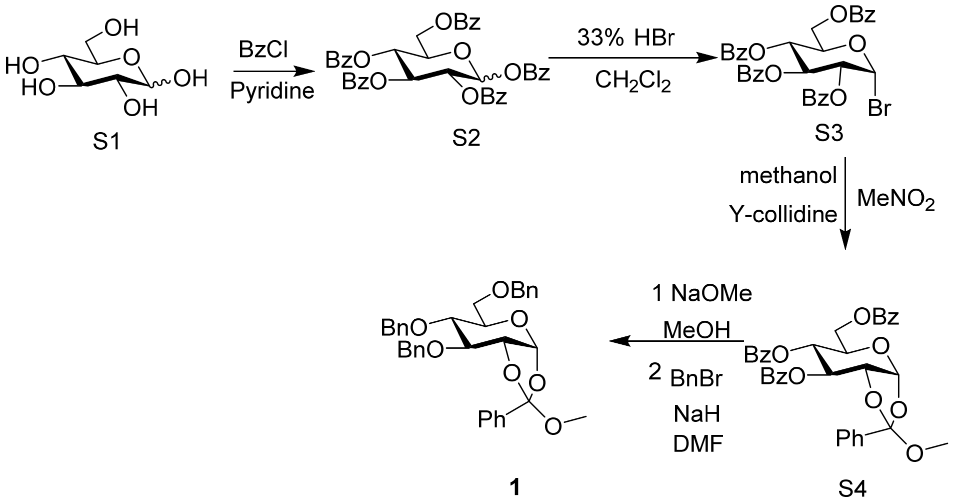

Glucose **S1** (10.0 g, 55.5 mmol) was dissolved in pyridine (50 mL). Then the reaction was stirred for 60 minutes. After that, benzoyl chloride (54g, 388.6 mmol) was added to the reaction in DCM (50 mL) at 0ºC and it was stirred overnight, where TLC showed full conversion. Pyridine was removed in vacuo and the crude was washed with water (100 mL) and extracted with DCM (3 X 100 mL). The combined organic phases were washed with $NaHCO_3$ (100 mL), 1M aq. HCl (100 mL) and brine (100 mL). The organic phase was then dried over $MgSO_4$ and concentrated in vacuo using toluene for azeotropic removal of pyridine, yielding glucose pentabenzoate **S2**.

To a stirred solution of glucose pentabenzoate **S2** (3.00 g, 4.28 mmol) in DCM (30 mL) 33% HBr in acetic acid (3 mL) was added dropwise, and then the reaction was stirred under nitrogen for three days at rt. The reaction mixture was diluted with DCM and washed with water (50 mL), sat. aq. $NaHCO_3$ (3 X 50 mL) and water. The organic phase was concentrated in vacuo to a crude glucosyl bromide **S3** (2.48 g) as a yellow oil.

The glucosyl bromide **S3** (1.50 g, 2.27 mmol) was dissolved in dry nitromethane (25 mL), and then the flask was covered with foil. γ-Collidine (0.28 g, 2.27 mmol) was added to this solution. After that, dry methanol (0.4 mL) was added into the reaction mixture. After stirring for 16 h, the reaction was quenched with Et_3N, filtered and washed with sat. aq. $NaHCO_3$ (50 mL). The aqueous layers were combined and

extracted with DCM (2 X 50 mL). The combined organic phases were washed with water (50 mL) and concentrated in vacuo yielding the crude benzoylated orthoester **S4**.[1]

The benzoylated orthoester **S4** was debenzoylated and benzylated according to literature.[2] **S4** was suspended in methanol (20 mL) followed by addition of NaOMe in methanol (1 mL). The reaction mixture was left stirring at rt for 8 h and was then dried and concentrated in vacuo.

Cooled on an ice bath, the residue was dissolved in DMF (20 mL), and NaH (0.44 g, 18.1 mmol) was added in portions. To the solution was then dropwise added benzyl bromide (2.71 g, 15.8 mmol) over 30 min. while stirring and the reaction was left at rt for 16 h. The reaction was quenched with methanol, extracted by EA (50 mL) and washed with water (100 mL), then concentrated in vacuo. The residue was purified by flash column chromatography on silica gel (1:10 to 1:5 EA/Heptane) yielding **1** (2.42g, 67%, Rf=0.4 in Heptane/EtOAc=5/1-3/1) as a colorless oil; HRMS(ESI): $[M+Na]^+$ calcd for $C_{35}H_{36}O_7Na^+$591.2353, found 591.2357.

^{1}H NMR (500 MHz, $CDCl_3$) δ 7.74 – 7.10 (m, 20H, arom-H), 6.00 (d, *J* = 5.2 Hz, 1H, H1), 4.76 (d, *J* = 11.9 Hz, 1H, $PhCH_2O$-), 4.65 – 4.62 (m, 2H, $PhCH_2O$-, H2), 4.58 (dd, *J* = 11.8, 7.7 Hz, 2H, $PhCH_2O$-), 4.47 (d, *J* = 12.2 Hz, 1H, $PhCH_2O$), 4.39 (d, *J* = 11.3 Hz, 1H, $PhCH_2O$), 3.95 (t, *J* = 3.8 Hz, 1H, H3), 3.81 – 3.72 (m, 2H, H4, H5), 3.64 (d, *J* = 3.0 Hz, 2H, H6A, H6B), 3.21 (s, 3H, $-OCH_3$).

^{13}C NMR (126 MHz, $CDCl_3$) δ 138.12(Arom-C), 138.00, 137.75, 135.96, 129.28, 128.50, 128.33, 128.32, 128.28, 128.04, 127.95, 127.84, 127.73, 127.58, 126.39, 120.62(Arom-C), 98.22(C1), 78.11(C3), 75.62(C2), 75.14(C4), 73.19($-PhCH_2O$-), 72.78($-PhCH_2O$-), 72.11($-PhCH_2O$-), 70.35(C5), 69.04(C6), 51.21(-OCH3).

Synthesis of Formyl-2-O-benzoyl-3,4,6-tri-O-benzyl-β-D-glucopyranoside(D1)[3]

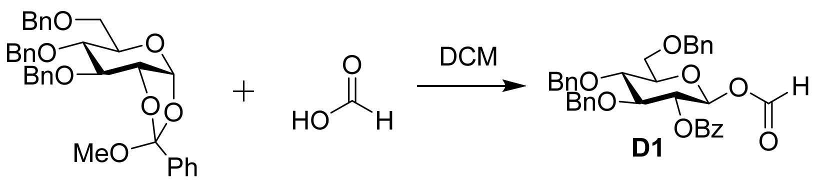

Under a N_2 atm. the orthoester **1** (140 mg, 0.25 mmol) was stirred in DCM (10 mL) and formic acid (0.03 mL) was added at rt. After 2 h, the reaction mixture was concentrated in vacuo. The residue was purified by flash column chromatography on silica gel (Ethyl acetate / Heptane 1/10 - 1/5) affording a colorless oil (127 mg, 89%, Rf:0.34 in Heptane/EtOAc=5/1); $[\alpha]_D^{25}$: 65(c =1, $CHCl_3$).

^{1}H NMR (500 MHz, Chloroform-*d*) δ 8.07 – 8.04 (m, 3H，-CHO，Arom-H), 7.65 – 7.62 (m, 1H, Arom-H), 7.49 (t, *J* = 7.8 Hz, 2H, Arom-H), 7.44 – 7.31 (m, 7H, Arom-H), 7.26 – 7.19 (m, 8H, Arom-H), 5.97 (d, *J* = 8.0 Hz, 1H, H1), 5.52 (t, *J* = 8.4 Hz, 1H, H2, -PhCH$_2$O), 4.89 (d, *J* = 10.8 Hz, 1H, -PhCH$_2$O), 4.83 (d, *J* = 11.2 Hz, 1H, -PhCH$_2$O), 4.75 – 4.70 (m, 2H, -PhCH$_2$O), 4.65 (d, *J* = 10.9 Hz, 1H, -PhCH$_2$O), 4.59 (d, *J* = 12.0 Hz, 1H, -PhCH$_2$O), 4.02 – 3.92 (m, 2H, H3, H4), 3.89 – 3.82 (m, 2H, H6A, H6B), 3.80 – 3.76 (m, 1H, H5).

^{13}C NMR (126 MHz, CDCl$_3$) δ 165.09(-C=O), 159.16(-CHO), 137.89, 137.84, 137.59, 133.48, 129.91, 129.49, 129.36, 128.55, 128.52, 128.48, 128.36, 128.07, 128.03, 127.97, 127.95, 127.81(arom-C), 91.82(β-C1), 82.47(C3), 77.28(C4), 76.18(C5), 75.19(-PhCH$_2$O), 75.15(-PhCH$_2$O), 73.64(-PhCH$_2$O), 72.44(C2), 68.08(C6).

Synthesis of 1-Cyclohexyl-2-O-(benzoyl-3,4,6-tri-O-Benzyl-D-glucopyranoside (G1)

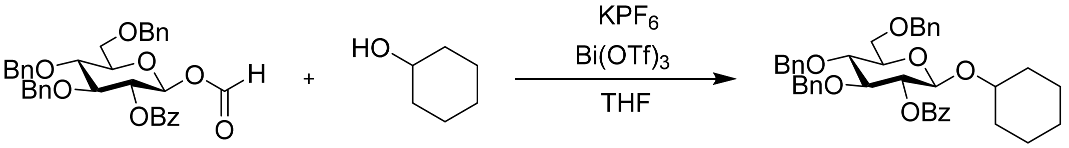

The starting material formyl-2-O-benzoyl-3,4,6-tri-O-benzyl-β-D-glucopyranoside **D1**(121 mg, 0.20 mmol) was dissolved in THF (3.0 mL). Then, Cyclohexanol (23 mg, 0.23 mmol), KPF$_6$ (41 mg, 0.23 mmol) and Bi(OTf)$_3$ (39 mg, 0.05 mmol) were added. After 4 hours at rt under N$_2$ protection, the reaction was quenched with TEA, aqueous worked-up using EA and finally concentrated in vacuo. Purification by flash column chromatography on silica gel (Ethyl acetate / Heptane 1:20 – 1:10) gave a white solid (84 mg, 65%, β, Rf:0.4 in Heptane/EtOAc=5/1); $[\alpha]_D^{25}$: 25(c=1, CHCl$_3$). HRMS(ESI): $[M+Na]^+$calcd for $C_{35}H_{34}O_8Na^+$659.2979, found 659.2994

^{1}H NMR (500 MHz, CDCl$_3$) δ 7.94 – 7.89 (m, 3H, Arom-H), 7.53 – 7.47 (m, 1H, Arom-H), 7.36 (t, *J* = 7.8 Hz, 3H, Arom-H), 7.28 – 7.01 (m, 13H, Arom-H), 5.82 (d, *J* = 8.1 Hz, 1H, , H1), 5.37 (t, *J* = 8.5 Hz, 1H, H2), 4.75 (d, *J* = 1.8 Hz, 1H, -PhCH2O), 4.68 (d, *J* = 11.2 Hz, 1H, -PhCH2O), 4.61 – 4.56 (m, 2H -PhCH2O), 4.50 (d, *J* = 10.7 Hz, 1H -PhCH2O), 4.45 (d, *J* = 9.6 Hz, 1H -PhCH2O), 3.82 (m, *J* = 12.5, 6.4 Hz, 2H, H3, H4), 3.70 (q, *J* = 4.6, 4.0 Hz, 2H, H6A, H6B), 3.65 – 3.61 (m, 1H, H5), 3.53 (m, *J* = 9.2, 4.7 Hz, 1H, -OCH-), 1.85 – 1.79 (m, 2H, -CH2-C), 1.69 – 1.64 (m, 2H -CH2-C), 1.48 (ddd, *J* = 8.9, 5.7, 1.8 Hz, 2H -CH2-C), 1.19 (t, *J* = 4.7 Hz, 4H, -CH2-C).

^{13}C NMR (126 MHz, CDCl$_3$) δ 159.11(-PhC=O), 137.82(Arom-C), 137.52, 133.42, 129.86, 129.29, 128.49, 128.46, 128.43, 128.31, 128.30, 128.01, 127.99, 127.96, 127.93, 127.90, 127.76(Arom-C), 91.76(C1), 82.42(C3), 76.73(C4), 76.13(C5), 75.15(-PhCH2O-), 75.12(-PhCH2O-), 73.60(-PhCH2O-), 72.38(C2), 70.36(-OCH-), 68.02(C6), 35.57(-CH2C-), 25.47(-CH2C-), 24.13(-CH2C-).

Synthesis of 1-Adamantyl-2-O-(benzoyl-3,4,6-tri-O-Benzyl-D-glucopyranoside (G2)[4]

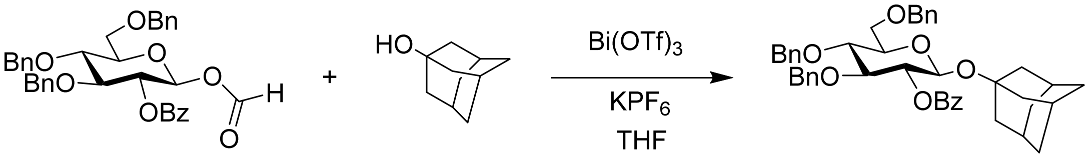

Formyl-2-O-benzoyl-3,4,6-tri-O-benzyl-β-D-glucopyranoside **D1** (140 mg, 0.24 mmol) was dissolved in THF (2.0 mL). While stirring, 1-adamantanol (44 mg, 0.29 mmol), KPF_6 (53 mg, 0.29 mmol) and $Bi(OTf)_3$ (39 mg, 0.06 mmol) were added. After 4 hours at rt under a nitrogen atmosphere, the reaction was quenched with TEA, aqueous worked-up using EA and finally concentrated in vacuo. Purification by flash column chromatography on silica gel (Ethyl acetate / Heptane 1:20 – 1:10) gave a white solid (111 mg, 67%, α/β=1/6, Rf:0.46 in Heptane/EtOAc=5/1); $[\alpha]^{25}_{D}$: 35 (c=1, CHCl3).

^{1}H NMR (500 MHz, $CDCl_3$) δ 8.05 – 8.02 (m, 2H， arom-H), 7.61 – 7.57 (m, 1H, arom-H), 7.46 (d, J = 7.59 Hz, 2H, arom-H), 7.39 – 7.29 (m, 9H, arom-H), 7.26 – 7.23 (m, 2H, arom-H), 7.14 (t, J = 3.2 Hz, 4H, arom-H), 5.26 (dd, J = 9.5, 8.0 Hz, 1H, H2), 4.86 (d, J = 11.0 Hz, 1H, $PhCH_2O$), 4.83 (d, J = 8.0 Hz, 1H, H1), 4.75 (d, J = 11.2 Hz, 1H, $PhCH_2O$), 4.68 – 4.59 (m, 4H, $PhCH_2O$), 3.85 (t, J = 9.2 Hz, 1H, H3), 3.80 (dd, J = 10.8, 2.0 Hz, 1H, H6), 3.73 – 3.70 (m, 1H, H6), 3.70 – 3.68 (m, 1H, H4), 3.58 (ddd, J = 9.8, 5.6, 2.0 Hz, 1H, H5), 2.08 (p, J = 3.2 Hz, 3H, adamantyl), 1.82 (dq, J = 11.3, 2.6 Hz, 3H, adamantyl), 1.69 – 1.64 (m, 3H, adamantyl), 1.61 – 1.51 (m, 6H, adamantyl).

^{13}C NMR (126 MHz, $CDCl_3$) δ 165.04, 138.38, 138.05, 137.90, 132.87, 130.32, 129.71, 128.42, 128.34, 128.32, 128.22, 128.08, 128.01, 127.81, 127.63, 127.58, 127.51(armo-C）, 94.03(β-C1), 83.13(C3), 78.38(C4), 75.07(C5), 75.00(-$PhCH_2O$), 74.96(-$PhCH_2O$), 74.94 (C2), 74.08(-$PhCH_2O$), 73.44, 69.33(C6), 42.39(adamantyl), 36.14(adamantyl), 30.57 (adamantyl).

Synthesis of Methyl 2,3,4-tri-O-benzyl-6-O-(2-benzoyl-3,4,6-tri-O-benzyl-β-D-glucopyranosyl)-α-D-glucopyranoside(G3)[5]

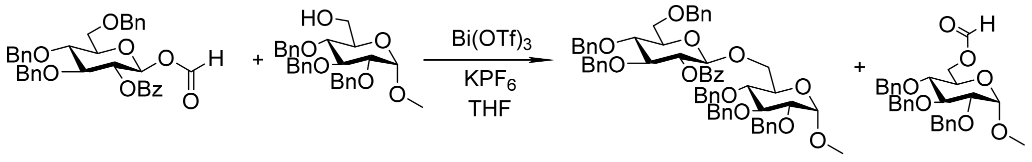

Formyl-2-O-benzoyl-3,4,6-tri-O-benzyl-β-D-glucopyranoside **D1** (80 mg, 0.14 mmol) was dissolved in THF (2.0 mL). While stirring, methyl 2,3,4-tri-O-benzyl-α-D-glucopyranoside (77 mg, 0.16 mmol), KPF_6 (30 mg, 0.16 mmol) and $Bi(OTf)_3$ (23 mg, 0.04 mmol) were added. After 4 hours at rt under a nitrogen atmosphere, the reaction was quenched with TEA, aqueous worked-up using EA and finally concentrated

in vacuo. Purification by flash column chromatography on silica gel (Ethyl acetate / Heptane 1:10 – 1:5) afforded a white solid (98 mg, 72%, β, Rf:0.34 in Heptane/EtOAc=5/1), [α] 25 D: 30.7 (c 0.4, $CHCl_3$), and a side product (8 mg).

^{1}H NMR (500 MHz, $CDCl_3$) δ 7.98 – 7.93 (m, 2H, arom-H), 7.64 – 7.57 (m, 1H, arom-H), 7.49 – 7.45 (m, 2H, arom-H), 7.36 – 7.30 (m, 16H, arom-H), 7.27 – 7.24 (m, 5H, arom-H), 7.23 – 7.20 (m, 3H, arom-H), 7.14 (s, 4H, arom-H), 7.06 – 7.01 (m, 2H, arom-H), 5.37 (dd, *J* = 9.4, 8.0 Hz, 1H, H2), 4.89 (d, *J* = 11.0 Hz, 1H, -$PhCH_2O$), 4.82 (s, 1H, -$PhCH_2O$), 4.75 (dd, *J* = 11.6, 5.2 Hz, 2H, -$PhCH_2O$), 4.69 (dd, *J* = 11.1, 6.2 Hz, 2H, -$PhCH_2O$), 4.65 – 4.57 (m, 4H, , -$PhCH_2O$), 4.55 (d, *J* = 7.9 Hz, 1H, H1), 4.49 (d, *J* = 3.5 Hz, 1H, H1'), 4.46 (d, *J* = 11.0 Hz, 1H, , -$PhCH_2O$), 4.30 (d, *J* = 11.0 Hz, 1H, -$PhCH_2O$), 4.16 (dd, *J* = 10.4, 1.7 Hz, 1H, -$PhCH_2O$), 3.89 (t, *J* = 9.3 Hz, 1H, H3'), 3.84 (t, *J* = 9.2 Hz, 1H, H3), 3.81 – 3.65 (m, 5H, H4, H6A,H6B, H5', H6A', H6B'), 3.58 (ddd, *J* = 9.7, 5.2, 2.0 Hz, 1H, H5), 3.45 (dd, *J* = 9.7, 3.5 Hz, 1H, H2'), 3.39 (t, *J* = 9.4 Hz, 1H, H4'), 3.21 (s, 3H, -OME).

^{13}C NMR (126 MHz, $CDCl_3$) δ 165.00(C=O), 138.91, 138.23, 137.90, 137.77, 132.95, 129.86, 129.82, 129.75, 128.45, 128.42, 128.37, 128.34, 128.27, 128.24, 128.13, 128.03, 127.87, 127.85, 127.83, 127.70, 127.66, 127.60, 127.48, 127.41(armo-C), 101.25(β-C1), 97.93(α–C1'), 82.85(β-C3), 81.92(α–C3'), 79.74(α–C2'), 78.08(β-C4), 77.47(α–C4'), 75.56 (α–C5'), 75.13(-$PhCH_2O$), 75.11(-$PhCH_2O$), 74.64(-$PhCH_2O$), 73.68(-$PhCH_2O$), 73.56 (β-C2), 73.48(-$PhCH_2O$), 73.37(-$PhCH_2O$), 70.55(β-C2), 69.48(β-C5), 68.93(α–C6'), 68.05(β-C6), 54.93(-OMe).

Synthesis of Methyl 2,3,6-tri-O-benzyl-4-O-(2-benzoyl-3,4,6-tri-O-benzyl-β-D-glucopyranosyl)-α-D-glucopyranoside (G4)[6]

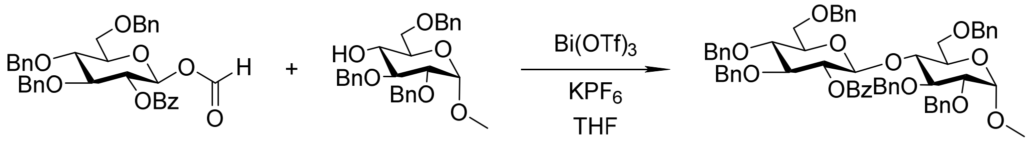

Formyl-2-O-benzoyl-3,4,6-tri-O-benzyl-β- D-glucopyranoside **D1** (120 mg, 0.21 mmol) was dissolved in THF (2.0 mL). While stirring, methyl 2,3,6-tri-O-benzyl-α-D-glucopyranoside (115 mg, 0.25 mmol), KPF_6 (46 mg, 0.25 mmol) and $Bi(OTf)_3$ (34 mg, 0.05 mmol) were added. After 3 hours at rt under a nitrogen atmosphere, the reaction was quenched with TEA, aqueous worked-up using EA and finally concentrated in vacuo. Purification by flash column chromatography on silica gel (Ethyl acetate / Heptane 1:20 – 1:10) gave a white solid (167 mg, 81%, β, Rf:0.4 in Heptane/EtOAc=5/1); [α] 25 D: 33.6(c 0.77, $CHCl_3$).

^{1}H NMR (500 MHz, $CDCl_3$) δ 8.10 – 8.05 (m, 2H, arom-H), 7.62 – 7.57 (m, 1H, arom-H), 7.47 (d, *J* = 7.8 Hz, 2H, arom-H), 7.41 – 7.28 (m, 31H, arom-H), 7.23 – 7.20 (m, 5H, arom-H), 5.58 (t, *J* = 3.5 Hz, 1H, H1), 5.16 (dd, *J* = 10.0, 3.7 Hz, 1H, H2), 5.03 (d, *J* = 11.5 Hz, 1H, -$PhCH_2O$), 4.89 – 4.83 (m, 3H, -$PhCH_2O$), 4.80 (d, *J* = 12.0 Hz, 1H, -$PhCH_2O$), 4.78 (s, 1H, -$PhCH_2O$), 4.69 (d, *J* = 12.1 Hz, 1H, -$PhCH_2O$), 4.66 (d,

$J = 3.6$ Hz, 1H, H1'), 4.62 (d, $J = 12.1$ Hz, 2H, -PhCH_2O), 4.57 (dd, $J = 11.4$, 2.2 Hz, 3H, -PhCH_2O), 4.26 (t, $J = 9.5$ Hz, 1H, H3), 4.17 (ddd, $J = 10.1$, 4.5, 2.3 Hz, 1H, H5), 3.81 (t, $J = 9.2$ Hz, 1H, H3'), 3.76 – 3.70 (m, 6H, H4, H6A, H6B, H5', H6A', H6B'), 3.63 (td, $J = 9.2$, 1.9 Hz, 1H, H4'), 3.56 (dd, $J = 9.5$, 3.5 Hz, 1H, H2'), 3.41 (s, 3H, -OMe).

^{13}C NMR (126 MHz, $CDCl_3$) δ 165.86, 138.82, 138.16, 138.07, 138.02, 137.89, 133.26, 129.83, 128.61, 128.49, 128.44, 128.42, 128.37, 128.34, 128.14, 128.02, 127.96, 127.92, 127.86, 127.79, 127.76, 127.66, 127.64(arom-C), 98.21(α-C1'), 90.66(β-C1), 81.48(α-C3'), 79.68(β-C3), 79.61(α-C2'), 78.11(β-C4), 75.56(-PhCH_2O), 75.44(-PhCH_2O), 75.12 (-PhCH_2O), 74.19(β-C2), 73.60(-PhCH_2O), 73.54(-PhCH_2O), 73.18(-PhCH_2O), 70.75(α-C4'), 70.48(α-C5'), 69.89(β-C5), 69.50(α-C6'), 68.75(β-C6), 55.26(-OMe). Signals missing possibly due to overlap. peaks are in agreement with previously reported spectra.

General procedure for the derivatives of galactose

The synthesis of Penta-O-benzoyl-D-galactopyranose(S5)[7,8]

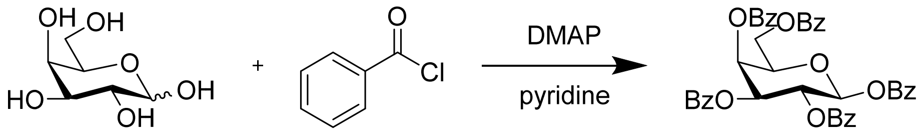

D-galactose (10 g, 55.51 mmol) was added to a solution of pyridine (40 mL) in dichloromethane (50 mL) at 0°C and stirred for 30 min. Benzoyl chloride (54.62 g, 389 mmol) and DMAP (0.67 g, 5.55 mmol) were added to the mixture and stirred at room temp. overnight. The reaction mixture was diluted with CH_2Cl_2 (100 mL) and then washed with water (2 × 100 mL) and brine (100 mL). The organic layer was dried over Na_2SO_4 and after removal of the solvent under vacuum, the residue was purified by column chromatography on silica gel using chloroform to afford a white solid (37.6 g, 97 %).

^{1}H NMR (500 MHz, $CDCl_3$) δ 8.15 (ddd, $J = 8.1$, 3.7, 2.4 Hz, 5H, Arom-H), 8.03 – 7.96 (m, 2H, Arom-H), 7.91 – 7.84 (m, 4H, Arom-H), 7.67 (td, $J = 7.4$, 1.4 Hz, 2H, Arom-H), 7.56 – 7.28 (m, 12H, Arom-H), 6.99 (d, $J = 3.7$ Hz, 1H, H1), 6.23 (dd, $J = 3.4$, 1.4 Hz, 1H, H4), 6.17 (dd, $J = 10.7$, 3.3 Hz, 1H, H3), 6.07 (dd, $J = 10.7$, 3.6 Hz, 1H, H2), 4.88 (td, $J = 6.6$, 1.4 Hz, 1H, H5), 4.67 (dd, $J = 11.4$, 6.4 Hz, 1H, H6A), 4.46 (dd, $J = 11.4$, 7.0 Hz, 1H, H6B).

^{13}C NMR (126 MHz, $CDCl_3$) δ 165.94, 165.73, 165.58, 165.51, 164.56(C=O), 133.92, 133.75, 133.57, 133.49, 133.42, 133.25, 130.16, 129.99, 129.96, 129.78, 129.77, 129.63, 129.30, 129.02, 128.98, 128.91,

128.85, 128.80, 128.75, 128.72, 128.46, 128.44, 128.42, 128.37(Arom-C), 90.70(C1), 69.47(C5), 68.56(C3), 68.50(C4), 67.72(C2), 61.86(C6).

Synthesis of 2,3,4,6-Tetra-O-benzoyl-α-D-galactopyranosyl bromide(S6)[7,8]

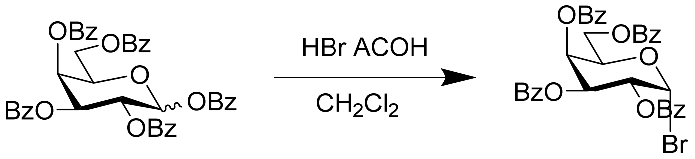

HBr (1.6 mL, 6.42 mmol, 33% in AcOH) was added dropwise to compound **S5** (980 mg, 1.28 mmol) dissolved in DCM at 0 °C, then stirred for 0.5 h and then at room temperature for 3 h. The reaction mixture was diluted with dichloromethane (20 mL), washed with cold water (20 mL) and then cold saturated $NaHCO_3$ solution (10 mL). The organic layer was dried over Na_2SO_4 and the solvent was removed by rotary evaporation to obtain a brownish white solid (860 mg, 96 %),

^{1}H NMR (500 MHz, $CDCl_3$) δ 8.12 – 7.78 (m, 6H, arom-H), 7.68 – 7.24 (m, 14H, arom-H), 7.01 (d, *J* = 4.0 Hz, 1H, H1), 6.15 (dd, *J* = 3.5, 1.3 Hz, 1H, H4), 6.09 (dd, *J* = 10.5, 3.4 Hz, 1H, H3), 5.70 (dd, *J* = 10.4, 4.0 Hz, 1H, H2), 4.95 (t, *J* = 6.5 Hz, 1H, H5), 4.67 (dd, *J* = 11.6, 6.8 Hz, 1H, H6A), 4.50 (dd, *J* = 11.5, 6.0 Hz, 1H, H6B).

^{13}C NMR (126 MHz, $CDCl_3$) δ 165.94, 165.57, 165.37, 165.33(C=O), 133.80, 133.40, 133.34, 130.03, 129.96, 129.83, 129.76, 129.26, 128.81, 128.74, 128.58, 128.56, 128.50, 128.47, 128.35(arom-C), 88.29(C1), 71.85(C5), 68.90(C3), 68.62(C2), 68.09(C4), 61.69(C6).

The synthesis of 1,2-O-(Methoxyphenylmethylene)-3,4,6-tris-O-Benzoyl-α-D-galactopyranose(S7)

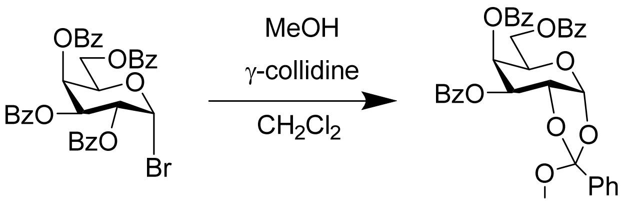

The glycosyl bromide **S6** (7.00 g, 10.6 mmol) was dissolved in dry nitromethane (50 mL), and then the flask was covered with foil. γ-Collidine (2.00 mL, 10.6 mmol) was added to this solution. After that, dry methanol (2 mL) was added to the reaction followed by stirring for 16 h. The reaction was quenched with Et_3N, filtered and washed with sat. aq. $NaHCO_3$ (100 mL). The aqueous layers were combined and extracted with DCM (2 X 100 mL). The combined organic phases were washed with water (100 mL) and concentrated in vacuo yielding the crude benzoylated orthoester **S7**.

^{1}H NMR (500 MHz, $CDCl_3$) δ 8.00 – 7.94 (m, 7H, arom-H), 7.68 (ddd, *J* = 6.6, 3.5, 1.9 Hz, 2H, arom-H), 7.59 – 7.53 (m, 4H, arom-H), 7.45 – 7.37 (m, 7H, arom-H), 6.24 (d, *J* = 5.0 Hz, 1H, -H1), 5.84 (dd, *J* = 4.2, 2.6 Hz, 1H, H4), 5.56 (dd, *J* = 6.1, 4.1 Hz, 1H, H3), 4.81 (dd, *J* = 6.1, 5.0 Hz, 1H, H2), 4.62 (dd, *J* = 11.3, 6.7 Hz, 1H, H6A), 4.55 (td, *J* = 6.7, 6.2, 2.6 Hz, 1H, H5), 4.41 (dd, *J* = 11.3, 5.6 Hz, 1H, H6B), 3.27 (s, 3H, CH3).

^{13}C NMR (126 MHz, $CDCl_3$) δ 166.00, 165.24, 165.21(C=O), 136.27, 133.56, 133.39, 133.17(arom-C), 129.85, 129.83, 129.79, 129.77, 129.61, 129.45, 129.16, 128.97, 128.66, 128.63, 128.55, 128.50, 128.39, 128.36, 126.08, 126.06, 120.66(arom-C), 98.24(C1), 73.58(C2), 70.18(C3), 68.93(C5), 66.56(C4), 62.37(C6), 51.13(CH3).

The synthesis of 1,2-O-(Methoxyphenylmethylene)-α-D-galactopyranose**(S8)**

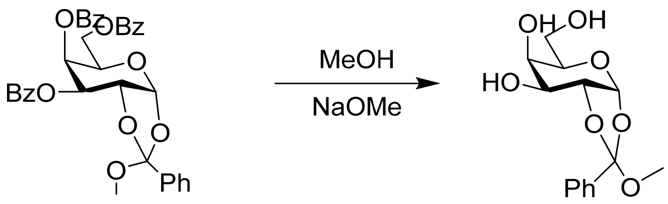

The starting material **S7** (3.00 g, 4.91 mmol) was dissolved in MeOH (30 mL), then, NaOMe (53 mg, 1.0 mmol) was added. The reaction mixture was left stirring at rt for 8 h, then dried over $MgSO_4$ and concentrated in vacuo.

^{1}H NMR (500 MHz, MeOD) δ 7.69 – 7.63 (m, 2H, Arom-H)), 7.37 (qd, *J* = 3.8, 1.6 Hz, 3H, Arom-H), 5.59 (d, *J* = 2.7 Hz, 1H, α-H1), 4.68 (dd, *J* = 4.2, 2.7 Hz, 1H, H2), 3.82 (dd, *J* = 9.5, 4.1 Hz, 1H, H3), 3.79 – 3.74 (m, 1H, H6A), 3.54 (t, *J* = 9.5 Hz, 1H, H4), 3.47 (dd, *J* = 11.9, 6.8 Hz, 1H, H6B), 3.32 – 3.27 (m, 4H, H5, CH_3).

^{13}C NMR (126 MHz, MeOD) δ 139.41, 129.97, 128.87, 127.72(Arom-C), 123.81(-PhC-), 99.26(α-C1), 80.98(C2), 77.52(C5), 72.96(C3), 68.73(C4), 63.09(C6), 51.28(-CH_3).

The synthesis of 1,2-O-(Methoxyphenylmethylene)-3,4,6-tris-O-Benzyl-α-D-galactopyranose(S9)

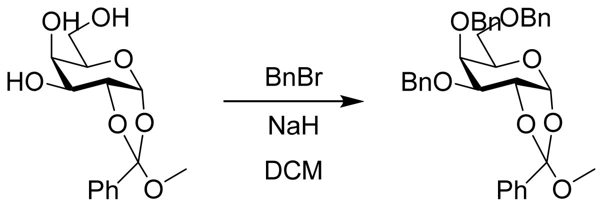

On an ice bath, the residue (1.5 g, 5.03 mmol) was dissolved in DMF (40 mL), and NaH (0.48 g, 20.1 mmol) was added in portions. To this stirring solution was dropwise added benzyl bromide (3.01 g, 17.6 mmol)

over 30 min, and the reaction was left stirring at rt for 16 h. The reaction was quenched with methanol, extracted by EA (50 mL) and the organic phase washed with water (4x100 mL). The combined organic phases were then concentrated in vacuo. The residue was purified by flash column chromatography on silica gel (1:10 to 1:5 EA/Heptane) yielding **S9** (2.41g, 64%, Rf=0.4 in Heptane/EtOAc=5/1) as a colorless oil; $[\alpha]_D^{25}$: -38 (c =1, $CHCl_3$). HRMS(ESI): $[M+Na]^+$ calcd for $C_{35}H_{36}O_7Na^+$ 591.2353, found 591.2362.

^{1}H NMR (500 MHz, $CDCl_3$) δ 7.63 – 7.59 (m, 3H, Arom-H), 7.41 – 7.26 (m, 17H, Arom-H), 5.98 (d, *J* = 4.8 Hz, 1H, H1), 4.92 – 4.87 (m, 1H, -PhCH2O-), 4.70 – 4.65 (m, 2H, H2, -PhCH2O-), 4.59 (d, *J* = 11.6 Hz, 1H, -PhCH2O-), 4.49 (t, *J* = 6.0 Hz, 2H, -PhCH2O-), 4.46 – 4.42 (m, 1H, -PhCH2O-), 3.99 (ddd, *J* = 7.5, 5.7, 1.7 Hz, 1H, H5), 3.86 (dd, *J* = 2.6, 1.8 Hz, 1H, H4), 3.65 – 3.60 (m, 2H, H6A, H6B), 3.47 (dd, *J* = 7.0, 2.6 Hz, 1H, H3), 3.29 (s, 3H, -OCH3).

^{13}C NMR (126 MHz, $CDCl_3$) δ 138.42 (Arom-C), 138.32, 138.07, 137.75, 129.02, 128.43, 128.36, 128.30, 128.14, 128.07, 128.04, 127.94, 127.90, 127.83, 127.79, 127.63, 127.59, 125.88 (Arom-C), 119.85(-CCO3-), 98.71(C1), 79.30(C2), 77.99(C3), 74.45(-PhCH2O-), 73.54(-PhCH2O-), 72.60(-PhCH2O-), 72.46(C4), 71.80(C5), 68.21(C6), 50.58(-OCH3).

Synthesis of Formyl-2-O-benzoyl-3,4,6-tri-O-benzyl-β-D-galactopyranoside(D2)

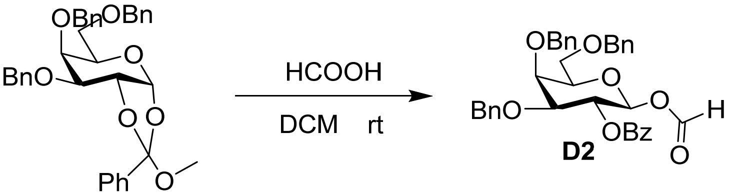

The 1,2-orthoester **S9** (500 mg, 0.88 mmol) was dissolved in DCM (10 mL), then formic acid (56 mg, 1.14 mmol) was added at rt. After 2 h, the reaction mixture was concentrated in vacuo. The residue was purified by flash column chromatography on silica gel (EA / Heptane 1/10 - 1/5) affording a colorless oil (414 mg, 81%, Rf:0.41 in Heptane/EtOAc=5/1); $[\alpha]_D^{25}$: 111(c =1, $CHCl_3$). HRMS(ESI): $[M+Na]^+$ calcd for $C_{35}H_{34}O_8Na^+$605.2146, found 605.2153.

^{1}H NMR (500 MHz, $CDCl_3$) δ 8.03 – 7.99 (m, 3H, CHO, Arom-H), 7.63 – 7.59 (m, 1H, Arom-H), 7.49 – 7.45 (m, 2H, Arom-H), 7.39 – 7.15 (m, 15H), 5.88 – 5.79 (m, 2H, H1, H2), 5.02 (d, *J* = 11.5 Hz, 1H, -PhCH2O), 4.67 (dd, *J* = 11.9, 5.7 Hz, 2H, -PhCH2O), 4.54 – 4.43 (m, 3H, -PhCH2O), 4.09 (dd, *J* = 2.8, 1.2 Hz, 1H, H4), 3.82 (ddd, *J* = 7.2, 5.6, 1.2 Hz, 1H, H5), 3.74 (dd, *J* = 9.5, 2.8 Hz, 1H, H3), 3.71 – 3.62 (m, 2H, -H6A, -H6B).

^{13}C NMR (126 MHz, $CDCl_3$) δ 165.15（C=O), 159.14(CHO), 138.19(Arom-C), 137.63, 137.31, 133.28, 129.91, 129.55, 128.50, 128.42, 128.38, 128.31, 128.27, 128.03, 127.95, 127.83, 127.73, 127.71(Arom-C),

92.18(C1), 79.55(C3), 74.77(C5), 74.69(-PhCH2O), 73.64(-PhCH2O), 72.12(C4), 71.87(-PhCH2O), 70.49(C2), 67.92(C6).

Synthesis of 1-Cyclohexyl-2-O-(benzoyl-3,4,6-tri-O-Benzyl-D-galactopyranoside(G5)

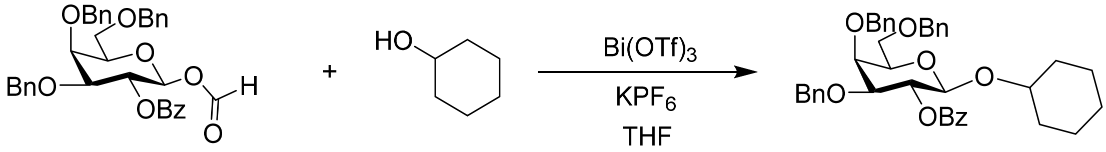

Formyl 2-O-benzoyl-3,4,6-tri-O-benzyl-β-D-glucopyranoside **D2** (120 mg, 0.20 mmol) was dissolved in THF (3.0 mL). Then, Cyclohexanol (23 mg, 0.23 mmol), KPF_6 (41 mg, 0.23 mmol) and $Bi(OTf)_3$ (38 mg, 0.05 mmol) were added. After 4 hours at rt under a N_2 atmosphere, the reaction was quenched with TEA, aqueous worked-up using EA and finally concentrated in vacuo. Purified by flash column chromatography on silica gel (Ethyl acetate / Heptane 1:20 – 1:10) gave a white solid (93 mg, 71%, β, Rf:0.4 in Heptane/EtOAc=5/1); $[\alpha]_D^{25}$: 20(c=1, $CHCl_3$). HRMS(ESI): $[M+Na]^+$calcd for $C_{35}H_{34}O_8Na^+$659.2979, found 659.2991.

^{1}H NMR (500 MHz, $CDCl_3$) δ 8.07 – 8.04 (m, 2H, Arom-H), 7.60 (ddt, J = 7.0, 5.2, 1.4 Hz, 1H), 7.47 (t, J = 7.8 Hz, 2H), 7.40 – 7.27 (m, 10H), 7.23 – 7.14 (m, 5H, Arom-H), 5.63 (dd, J = 10.1, 7.9 Hz, 1H, H2), 5.02 (d, J = 11.7 Hz, 1H, -PhCH2O), 4.68 (dd, J = 12.1, 7.0 Hz, 2H, -PhCH2O), 4.59 (d, J = 7.9 Hz, 1H, H1), 4.53 – 4.48 (m, 3H, -PhCH2O), 4.04 – 4.01 (m, 1H, H4), 3.72 – 3.59 (m, 5H, H3, H5, -O-CH-), 1.89 – 1.81 (m, 1H, -C-CH2-C), 1.70 – 1.64 (m, 2H, -C-CH2-C), 1.53 (dddd, J = 12.8, 9.8, 7.4, 3.8 Hz, 1H, -C-CH2-C), 1.45 – 1.36 (m, 2H, -C-CH2-C), 1.21 (tdd, J = 12.7, 7.8, 2.9 Hz, 2H, -C-CH2-C), 1.14 – 1.08 (m, 2H, -C-CH2-C).

^{13}C NMR (126 MHz, $CDCl_3$) δ 165.29(C=O), 138.52(Arom-C), 137.97, 137.79, 132.78, 130.51, 129.73, 128.47, 128.45, 128.40, 128.29, 128.27, 128.21, 128.07, 127.94, 127.87, 127.83, 127.68, 127.62, 127.59(Arom-C), 100.05(C1), 80.11(C3), 77.28(-O-CH-), 74.41(-PhCH2O), 73.71(C5), 73.61(-PhCH2O), 72.43(C4), 72.25(C2), 71.66(-PhCH2O), 68.91(C6), 33.23(-C-CH2-C), 31.51(-C-CH2-C), 25.50(-C-CH2-C), 23.72(-C-CH2-C), 23.50(-C-CH2-C).

Synthesis of 1-Adamantyl-2-O-(benzoyl-3,4,6-tri-O-Benzyl-D-glucopyranoside(G6)

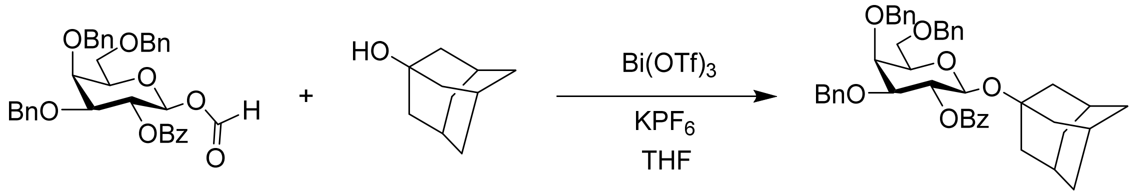

D2 (120 mg, 0.20 mmol) was dissolved in THF (3.0 mL). Then, 1-adamantanol (34 mg, 0.23 mmol), KPF_6 (38 mg, 0.20 mmol) and $Bi(OTf)_3$ (38 mg, 0.05 mmol) were added. After 4 hours at rt under a N_2 atmosphere,

the reaction was quenched with TEA, aqueous worked-up using EA and finally concentrated in vacuo. Purification by flash column chromatography on silica gel (Ethyl acetate / Heptane 1:20 – 1:10) gave colorless oil product (101 mg, 72%, β, Rf:0.4 in Heptane/EtOAc=5/1); $[\alpha]_D^{25}$: 2(c=1, $CHCl_3$). HRMS(ESI): $[M+Na]^+$calcd for $C_{35}H_{34}O_8Na^+$ 711.3292, found 711.3312.

^{1}H NMR (500 MHz, $CDCl_3$) δ 8.15 – 8.07 (m, 2HArom-H), 7.57 (td, *J* = 7.4, 1.5 Hz, 1H, Arom-H), 7.43 – 7.22 (m, 17H, Arom-H), 5.46 (t, *J* = 2.6 Hz, 1H, H2), 5.42 (d, *J* = 2.1 Hz, 1H, H1), 4.91 (d, *J* = 10.6 Hz, 1H, -PhCH2O), 4.80 (dd, *J* = 23.6, 11.6 Hz, 2H, -PhCH2O), 4.62 (d, *J* = 11.3 Hz, 1H, -PhCH2O), 4.56 (dd, *J* = 11.4, 5.1 Hz, 2H, -PhCH2O), 4.23 – 4.19 (m, 1H, H3), 4.16 – 4.09 (m, 2H, H4, H5), 3.95 (dd, *J* = 10.7, 3.3 Hz, 1H, H6A), 3.79 (dd, *J* = 10.6, 1.5 Hz, 1H, H6B), 2.18 – 2.13 (m, 3H, adamantyl), 1.89 – 1.83 (m, 6H, adamantyl), 1.67 – 1.60 (m, 6H, adamantyl).

^{13}C NMR (126 MHz, $CDCl_3$) δ 166.01(C=O), 138.60(Arom-C), 138.51, 138.23, 133.03, 130.13, 129.98, 128.37, 128.35, 128.29, 128.09, 128.06, 127.65, 127.56, 127.49, 127.42(Arom-C), 91.07(C1), 78.35(C3), 75.35(-PhCH2O), 75.14(adamantyl), 74.76(C4), 73.40(-PhCH2O), 71.52(-PhCH2O), 71.19(C5), 70.71(C2), 69.29(C6), 42.33(adamantyl), 36.24(adamantyl), 30.64(adamanty).

Synthesis of Methyl 2,3,4-tri-O-benzyl-6-O-(2-benzoyl-3,4,6-tri-O-benzyl-β-D-galactopyranosyl)-α-D-glucopyranoside(G7)

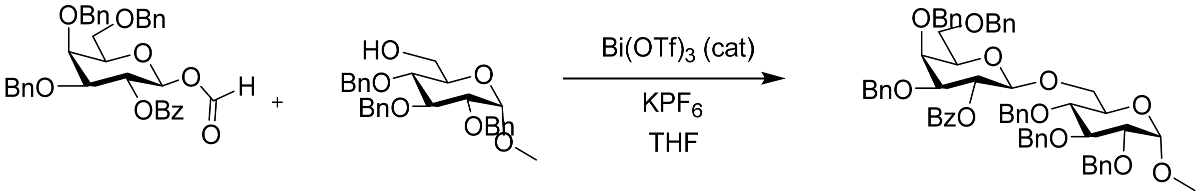

D2 (120 mg, 0.20 mmol) was dissolved in THF (3.0 mL). Then, acceptor 2,3,4-tri-O-benzyl-α-D-glucopyranoside (105 mg, 0.23 mmol), KPF_6 (38 mg, 0.20 mmol) and $Bis(OTf)_3$ (38 mg, 0.05 mmol) were added. After 4 hours at rt under N_2 protection, the reaction was quenched with TEA, aqueous worked-up using EA and finally concentrated in vacuo. Purification by flash column chromatography on silica gel (Ethyl acetate / Heptane 1:20 – 1:10), gave a colorless oil (150 mg, 73%, β, Rf:0.4 in Heptane/EtOAc=5/1); $[\alpha]_D^{25}$: 49(c=1, $CHCl_3$). HRMS(ESI): $[M+Na]^+$calcd for $C_{35}H_{34}O_8Na^+$1023.4290, found 1023.4304.

^{1}H NMR (500 MHz, $CDCl_3$) δ 7.95 (t, *J* = 5.9 Hz, 2H, Arom-H), 7.37 – 7.19 (m, 33H, Aaom-H), 5.70 (ddd, *J* = 11.6, 8.0, 3.6 Hz, 1H, H2'), 4.98 (dd, *J* = 11.5, 3.6 Hz, 1H, -PhCH2O), 4.88 (dd, *J* = 11.1, 3.7 Hz, 1H, -PhCH2O), 4.73 – 4.66 (m, 3H, -PhCH2O), 4.60 (td, *J* = 11.8, 3.4 Hz, 3H, -PhCH2O), 4.52 – 4.47 (m, 3H, H1', -PhCH2O), 4.45 (q, *J* = 3.6, 2.9 Hz, 2H, H1, -PhCH2O), 4.36 (dd, *J* = 10.9, 3.5 Hz, 1H, -PhCH2O), 4.10 (dt, *J* = 10.6, 2.6 Hz, 1H, H6A'), 4.02 (d, *J* = 3.0 Hz, 1H, H4'), 3.87 (td, *J* = 9.4, 3.5 Hz, 1H, H3), 3.63

(dddd, J = 19.0, 11.4, 7.8, 3.9 Hz, 6H, H3', H5', H5, H6B', H6A, H6B), 3.41 (dt, J = 9.8, 3.5 Hz, 1H, H2), 3.36 (td, J = 9.3, 3.7 Hz, 1H, H4), 3.20 – 3.13 (m, 3H, -CH3).

^{13}C NMR (126 MHz, $CDCl_3$) δ 165.15(C=O), 138.93(Arom-C), 138.48, 138.32, 138.23, 137.86, 137.65, 132.79, 130.15, 129.83, 128.52, 128.47, 128.39, 128.35, 128.31, 128.22, 128.09, 127.94, 127.86, 127.77, 127.68, 127.66, 127.57, 127.55, 127.46, 127.39(Arom-C), 101.69(C1'), 97.76(C1), 81.96(C3), 79.94(C3'), 79.80(C2), 77.53(C4), 75.47(-PhCH2O), 74.59(-PhCH2O), 74.55(-PhCH2O), 73.75(C5'), 73.57(-PhCH2O), 73.31(-PhCH2O), 72.57(C4'), 71.71(-PhCH2O), 69.54(C5), 68.52(C6), 67.68(C6'), 54.89(-CH3).

Synthesis of Methyl 2,3,6-tri-O-benzyl-4-O-(2-benzoyl-3,4,6-tri-O-benzyl-β-D-galactopyranosyl)-α-D-glucopyranoside(G8)

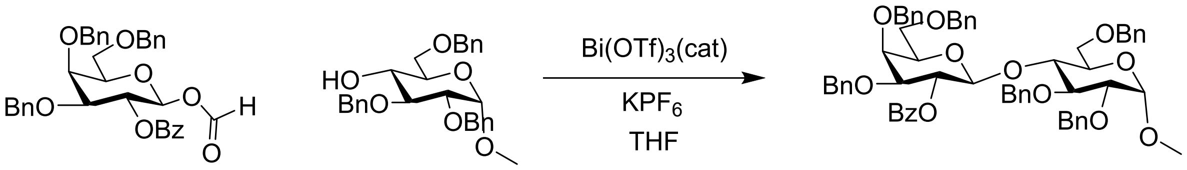

D2 (120 mg, 0.20 mmol) was dissolved in THF (3.0 mL) followed by the acceptor 2,3,6-tri-O-benzyl-α-D-glucopyranoside (105 mg, 0.23 mmol), KPF_6 (38 mg, 0.20 mmol) and $Bi(OTf)_3$ (38 mg, 0.05 mmol). After 4 hours at rt under a N_2 atmosphere the reaction was quenched with TEA, aqueous worked-up using EA and finally concentrated in vacuo. Purification by flash column chromatography on silica gel (Ethyl acetate / Heptane 1:20 – 1:10) gave a colorless oil product (75 mg, 38%, β, Rf:0.4 in Heptane/EtOAc=5/1); HRMS(ESI): $[M+Na]^+$calcd for $C_{35}H_{34}O_8Na^+$1023.4290, found 1023.4302.

^{1}H NMR (500 MHz, $CDCl_3$) δ 8.08-8.07 (m, 2H, Arom-H), 7.49-6.98 (m, 28H, Arom-H), 5.77 (dd, 1H, J = 9.5, 8.1 Hz, H2), 5.30 (d, 1H, J = 11.8 Hz.-PhCH2O), 5.07 (d, 1H, J = 8.1 Hz, H1), 4.94- 4.55 (d, 6H, J = 3.2 Hz, -PhCH2O, H1'), 4.55-4.36 (m, 5H, -PhCH2O), 4.32-4.28 (m, 2H, H4', -PhCH2O), 4.23 (t, 1H, J = 9.3 Hz, H3'), 3.93 (dd, 1H, J = 11.0, 3.7 Hz, H6A'), 3.78-3.69 (m, 4H, H-3, H-4, H6B, H-5'), 3.59-3.55 (m, 2H, H-5, H6A), 3.53 (dd, 1H, J = 11.0, 1.5 Hz, H6B'), 3.49 (dd, 1H, J = 9.3, 3.7 Hz, H2'), 2.94 (s, 3H, OMe);

^{13}C NMR (126 MHz, C6H6) δ 162.8(PhC=O), 138.6(Arom-C), 138.3, 138.1, 138.02, 138.0, 137.8, 133.0, 130.7, 130.0, 128.6, 128.6, 128.4, 128.3, 128.21, 128.1, 127.93, 127.92, 127.7, 127.61, 127.54, 127.54, 127.1(Arom-C), 97.2, 92.4, 83.5, 80.5, 80.4, 78.52, 77.7, 76.21, 75.23, 75.0, 74.91, 74.8, 73.7, 73.6, 73.1, 70.7, 69.1, 68.7, 54.8;

General procedure for the derivatives of mannose

Synthesis of Penta-O-benzoyl-D-mannopyranose(S10)

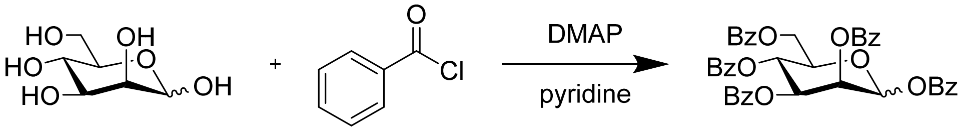

D-mannose (10.0 g, 55.5 mmol) was added to a solution of pyridine (40 mL) in dichloromethane (50 mL) at 0°C and stirred for 30 mins. Benzoyl chloride (54.6 g, 389 mmol) and DMAP (0.67 g, 5.55 mmol) were added to the mixture followed by stirring at room temp. overnight. The reaction mixture was diluted with CH_2Cl_2 (100 mL), washed with water (2 × 100 mL) and brine (100 mL). The combined organic layers were dried over Na_2SO_4 and after removal of the solvent under vacuum, the residue was purified by column chromatography (Ethyl acetate / Heptane 1/5 - 1/2) on silica gel using chloroform to afford a white solid (37.6 g, 97 %).

^{1}H NMR (500 MHz, $CDCl_3$) δ 8.25 – 8.20 (m, 2H, Arom-H), 8.15 – 8.11 (m, 2H, Arom-H), 8.01 – 7.97 (m, 2H, Arom-H), 7.91 – 7.86 (m, 2H, Arom-H), 7.71 (t, J = 7.5 Hz, 1H, Arom-H), 7.66 (d, J = 7.9 Hz, 1H, Arom-H), 7.59 (ddd, J = 7.8, 5.8, 2.8 Hz, 3H, Arom-H), 7.54 (t, J = 7.4 Hz, 1H, Arom-H), 7.50 – 7.28 (m, 11H, Arom-H), 6.66 (d, J = 2.1 Hz, 1H, -α-H1), 6.31 (t, J = 10.1 Hz, 1H, -H4), 6.10 (dd, J = 10.2, 3.3 Hz, 1H, H3), 5.96 – 5.91 (m, 1H, H2), 4.73 (dd, J = 12.3, 2.6 Hz, 1H, H6A), 4.60 (dt, J = 10.0, 3.2 Hz, 1H, H5), 4.53 (dd, J = 12.3, 3.7 Hz, 1H, H6B).

^{13}C NMR (126 MHz, $CDCl_3$) δ 166.06(C=O), 165.70(C=O), 165.31(C=O), 165.16(C=O), 163.84(C=O), 134.09(Arom-C), 133.68(Arom-C), 133.56, 133.42, 133.07, 130.17, 129.98, 129.83, 129.79, 129.02, 128.84, 128.82, 128.73, 128.69, 128.49, 128.45, 128.42(Arom-C), 91.40(C1), 71.21(C5), 70.01(C3), 69.45(C2), 66.19(C4), 62.36(C6).

Synthesis of 2,3,4,6-Tetra-O-benzoyl-α-D-mannopyranosyl bromide(S11)[9]

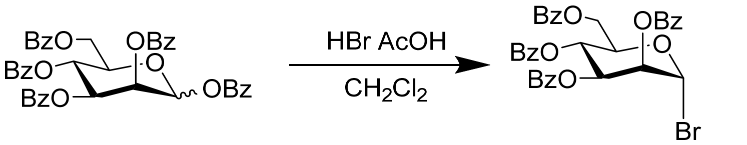

HBr (1.6 mL, 6.42 mmol, 33% in AcOH) was added dropwise to compound **S10** (980 mg, 1.28 mmol) dissolved in DCM at 0 °C and then stirred for 0.5 h before it was allowed to reach room temperature, where it was stirred for another 3 h. The reaction mixture was diluted with dichloromethane (20 mL) and washed

with cold water (20 mL) and a cold saturated $NaHCO_3$ solution (10 mL). The organic layer was dried over Na_2SO_4 and the solvent was removed by rotary evaporation to obtain a brownish white solid (780 mg, 88 %).

^{1}H NMR (500 MHz, $CDCl_3$) δ 8.20 – 7.25 (m, 20H, arom-H), 6.62 (d, J = 1.7 Hz, 1H, H1), 6.35 – 6.23 (m, 2H, H3, H4), 5.94 (dd, J = 3.0, 1.8 Hz, 1H, H2), 4.77 (dd, J = 12.6, 2.5 Hz, 1H, H6A), 4.69 (dt, J = 9.6, 3.1 Hz, 1H, H5), 4.54 (dd, J = 12.5, 3.8 Hz, 1H, H6B).

^{13}C NMR (126 MHz, $CDCl_3$) δ 165.96, 165.38, 165.30, 164.99(C=O), 133.79, 133.69, 133.41, 133.21(arom-C), 129.90, 129.81, 129.78, 129.66, 128.79, 128.73, 128.71, 128.67, 128.55, 128.51, 128.43, 128.40(arom-C), 83.29(C1), 73.13(C2), 72.96(C5), 69.10(C3), 65.93(C4), 61.77(C6).

Synthesis of 1,2-O-(α-methoxybenzylidene)-3,4,6-tri-O-benzoyl-β-D-mannopyranose (S12)[10]

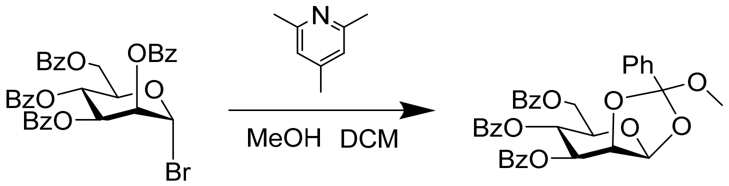

To a solution of 2,3,4,6-O-tetrabenzoyl-α-D-mannopyranosyl bromide **S11** (8 g, 12.13 mmol) in CH_2Cl_2 (39 mL) was added MeOH (1.93 mL) and 2,6-lutidine (1.9 mL, 17 mmol) and the reaction was stirred at room temperature for 3 d. The solution was then quenched with water (20 mL) and diluted with Et_2O (100 mL). The combined organic layers were washed with brine, dried over Na_2SO_4 and the solvent removed under reduced pressure. The resulting clear oil was further dried for 2 d under high vacuum to give 1,2-O-(α-methoxybenzylidene)-3,4,6-tri-O-benzoyl-β-D-mannopyranose as a white-foam that was used without further purification.

^{1}H NMR (500 MHz, $CDCl_3$) δ 8.07 – 7.27 (m, 20H, arom-H), 5.92 (t, J = 9.6 Hz, 1H, H4), 5.83 (d, J = 3.0 Hz, 1H, H1), 5.68 (dd, J = 10.0, 4.1 Hz, 1H, H3), 5.10 (dd, J = 4.1, 2.9 Hz, 1H, H2), 4.58 – 4.49 (m, 1H, H6A), 4.38 (dd, J = 12.1, 4.8 Hz, 1H, H6B), 4.19 – 4.08 (m, 1H, H5), 3.26 (s, 3H, -CH3).

^{13}C NMR (126 MHz, $CDCl_3$) δ 166.06, 166.01, 165.17(C=O), 136.19, 133.50, 133.41, 132.98, 130.05, 129.79, 129.73, 129.59, 129.32, 128.98, 128.92, 128.44, 128.30, 128.21, 126.48, 123.15(arom-C), 97.92(C1), 76.32(C2), 72.10(C5), 71.17(C3), 66.47(C4), 63.11(C6), 51.49(-CH_3).

The synthesis of 1,2-O-(Methoxyphenylmethylene)-3,4,6-tri-O-benzyl-α-D-mannopyranose**(S13)**

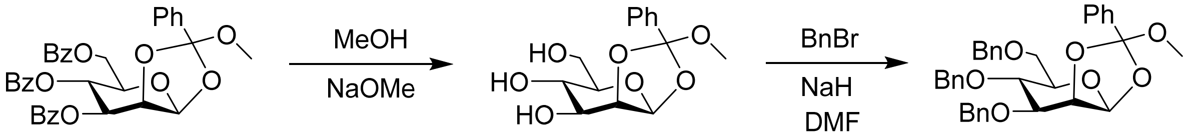

1,2-*O*-(Methoxybenzylidene)-3,4,6-tri-*O*-benzoyl-β- D-mannopyranose was dissolved in CH_2Cl_2 (6.2 mL) to which MeOH (23 mL) and NaOMe (2 mL of a 0.5 M solution in MeOH) were added slowly. The resulting solution was stirred at room temperature for 24 h. The solvent was then removed under reduced pressure and the residue was dissolved in DMF, where NaH (0.98 g, 40 mmol) was added in portions. To this stirring solution was then slowly added benzyl bromide (5.71 g, 33.8 mmol) over 30 min, and the reaction was left stirring at rt for 16 h. The reaction was quenched with methanol, extracted by EA (50 mL) and washed with water (100 mL), then concentrated in vacuo. The residue was purified by flash column chromatography on silica gel (1:10 to 1:5 EA/Heptane) yielding (3.60 g, 62%, β, Rf: 0.6 in Heptane/EtOAc=5/1); HRMS(ESI): $[M+Na]^+$calcd for $C_{35}H_{36}O_7Na^+$ 591.2361, found 591.2353.

^{1}H NMR (500 MHz, $CDCl_3$) δ 7.74 – 7.70 (m, 2H, Arom-H), 7.48 – 7.45 (m, 2H, Arom-H), 7.40 – 7.23 (m, 16H, arom-H), 5.53 (d, *J* = 3.0 Hz, 1H, H1), 4.94 – 4.85 (m, 3H, -PhCH2O), 4.71 (dd, *J* = 4.0, 3.0 Hz, 1H, H2), 4.66 (d, *J* = 10.8 Hz, 1H, -PhCH2O), 4.47 – 4.40 (m, 2H, -PhCH2O), 3.97 (t, *J* = 9.1 Hz, 1H, H4), 3.88 (dd, *J* = 9.3, 3.9 Hz, 1H, H3), 3.69 (dd, *J* = 10.9, 4.9 Hz, 1H, H6A), 3.60 (dd, *J* = 10.9, 2.5 Hz, 1H, H6B), 3.53 (ddd, *J* = 9.1, 4.9, 2.5 Hz, 1H, H5), 3.31 (s, 3H, -CH3).

^{13}C NMR (126 MHz, $CDCl_3$) δ 138.40(Arom-C), 138.29, 137.93, 136.63, 129.06, 128.54, 128.40, 128.26, 128.08, 128.02, 128.00, 127.98, 127.76, 127.50, 127.41, 126.78(Arom-C), 122.54(-PhC-), 97.87(C1), 78.52(C3), 76.35(C2), 75.09(C5, -PhCH2O), 74.35(C4), 73.24(-PhCH2O), 72.11(-PhCH2O), 69.16(C6), 51.41(-CH3).

Synthesis of Formyl-2-O-benzoyl-3,4,6-tri-O-benzyl-α-D-mannopyranoside**(D3)**

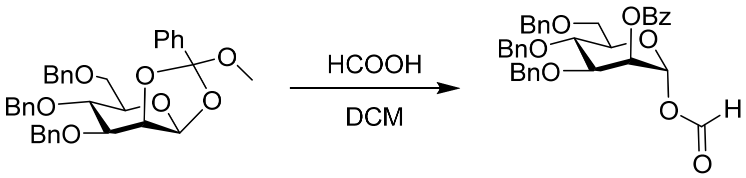

The 1,2-orthoester **S13** (500 mg, 0.88 mmol) was dissolved into DCM (10 mL) , then formic acid (56 mg, 1.14 mmol) was added at rt. After 2 h, the reaction mixture was concentrated in vacuo. The residue was purified by flash column chromatography on silica gel (EA / Heptane 1/10 - 1/5) affording a colorless oil (434 mg, 86%, Rf:0.41 in Heptane/EtOAc=4/1); HRMS(ESI): $[M+Na]^+$ calcd for $C_{35}H_{34}O_8Na^+$ 605.2146, found 605.2179.

^{1}H NMR (500 MHz, $CDCl_3$) δ 8.16 – 8.06 (m, 3H, -CHO, Arom-H), 7.64 – 7.59 (m, 1H, arom-H), 7.50 – 7.19 (m, 17H), 6.40 (d, *J* = 2.5 Hz, 1H, H1), 5.69 (p, *J* = 3.0 Hz, 1H, H2), 4.95 (dd, *J* = 10.8, 2.4 Hz, 1H, -PhCH2O), 4.86 (dd, *J* = 11.5, 2.4 Hz, 1H, -PhCH2O), 4.77 (dd, *J* = 12.0, 2.4 Hz, 1H, -PhCH2O), 4.68 – 4.56 (m, 3H, -PhCH2O), 4.24 (qd, *J* = 8.9, 8.1, 2.5 Hz, 1H, H4), 4.16 (dt, *J* = 9.5, 2.8 Hz, 1H, H3), 4.03 – 3.92 (m, 2H, H5, -H6A), 3.82 (dd, *J* = 11.2, 2.6 Hz, 1H, H6B).

^{13}C NMR (126 MHz, $CDCl_3$) δ 165.43(C=O), 158.55(CHO), 138.35(Arom-C), 138.18, 137.67, 133.44, 130.08, 129.46, 128.54, 128.43, 128.41, 128.08, 128.04, 127.84, 127.80, 127.61, 127.59(Arom-C), 91.48(C1), 77.59(C3), 75.43(-PhCH2O), 74.27(C5), 73.59(C4), 73.57(-PhCH2O), 71.83(-PhCH2O), 68.62(C6), 67.77(C2).

The synthesis of 1-Cyclohexyl-2-O-(benzoyl-3,4,6-tri-O-benzyl-D-mannopyranoside(**G9)**

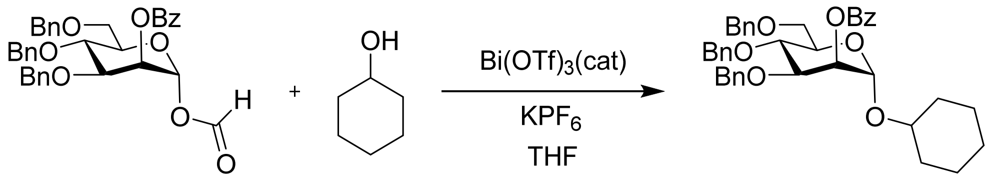

The starting materials formyl-2-O-benzoyl-3,4,6-tri-O-benzyl-β-D-glucopyranoside **D3** (120 mg, 0.20 mmol) was dissolved in THF (3.0 mL). Then, Cyclohexanol (23 mg, 0.23 mmol), KPF_6 (41 mg, 0.23 mmol) and $Bi(OTf)_3$ (38 mg, 0.05 mmol) were added. After 4 hours at rt under a N_2 atmosphere, the reaction was quenched with TEA, aqueous worked-up using EA and finally concentrated in vacuo. Purification by flash column chromatography on silica gel (Ethyl acetate / Heptane 1:20 – 1:10) gave a colorless oil product (81 mg, 62%, α, Rf:0.5 in Heptane/EtOAc=5/1); $[\alpha]^{25}_{D}$: 8(c=1, $CHCl_3$). HRMS(ESI): $[M+Na]^+$ calcd for $C_{35}H_{34}O_8Na^+$ 659.2979, found 659.2993.

^{1}H NMR (500 MHz, $CDCl_3$) δ 8.19 – 8.08 (m, 2H, -Arom-H), 7.61 – 7.57 (m, 1H, -Arom-H), 7.46 – 7.24 (m, 17H, -Arom-H), 5.66 – 5.62 (m, 1H, H2), 5.17 (d, *J* = 1.9 Hz, 1H, H1), 4.93 (d, *J* = 10.7 Hz, 1H, PhCH2O-), 4.86 (d, *J* = 11.2 Hz, 1H, $PhCH_2O$-), 4.80 (d, *J* = 12.0 Hz, 1H, $PhCH_2O$-), 4.64 (d, *J* = 11.2 Hz, 1H, $PhCH_2O$-), 4.59 (d, *J* = 11.3 Hz, 2H, $PhCH_2O$-), 4.20 (dd, *J* = 9.3, 3.1 Hz, 1H, H4), 4.15 (t, *J* = 9.4 Hz, 1H, H3), 4.02 (ddd, *J* = 9.6, 4.0, 1.8 Hz, 1H, H5), 3.97 (dd, *J* = 10.7, 4.0 Hz, 1H, H6A), 3.83 (dd, *J* = 10.7, 1.8 Hz, 1H, H6B), 3.70 (ddt, *J* = 13.0, 8.9, 3.8 Hz, 1H, -OCH-), 1.97 – 1.89 (m, 2H, -CH_2-), 1.77 (dt, *J* = 10.8, 3.5 Hz, 2H, -CH_2-), 1.58 – 1.43 (m, 2H, -CH_2-), 1.40 – 1.25 (m, 4H, -CH_2-).

^{13}C NMR (126 MHz, $CDCl_3$) δ 165.90(-C=O), 138.58(Arom-C), 138.45, 138.18, 133.10, 130.07, 130.00, 128.59, 128.41, 128.38, 128.36, 128.32, 128.13, 128.07, 127.70, 127.60, 127.51, 127.48(Arom-C), 95.90(C1), 78.44(C4), 75.66(C3), 75.40(-OCH-), 74.61($PhCH_2O$-), 73.43(C3), 71.65($PhCH_2O$-),

71.60(C5), 69.77($PhCH_2O$-), 69.23(C6), 33.28(-CH_2-), 31.44(-CH_2-), 25.64(-CH_2-), 24.07(-CH_2-), 23.82(-CH_2-).

The synthesis of adamantyl 2-O-benzoyl-(3,4,6-tri-O-benzyl)-D-mannopyranoside**(G10)**

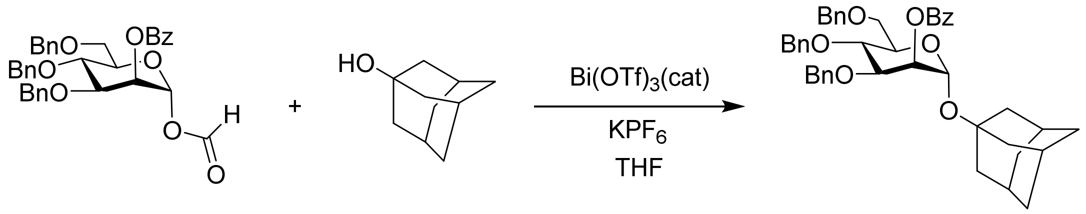

Formyl 2-O-benzoyl-3,4,6-tri-O-benzyl-β-D-glucopyranoside **D3** (120 mg, 0.20 mmol) was dissolved in THF (3.0 mL). Then, 1-adamantanol (34 mg, 0.23 mmol), KPF_6 (38 mg, 0.20 mmol) and $Bi(OTf)_3$ (38 mg, 0.05 mmol) were added. After 4 hours at rt under a N_2 atmosphere, the reaction was quenched with TEA, aqueous worked-up using EA and finally concentrated in vacuo. Purification by flash column chromatography on silica gel (Ethyl acetate / Heptane 1:20 – 1:10) gave a colorless oil (96 mg, 68%, α-product, Rf:0.5 in Heptane/EtOAc=5/1); $[\alpha]_D^{25}$: 1 (c=1, $CHCl_3$). HRMS(ESI): $[M+Na]^+$calcd for $C_{35}H_{34}O_8Na^+$ 711.3292, found 711.3312.

^{1}H NMR (500 MHz, $CDCl_3$) δ 8.14 – 8.09 (m, 2H, Arom-H), 7.59 – 7.55 (m, 1H), 7.43 – 7.20 (m, 17H, Arom-H), 5.47 – 5.44 (m, 1H, H2), 5.42 (d, J = 2.2 Hz, 1H, H1), 4.91 (d, J = 10.6 Hz, 1H, -PhCH2O), 4.80 (dd, J = 23.6, 11.6 Hz, 2H, -PhCH2O), 4.61 (d, J = 11.3 Hz, 1H, -PhCH2O), 4.55 (dd, J = 11.3, 4.8 Hz, 2H, -PhCH2O), 4.23 – 4.19 (m, 1H, H3), 4.16 – 4.09 (m, 2H, H4, H5), 3.94 (dd, J = 10.6, 3.2 Hz, 1H, H6A), 3.79 (dd, J = 10.6, 1.5 Hz, 1H, H6B), 2.16 (p, J = 3.1 Hz, 3H, -CH-, -CH-, -CH-), 1.85 (m, J = 3.6, 1.8 Hz, 6H, -CH2-, -CH2-, -CH2-), 1.66 – 1.58 (m, 6H, -CH2-, -CH2-, -CH2-).

^{13}C NMR (126 MHz, $CDCl_3$) δ 166.01(C=O), 138.60(Arom-H), 138.51, 138.23, 133.03, 130.13, 129.98, 128.37, 128.34, 128.29, 128.09, 128.06, 127.64, 127.56, 127.49, 127.41(Arom-H), 91.06(C1), 78.35(C3), 75.35(-C-O-), 75.14(-PhCH2O), 74.76(C4), 73.39 (-PhCH2O), 71.52(-PhCH2O) , 71.19(C5), 70.70(C2), 69.28(C6), 42.33(-CH-, -CH-, -CH-), 36.24(-CH2-, -CH2-, -CH2-), 30.64(-CH2-, -CH2-, -CH2-)

The synthesis of Methyl 2,3,4-tri-O-benzyl-6-O-(2-benzoyl-3,4,6-tri-O-benzyl-β-D-mannopyranosyl)-α-D-glucopyranoside(G11)

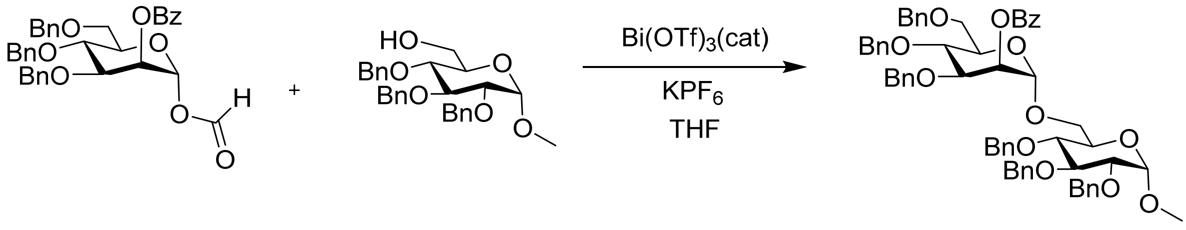

Formyl 2-O-benzoyl-3,4,6-tri-O-benzyl-β-D-glucopyranoside **D3** (120 mg, 0.20 mmol) was dissolved in THF (3.0 mL). Then, methyl 2,3,4-tri-O-benzyl-α-D-glucopyranoside (105 mg, 0.23 mmol), KPF_6 (38 mg, 0.20 mmol) and $Bi(OTf)_3$ (38 mg, 0.05 mmol) were added. After 4 hours at rt under a N_2 atmosphere, the reaction was quenched with TEA, aqueous worked-up using EA and finally concentrated in vacuo. Purification by flash column chromatography on silica gel (Ethyl acetate / Heptane 1:20 – 1:10) gave a colorless oil (146 mg, 71%, α, Rf:0.45 in Heptane/EtOAc=5/1); $[\alpha]_D^{25}$:23 (c=1, $CHCl_3$). HRMS(ESI): $[M+Na]^+$calcd for $C_{35}H_{34}O_8Na^+$1023.4290, found 1023.4295.

^{1}H NMR (500 MHz, $CDCl_3$) δ 8.12 – 8.08 (m, 2H, Arom-H), 7.58 (td, J = 7.4, 1.4 Hz, 2H, Arom-H), 7.44 – 7.19 (m, 31H), 5.69 (t, J = 2.4 Hz, 1H, H2'), 5.07 (d, J = 2.0 Hz, 1H, H1'), 5.04 (d, J = 10.8 Hz, 1H, -$PhCH_2O$), 4.92 (dd, J = 11.0, 7.1 Hz, 2H, -$PhCH_2O$), 4.86 – 4.80 (m, 3H, -$PhCH_2O$), 4.73 (dd, J = 12.0, 10.3 Hz, 2H, -PhCH2O), 4.64 (d, J = 3.6 Hz, 1H, H1), 4.60 (d, J = 11.5 Hz, 1H, -$PhCH_2O$), 4.57 – 4.53 (m, 2H, -$PhCH_2O$), 4.49 (d, J = 12.0 Hz, 1H, -$PhCH_2O$), 4.10 (d, J = 8.8 Hz, 1H, H3'), 4.09 – 4.06 (m, 1H, H4'), 4.03 (t, J = 9.3 Hz, 1H, H3), 3.89 (dd, J = 11.4, 4.4 Hz, 1H, H6A'), 3.83 – 3.77 (m, 3H, H5', H5, H6A), 3.69 (ddd, J = 16.1, 11.1, 1.8 Hz, 2H, H6B', H6B), 3.61 (dd, J = 9.6, 3.5 Hz, 1H, H2), 3.51 (dd, J = 10.0, 8.9 Hz, 1H, H4), 3.36 (s, 3H, -CH_3).

^{13}C NMR (126 MHz, $CDCl_3$) δ 165.60(C=O), 138.74(Arom-C), 138.57, 138.49, 138.23, 138.15, 137.86, 133.11, 130.00, 128.51, 128.43, 128.40, 128.35, 128.33, 128.26, 128.18, 128.15, 128.05, 127.95, 127.87, 127.69, 127.65, 127.59, 127.53, 127.50, 127.46(Arom-C), 98.13(C1'), 97.85(C1), 82.12(C3), 80.13(C2), 77.64(C4'), 77.60(C4), 75.80(-$PhCH_2O$), 75.18(-$PhCH_2O$), 74.97(-$PhCH_2O$), 74.22(C3'), 73.37(-2$PhCH_2O$), 71.75(C5'), 71.35(-$PhCH_2O$), 69.74(C5), 68.93(C2'), 68.75(C6), 66.12(C6'), 55.14(-CH3).

The synthesis of Methyl 2,3,6-tri-O-benzyl-4-O-(2-benzoyl-3,4,6-tri-O-benzyl-β-D-mannopyranosyl)-α-D-glucopyranoside(G12)

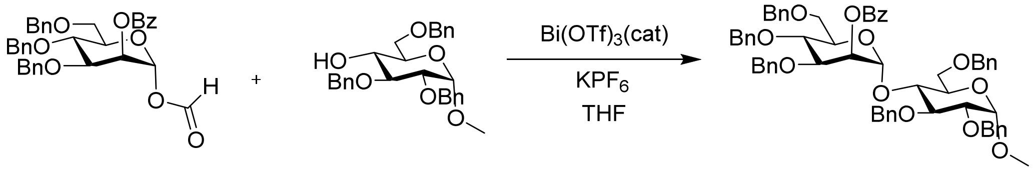

Formyl-2-O-benzoyl-3,4,6-tri-O-benzyl-β-D-glucopyranoside **D3** (120 mg, 0.20 mmol) was dissolved in THF (3.0 mL). Then, methyl 2,3,6-tri-O-benzyl-α-D-glucopyranoside (105.3 mg, 0.23 mmol), KPF_6 (38 mg, 0.20 mmol) and $Bi(OTf)_3$ (38 mg, 0.05 mmol) were added. After 4 hours at rt under a N_2 atmosphere, the reaction was quenched with TEA, aqueous worked-up using EA and finally concentrated in vacuo. Purification by flash column chromatography on silica gel (Ethyl acetate / Heptane 1:20 – 1:10), gave a colorless oil (157 mg, 76%, α, Rf:0.43 in Heptane/EtOAc=5/1); $[\alpha]_D^{25}$: -12(c=1, $CHCl_3$). HRMS(ESI): $[M+Na]^+$calcd for $C_{35}H_{34}O_8Na^+$1023.4290, found 1023.4270.

^{1}H NMR (500 MHz, $CDCl_3$) δ 8.07 – 7.96 (m, 2H, Arom-H), 7.56 (dd, *J* = 8.2, 6.7 Hz, 1H), 7.38 – 7.08 (m, 32H, Arom-H), 5.73 (t, *J* = 2.5 Hz, 1H, H1'), 5.58 (d, *J* = 2.1 Hz, 1H, H2'), 5.05 (d, *J* = 10.9 Hz, 1H, -PhCH2O), 4.88 – 4.43 (m, 12H, -PhCH2O, H1), 4.10 – 4.02 (m, 2H, H3', H4), 3.98 (t, *J* = 9.1 Hz, 1H, H3), 3.91 – 3.84 (m, 2H, H4', H5), 3.79 – 3.70 (m, 4H, H5', H6A', H6B', H6A), 3.60 – 3.55 (m, 2H, H6B, H2), 3.42 (s, 3H, -CH3).

^{13}C NMR (126 MHz, $CDCl_3$) δ 165.26(C=O), 138.53(Arom-C), 138.51, 138.30, 138.18, 138.08, 137.98, 132.97, 129.98, 128.53, 128.48, 128.32, 128.28, 128.25, 128.21, 128.18, 128.03, 127.99, 127.97, 127.60, 127.57, 127.52, 127.50, 127.47, 127.43, 127.33(Arom-C), 99.25(C1'), 97.82(C1), 81.73(C3), 80.18(C2), 78.20(C3'), 75.73(C5), 75.43(-PhCH2O), 75.26(-PhCH2O), 74.12(C4), 73.45(-PhCH2O), 73.36(-PhCH2O), 73.30(-PhCH2O), 72.70(C4'), 71.44(-PhCH2O), 69.62(C6'), 69.24(C2'), 69.13(C5'), 68.97(C6), 55.32(-CH3).

General procedure for the derivatives of 2-amino-2-deoxy-D-glucose

The synthesis of 2-N-alloc-2-deoxy-D-glucopyranose (S14)[11,12]

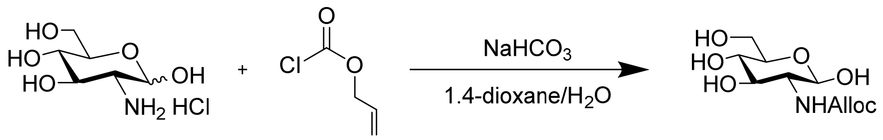

D-Glucosamine hydrochloride (1.00 g, 4.64 mmol, 1.0 eq) and sodium bicarbonate (1.36 g, 16.2 mmol, 3.5 eq) were dissolved in 1,4-dioxane (37.5 mL) and MilliQ water (12.5 mL). Thereafter alloc chloride (5.57 mmol, 1.2 eq) was added dropwise and the reaction was stirred until complete conversion (within 1 h. as indicated by TLC). The solvents were evaporated and the crude product was purified by column chromatography (0 - 20 % MeOH/DCM, v/v) yielding the product as a white powder.

^{1}H NMR (500 MHz, MeOD) δ 5.96 (ddtd, *J* = 18.1, 10.7, 5.4, 2.3 Hz, 1H, -CH=CH2), 5.33 (dq, *J* = 17.3, 1.8 Hz, 1H, -CH=CH2), 5.19 (ddd, *J* = 10.5, 3.1, 1.6 Hz, 1H, -CH=CH2), 5.14 (d, *J* = 3.5 Hz, 1H, H1), 4.61 – 4.50 (m, 2H, -CH2-CH-), 3.81 (dt, *J* = 11.1, 2.5 Hz, 2H, H5, H6A), 3.75 – 3.69 (m, 1H, H6B), 3.69 – 3.64 (m, 1H, H3), 3.58 (dd, *J* = 10.6, 3.5 Hz, 1H, H2), 3.38 (t, *J* = 9.2 Hz, 1H, H4).

^{13}C NMR (126 MHz, MeOD) δ 158.76(-C=O), 134.45(-CH=CH2, 117.60(-CH=CH2), 92.97(C1), 73.09(C3), 72.92(C4), 72.41(C5), 66.57(CH2-CH-, 62.82(C6), 57.56(C2).

The synthesis of 2-N-Alloc-2-deoxy-1,3,4,6-tetraacetyl-D-glucopyranose(S15)[13,14]

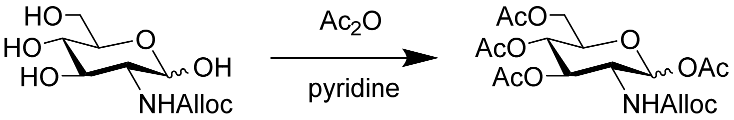

Under a N_2 atmosphere, the starting material **S14** (5.0 g, 17.9 mmol) was dissolved in pyridine (30 mL), then Ac_2O (18.3 g, 179.05 mmol) was added while cooling on an ice bath. After 12 hours, the reaction was concentrated in vacuo and worked up using EA. Purification by flash column chromatography on silica gel (Ethyl acetate / Heptane 1:5 – 1:2), gave a colorless oil product (7.68 g, 79%, Rf:0.63 in Heptane/EtOAc=1/1);

^{1}H NMR (500 MHz, $CDCl_3$) δ 6.21 (d, *J* = 3.6 Hz, 1H, H1), 5.93 (ddt, *J* = 16.3, 10.8, 5.7 Hz, 1H, -CH=CH2), 5.37 – 5.21 (m, 2H, -CH=CH2), 5.00 (t, *J* = 9.7 Hz, 1H, H4), 4.60 (d, *J* = 5.7 Hz, 2H, -CH2-CH), 4.31 (dd, *J* = 12.5, 4.5 Hz, 1H, H6A), 4.20 – 4.04 (m, 2H, H6B, H2), 3.98 (ddd, *J* = 10.3, 4.4, 2.3 Hz, 1H, H3), 3.87 – 3.75 (m, 1H, H5), 2.20 – 2.06 (m, 12H, -CH3, -CH3, -CH3, -CH3).

^{13}C NMR (126 MHz, $CDCl_3$) δ 171.32 (C=O), 170.67, 169.18, 168.66, 155.41 (C=O), 132.31(CH=CH2), 118.15(CH=CH2), 90.80(C1), 70.62(C4), 69.69(C2), 67.63(C3), 66.12(CH2-CH-), 61.54(C6), 52.85(C5), 20.92(CH3), 20.73(CH3), 20.70(CH3), 20.56(CH3).

The synthesis of 2-deoxy-2-[[(2-propenyloxy)carbonyl]amino]-, 3,4,6-triacetate(S16)[15, 16]

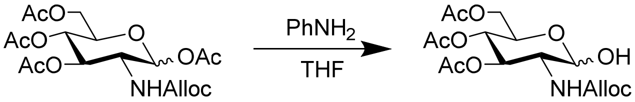

To compound **S15** (8.6 g, 16.5 mmol), dissolved in anhydrous THF (50 mL) at room temperature, benzylamine (2.2 mL, 19.8 mmol) was added slowly. Within 12 h the starting material was completely consumed (TLC control). After removal of the solvent under reduced pressure, an excess of aq 1 M HCl was added to the residue and the suspension was extracted with $CHCl_3$. The combined organic layers were washed with saturated aq $NaHCO_3$, followed by brine and the organic phase was concentrated under reduced pressure. Purification using silica-gel chromatography (Heptane/EA, 5:1-2/1) afforded a colorless oil product (167 mg, 83%, α, Rf:0.43 in Heptane/EtOAc=5/1);

^{1}H NMR (500 MHz, $CDCl_3$) δ 5.89 (ddt, *J* = 16.2, 10.7, 5.6 Hz, 1H, CH=CH2), 5.34 – 5.18 (m, 4H, CH=CH2, H1, H3), 5.10 (dt, *J* = 17.2, 9.6 Hz, 1H, H4), 4.62 – 4.47 (m, 2H, -CH2-CH=), 4.28 – 4.11 (m, 3H, H5, H6A, H6B), 4.01 (td, *J* = 10.3, 3.7 Hz, 1H, H2), 2.10 (s, 3H, -CH3), 2.03 (d, *J* = 4.9 Hz, 6H, -CH3, -CH3).

^{13}C NMR (126 MHz, $CDCl_3$) δ 171.19(C=O), 170.96, 169.50, 155.82(C=O), 132.51(-CH=CH2), 117.85(-CH=CH2), 91.87(C1), 70.95(C3), 68.40(C4), 67.57(C5), 65.84(-CH=CH2), 62.11(C6), 53.94(C2), 20.77(-CH3), 20.74(-CH3), 20.64(-CH3).

Synthesis of formyl 2-deoxy-2-N-Alloc-3,4,6-tri-O-acetyl D-glucopyranose (D4)

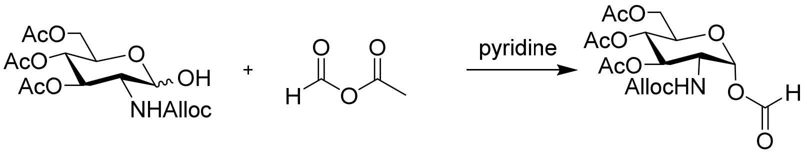

Formic acid was added slowly to Ac_2O cooled on an ice bath, where after the reaction was kept for 2 h at 55°C. After that the crude mixture was added to the starting material **D4** (0.5 g, 1.28 mmol) in pyridine (30 mL). After 12 hours, the reaction was concentrated in vacuo and aqueous worked-up using EA and finally concentrated in vacuo. Purified by flash column chromatography on silica gel (Ethyl acetate / Heptane 1:5 – 1:2), giving a colorless oil product (0.37g g, 87% brsm, Rf:0.57 in Heptane/EtOAc=1/1);

^{1}H NMR (500 MHz, $CDCl_3$) δ 8.20 (s, 1H, -CHO), 6.32 (d, J = 3.6 Hz, 1H, H1), 5.99 – 5.85 (m, 1H, -CH=CH2), 5.36 – 5.15 (m, 4H-CH=CH2, , H3, H4), 4.66 – 4.49 (m, 2H, -CH2-CH=), 4.34 – 4.19 (m, 2H, H2, H6A), 4.13 – 3.99 (m, 2H, H6B, H5), 2.11 (d, J = 1.5 Hz, 3H, -CH3), 2.06 (dd, J = 5.1, 2.2 Hz, 6H, -CH3, -CH3).

^{13}C NMR (126 MHz, $CDCl_3$) δ 171.37(C=O), 170.75(C=O), 169.27(C=O), 158.74(C=O), 155.46(CHO), 132.20(-CH=CH2), 118.22(-CH=CH2), 90.93(C1), 70.21(C3), 70.06(C5), 67.28(C4), 66.19(-CH2-CH=), 61.40(C6), 52.91(C2), 20.77(CH3), 20.75(CH3), 20.63(CH3).

Reference

1. Ekborg, G. & Glaudemans, C. P. J. p-(Trifluoroacetamido)phenyl 2-acetamido-2-deoxy-4-O-β-d-mannopyranosyl-β-d-glucopyranoside. *Carbohydr. Res.* **129**, 287–292 (1984).

2. Hölemann, A., Stocker, B. L. & Seeberger, P. H. Synthesis of a core arabinomannan oligosaccharide of Mycobacterium tuberculosis. *J. Org. Chem.* **71**, 8071–8088 (2006).

3. Yang, L., Hammelev, C. H. & Pedersen, C. M. Catalytic and Atom-Economic Glycosylation using Glycosyl Formates and Cheap Metal Salts. *ChemSusChem* **13**, 3166–3171 (2020).

4. Ma, T. *et al.* Indium(III) Iodide-Catalyzed Stereoselective Synthesis of β-Glucopyranosides by Using a Glucosyl Fluoride Donor with 2- O -Benzoyl-3,4,6-Tri- O -Benzyl Protection. *Synlett* **28**, 2633–2636 (2017).

5. Ravidà, A., Liu, X., Kovacs, L. & Seeberger, P. H. Synthesis of glycosyl phosphates from 1,2-orthoesters and application to in situ glycosylation reactions. *Org. Lett.* **8**, 1815–1818 (2006).

6. Mydock, L. K. & Demchenko, A. V. Superarming the S-benzoxazolyl glycosyl donors by simple 2-O-benzoyl-3,4,6-tri-O-benzyl protection. *Org. Lett.* **10**, 2103–2106 (2008).

7. Buhl, M. *et al.* On surface: O -glycosylation by catalytic microcontact printing. *Chem. Commun.* **53**, 6203–6206 (2017).

8. Raposo, C. D. *et al.* Development of novel galactosylated PLGA nanoparticles for hepatocyte targeting using molecular modelling. *Polymers (Basel).* **12**, (2020).

9. Kramer, S. *et al.* HPMA-Based Nanocarriers for Effective Immune System Stimulation. *Macromol. Biosci.* **19**, 1–10 (2019).

10. Wei, S., Zhao, J. & Shao, H. A facile method for the preparation of sugar orthoesters promoted by anhydrous sodium bicarbonate. *Can. J. Chem.* **87**, 1733–1737 (2009).

11. Bloemendal, V. R. L. J. *et al.* Chemoenzymatic Synthesis of Sialic Acid Derivatives Using Immobilized N-Acetylneuraminate Lyase in a Continuous Flow Reactor. *Adv. Synth. Catal.* **361**, 2443–2447 (2019).

12. Lv, Y. M. *et al.* Highly efficient and selective biocatalytic production of glucosamine from chitin.

Green Chem. **19**, 527–535 (2017).

13. Späte, A. K., Schart, V. F., Schöllkopf, S., Niederwieser, A. & Wittmann, V. Terminal Alkenes as Versatile Chemical Reporter Groups for Metabolic Oligosaccharide Engineering. *Chem. - A Eur. J.* **20**, 16502–16508 (2014).

14. Boullanger, P., Banoub,' A N D G ~ R, J., Descotes, A. R. D., Banoub, J. & Descotes, G. N-nAllyloxycarbonyl derivatives of D-glucosamine as promotors of 1,2-trans-glucosylation in Koenigs-Knorr reactions and in Lewis acid catalyzed condensatios. *Can. J. Chem* **65**, 1343 (1987).

15. De Nisco, M., Pedatella, S., Bektaş, S., Nucci, A. & Caputo, R. D-Glucosamine in a chimeric prolinamide organocatalyst for direct asymmetric aldol addition. *Carbohydr. Res.* **356**, 273–277 (2012).

16. Heinemann, F., Hiegemann, M. & Welzel, P. Glycosylations with tetra-O-acetyl-N-allyloxycarbonylamino-2-deoxy-β-D-glucose in polar solvents. *Tetrahedron* **48**, 3781–3788 (1992).

Copies of NMR spectra

^{1}H NMR (500 MHz, $CDCl_3$)

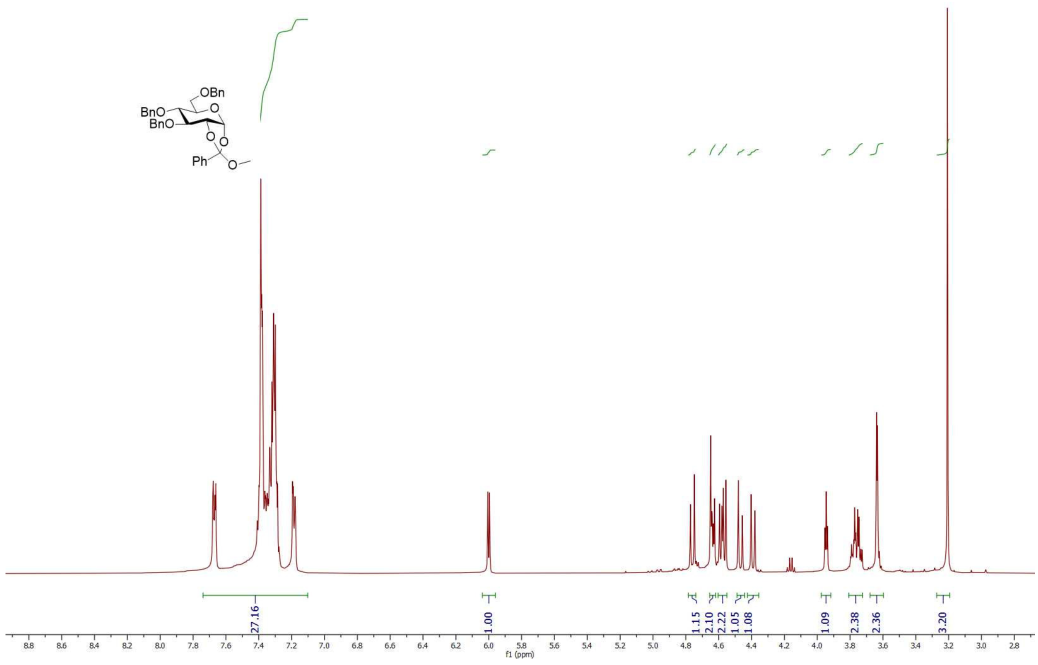

^{13}C NMR (126 MHz, $CDCl_3$)

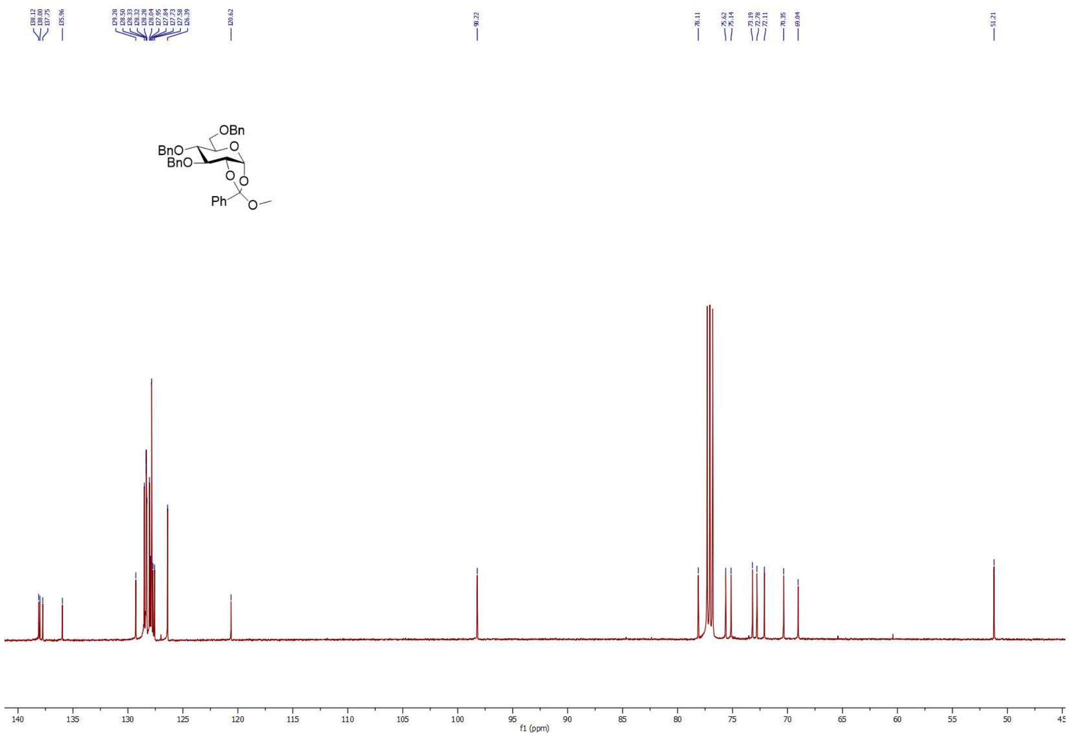

H-H COSY NMR

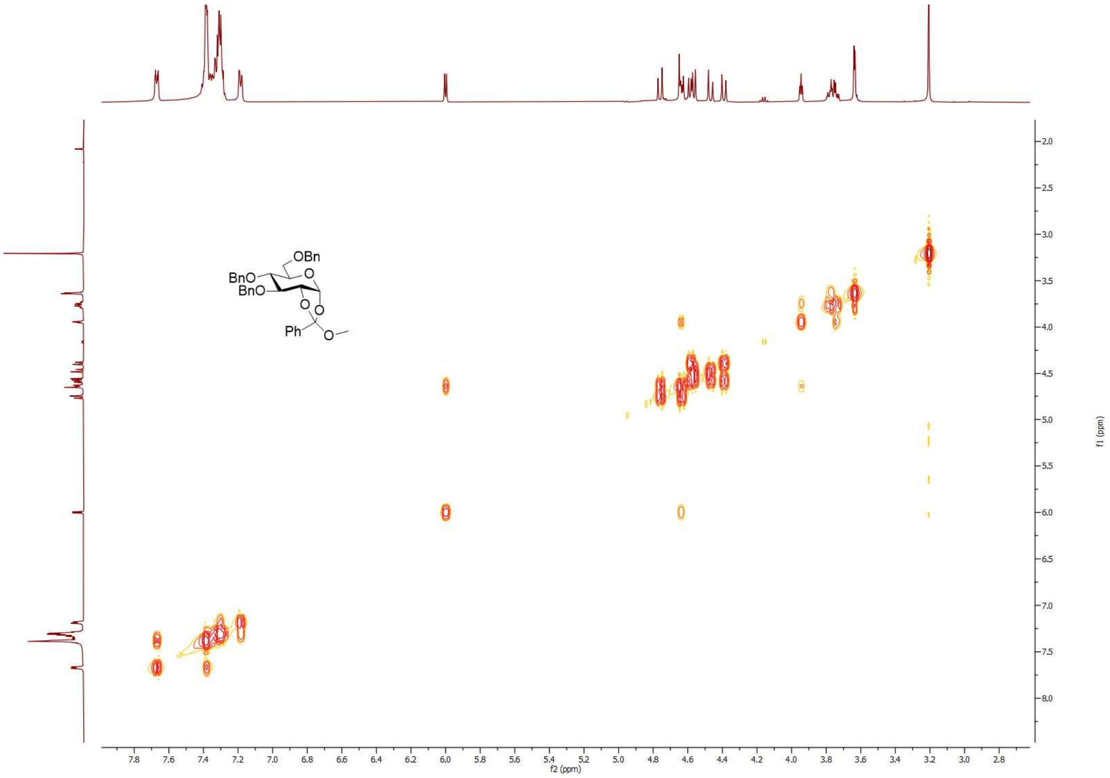

HSQC NMR

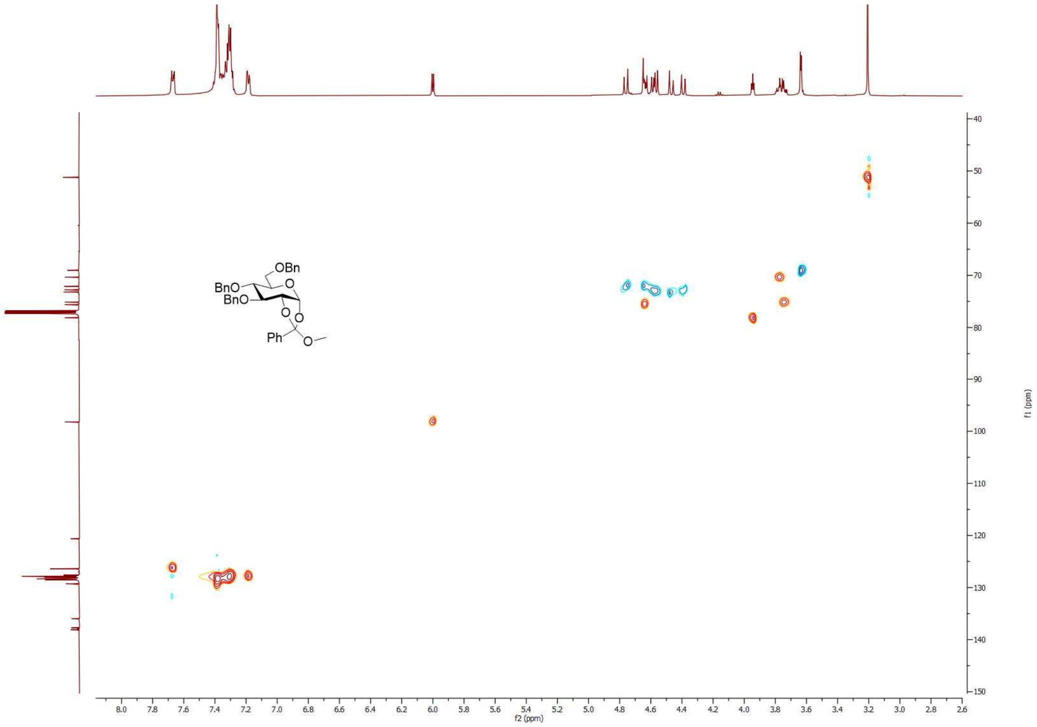

^{1}H NMR (500 MHz, $CDCl_3$)

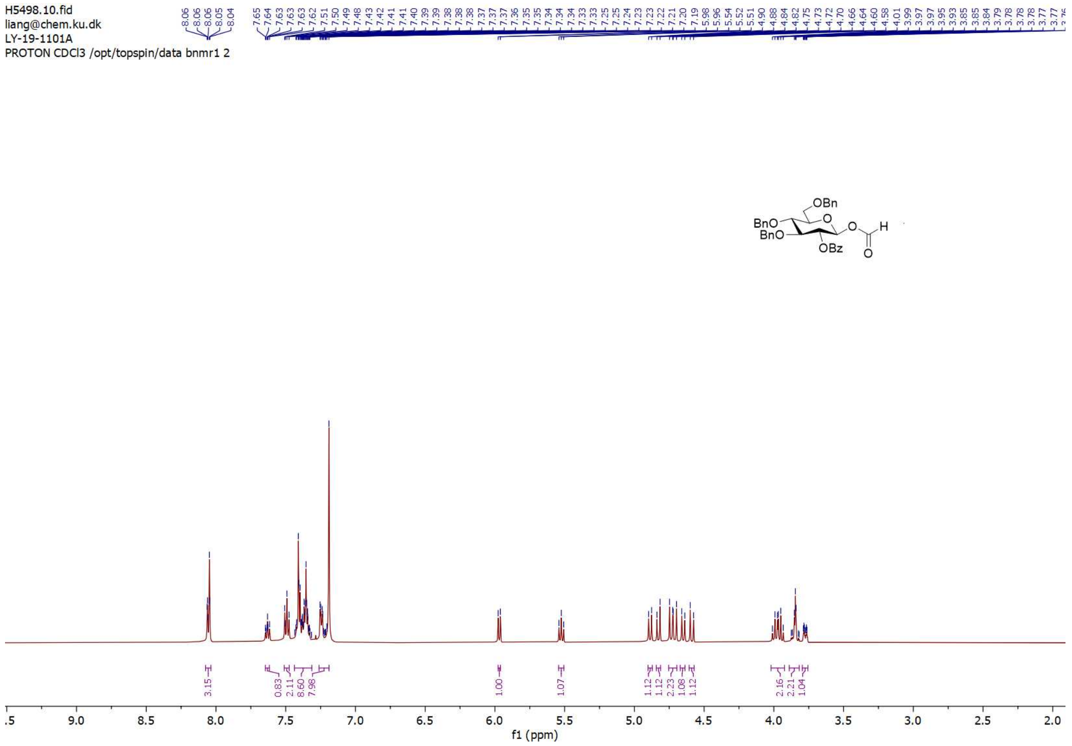

^{13}C NMR (126 MHz, $CDCl_3$)

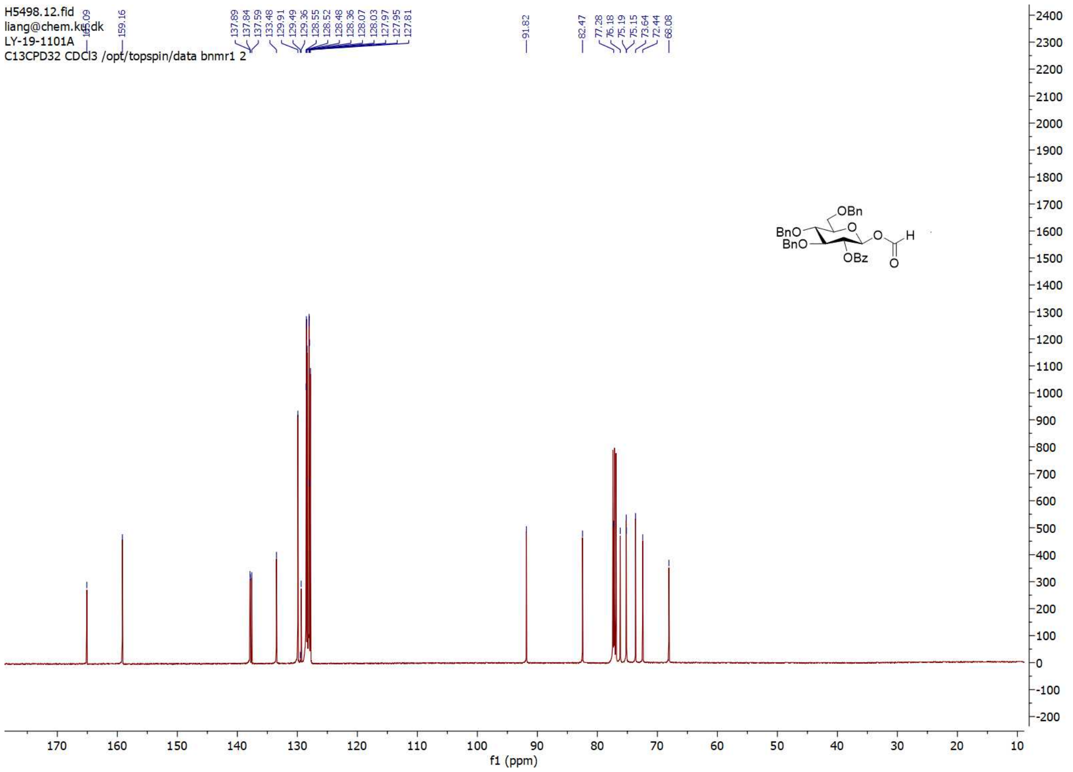
H5498.12.fid
liang@chem.ku.dk
LY-19-1101A
C13CPD32 CDCl3 /opt/topspin/data bnmr1 2
159.16
137.89
137.84
137.59
133.48
129.91
129.49
129.36
128.55
128.52
128.48
128.36
128.07
128.03
127.97
127.95
127.81
91.82
82.47
77.28
76.18
75.19
75.15
73.64
72.44
68.08
f1 (ppm)
BnO
BnO
OBn
OBz
O
H

H-H COSY NMR

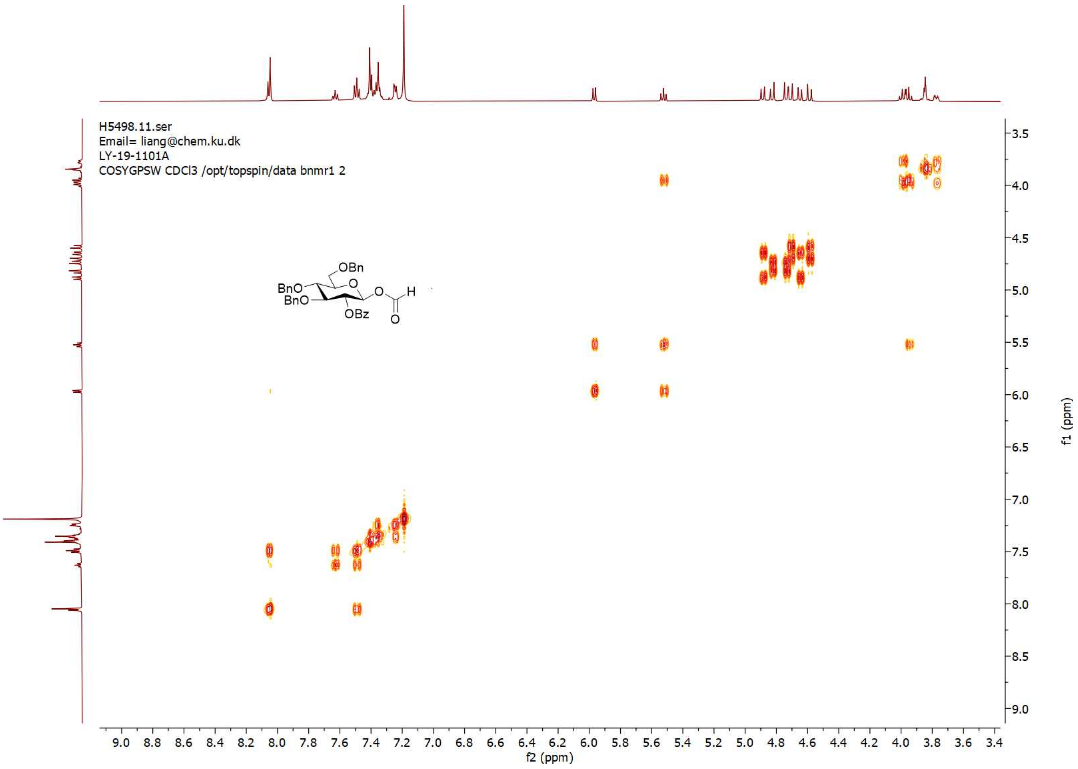
H5498.11.ser
Email= liang@chem.ku.dk
LY-19-1101A
COSYGPSW CDCl3 /opt/topspin/data bnmr1 2
BnO
BnO
OBn
OBz
O
H
f2 (ppm)
f1 (ppm)

HSQC NMR

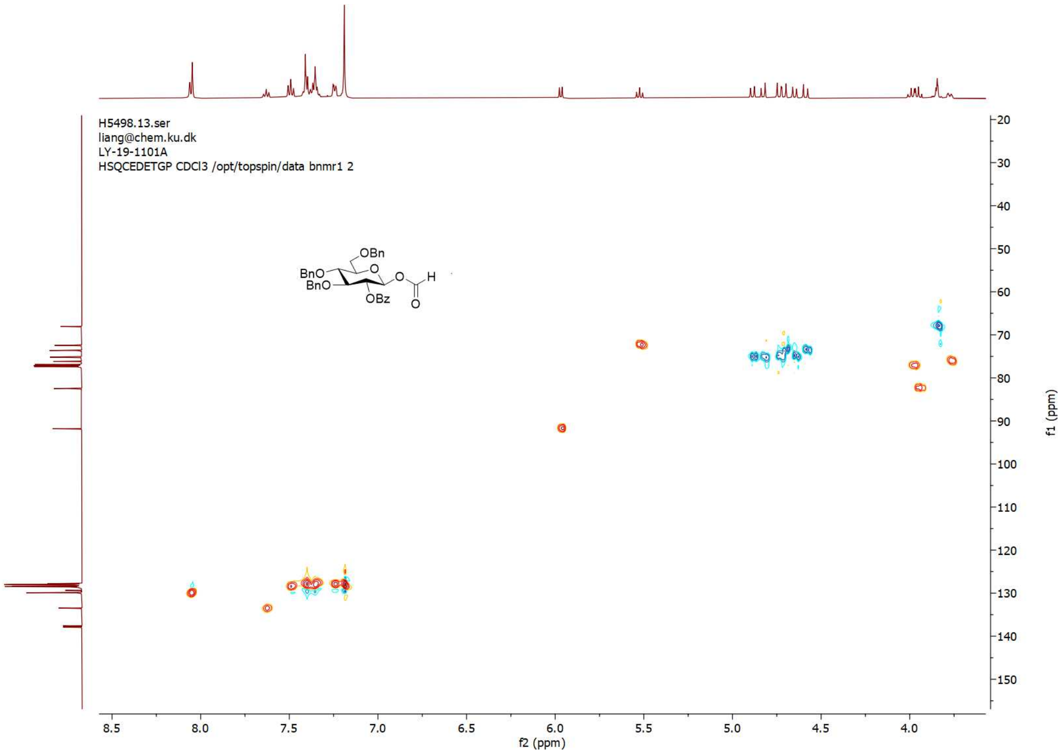

^{1}H NMR (500 MHz, $CDCl_3$)

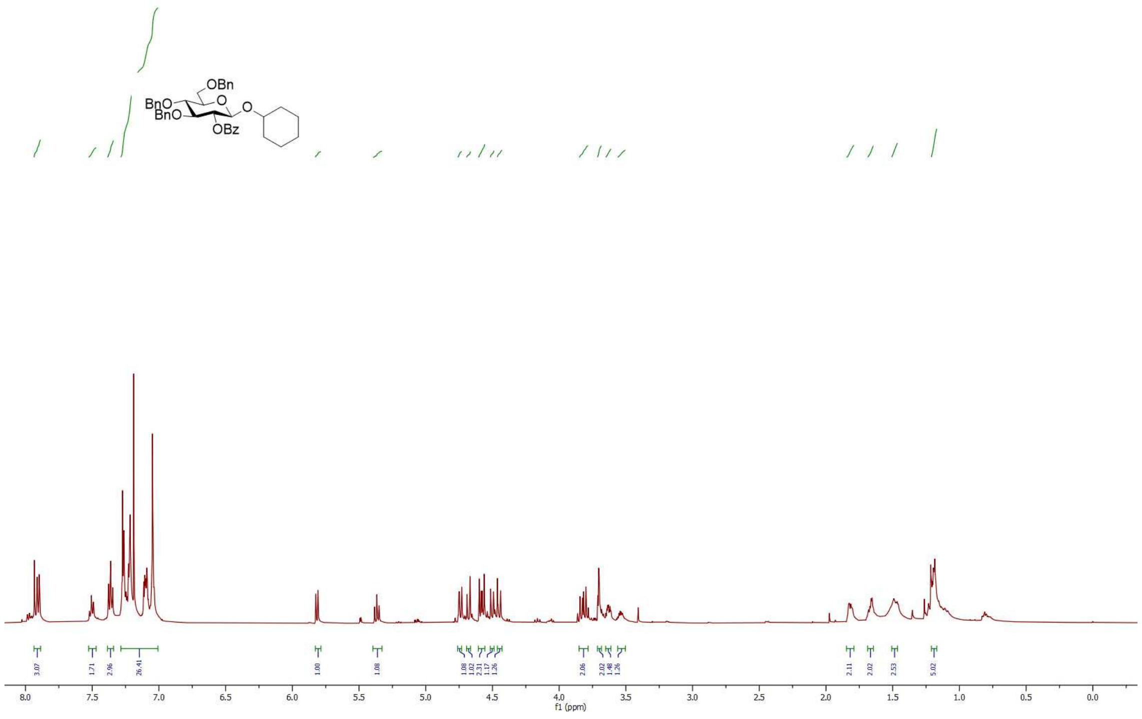

^{13}C NMR (126 MHz, $CDCl_3$)

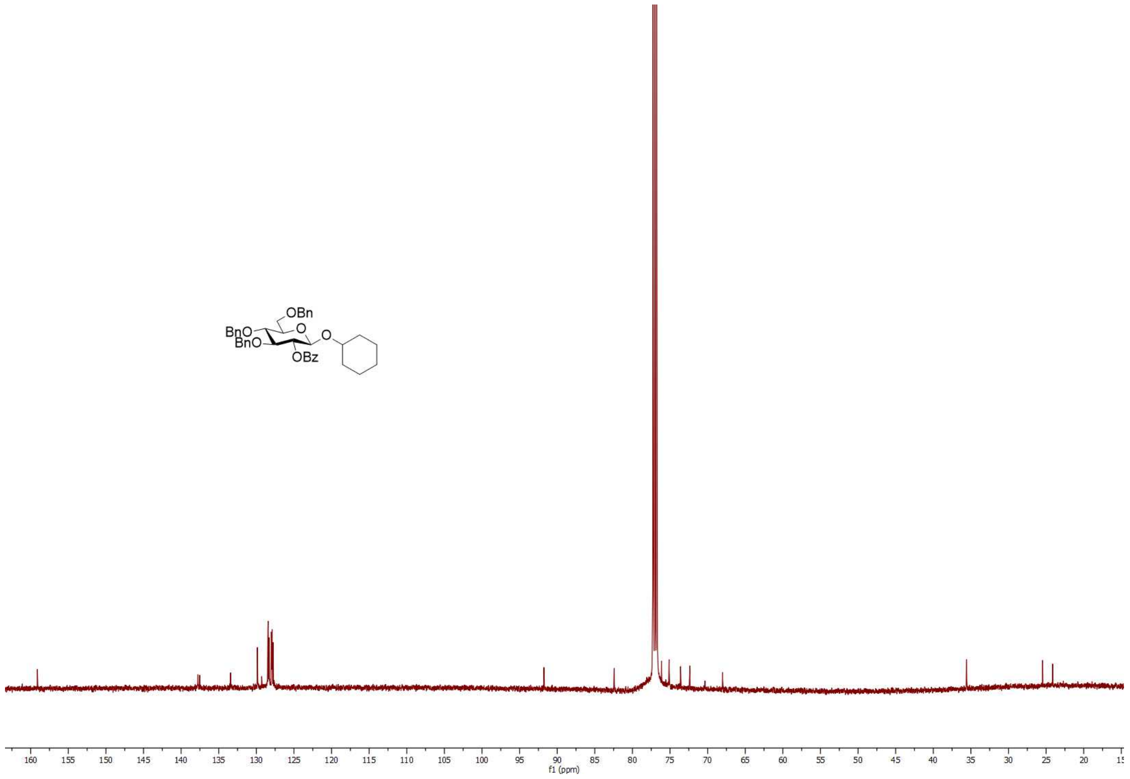

H-H COSY NMR

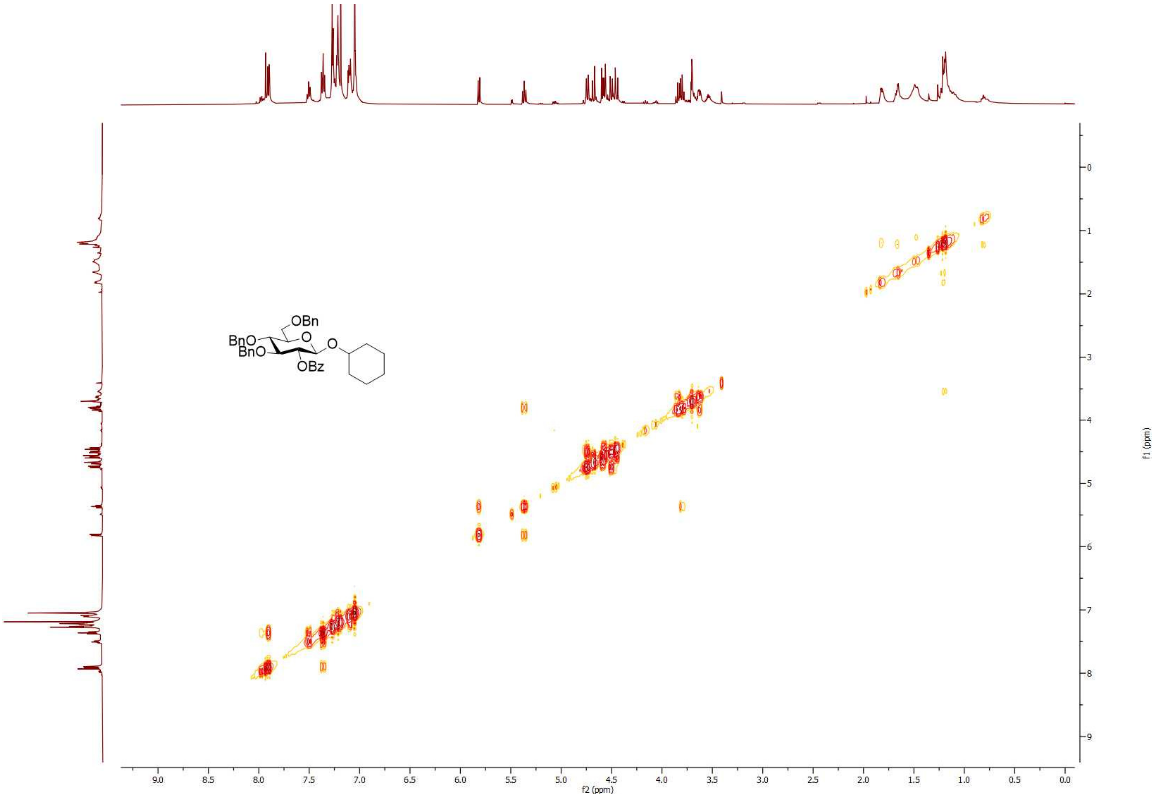

HSQC NMR

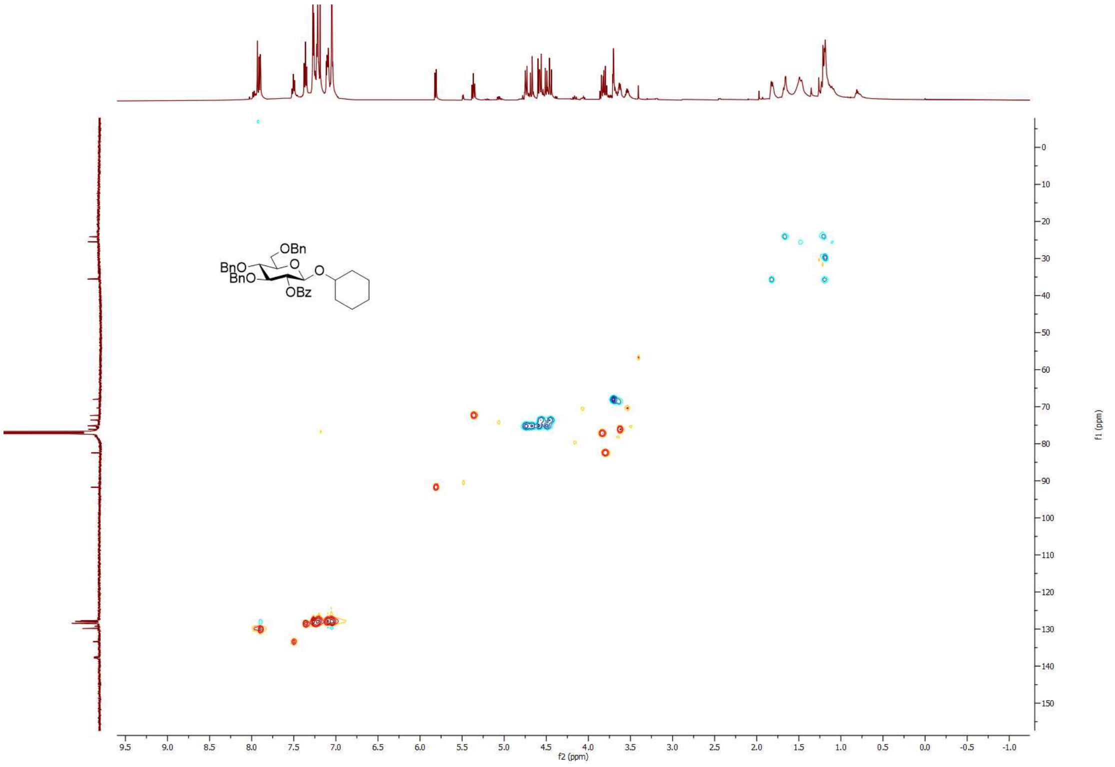

^{1}H NMR (500 MHz, $CDCl_3$)

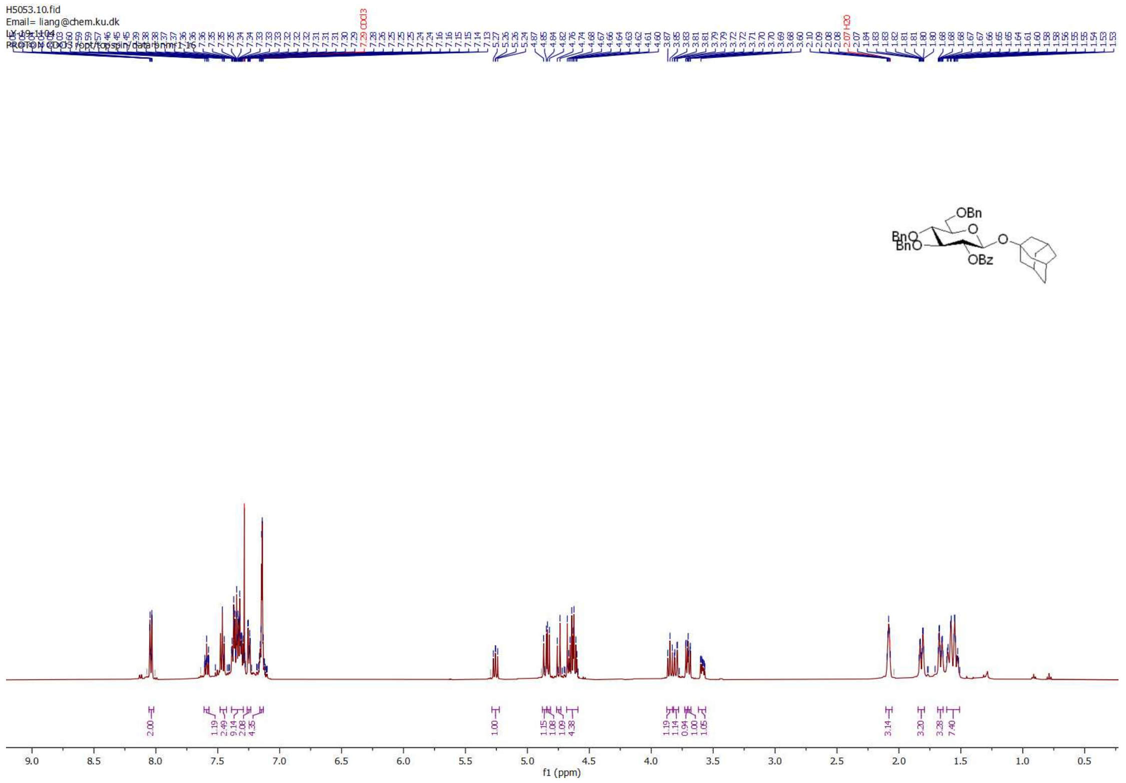

^{13}C NMR (126 MHz, $CDCl_3$)

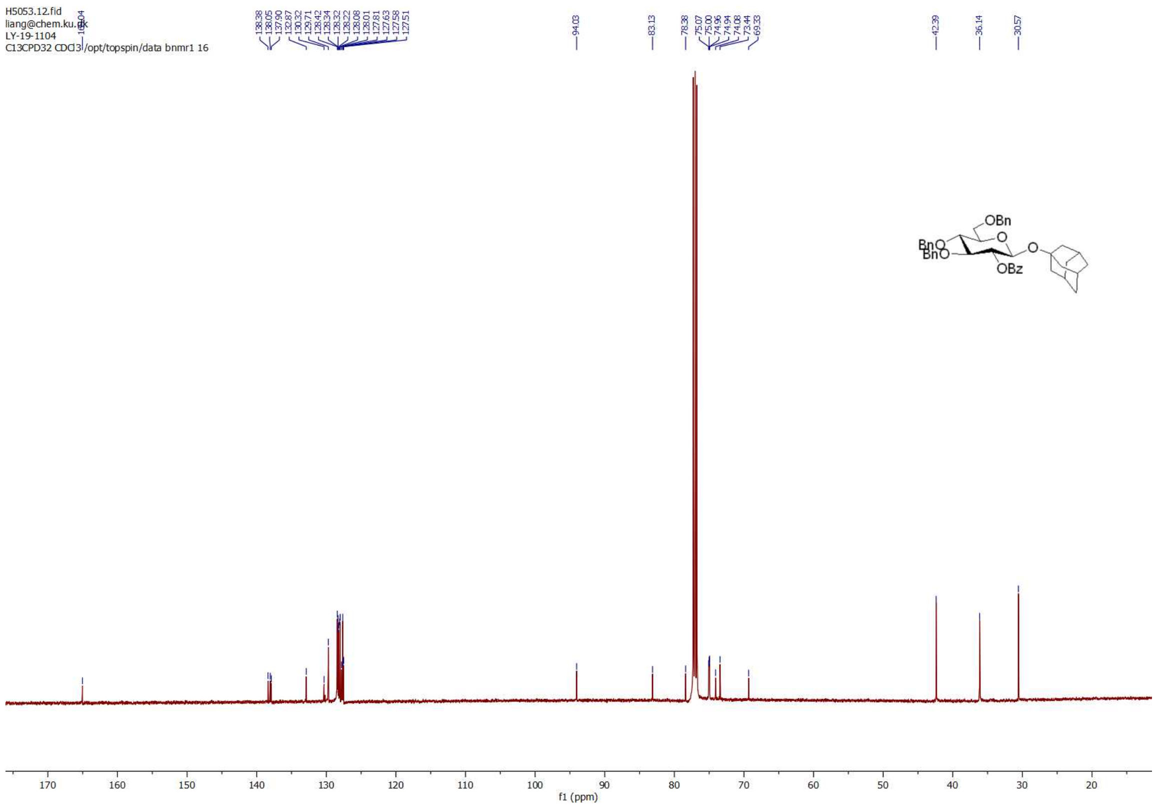

H-H COSY NMR

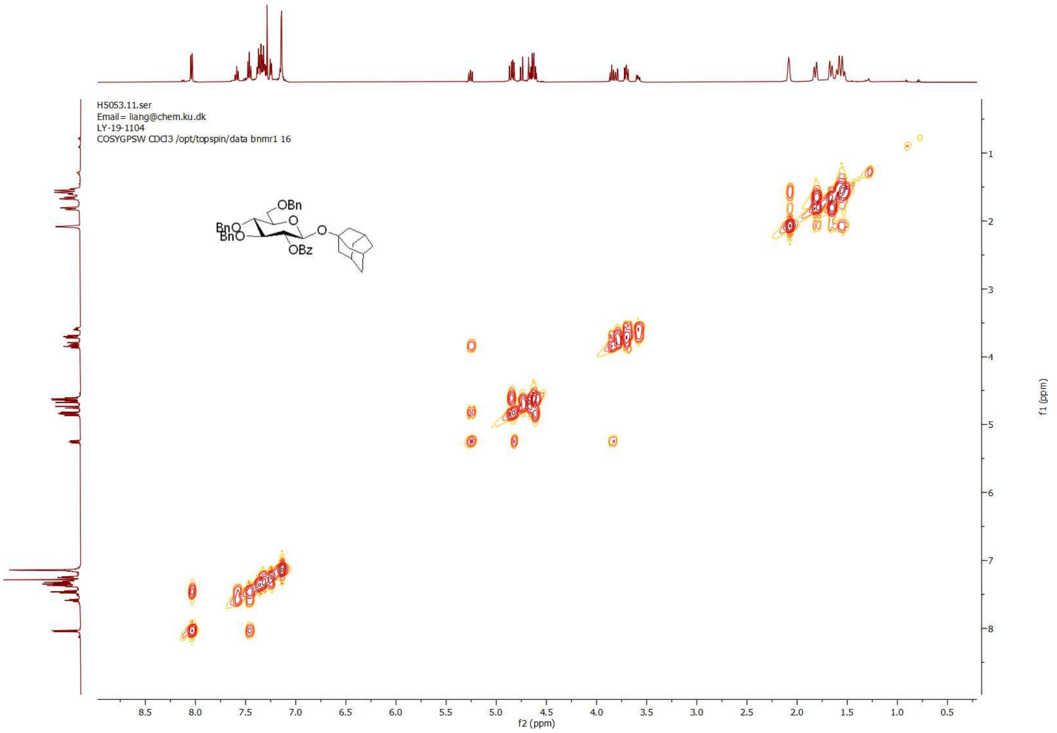

HSQC NMR

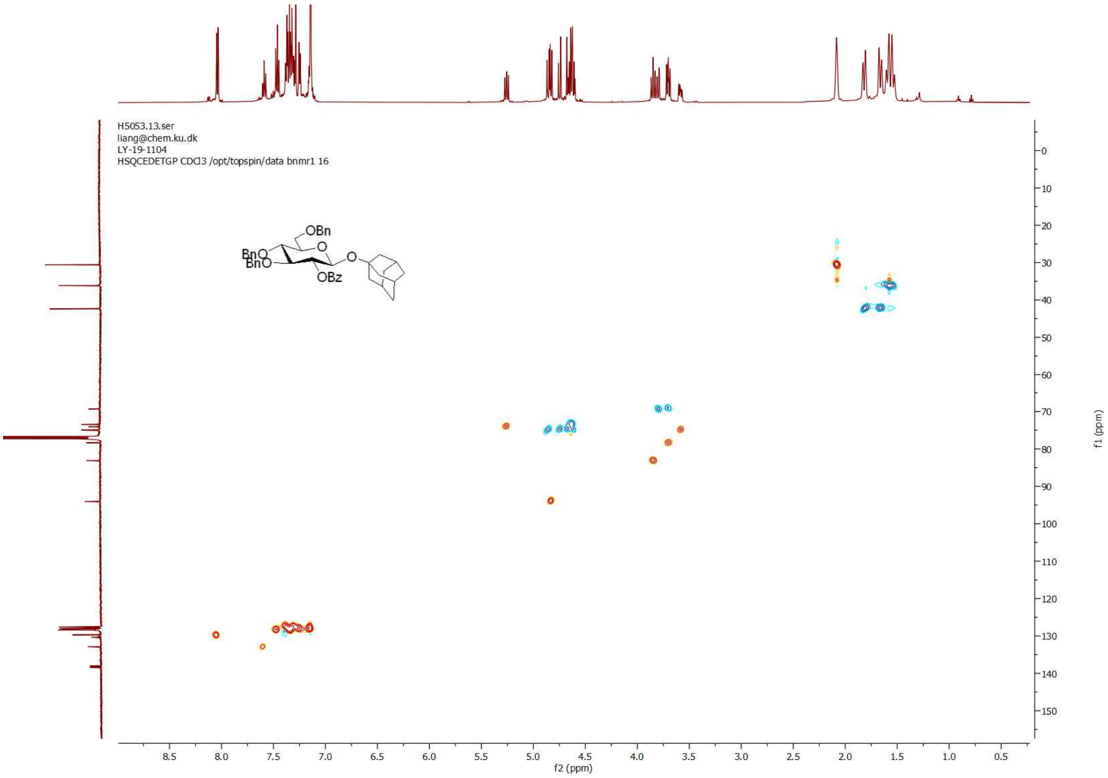

^{1}H NMR (500 MHz, CDCl$_3$)

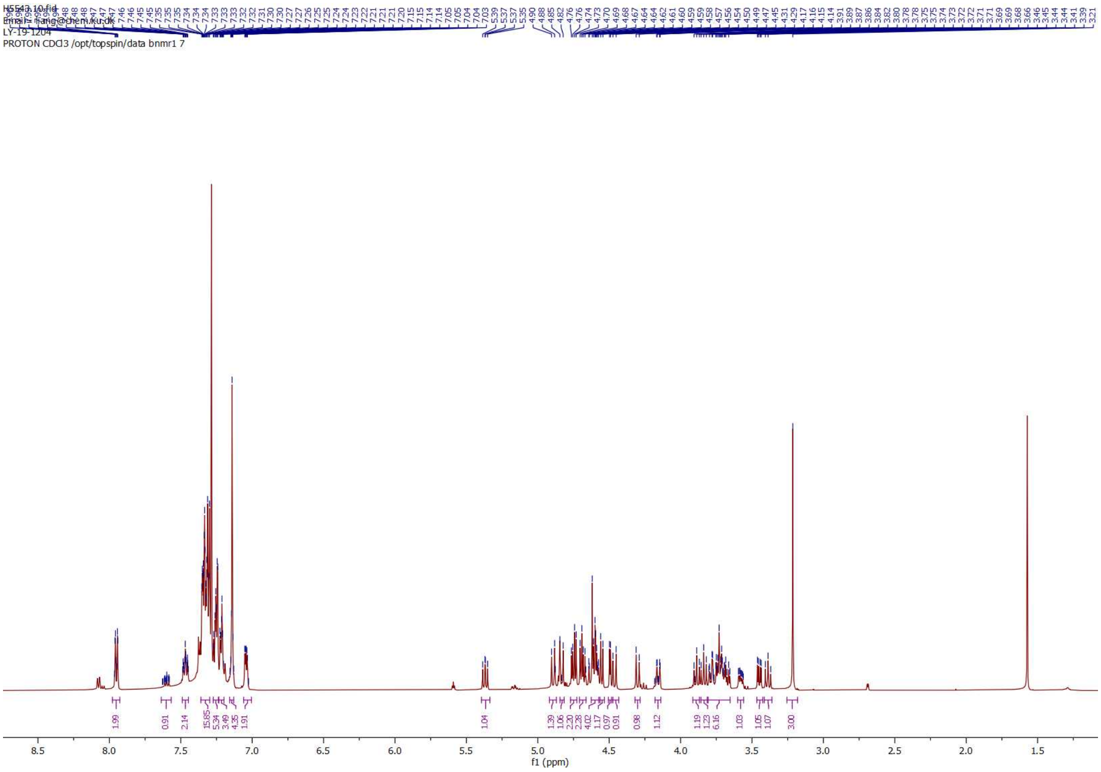

^{13}C NMR (126 MHz, CDCl$_3$)

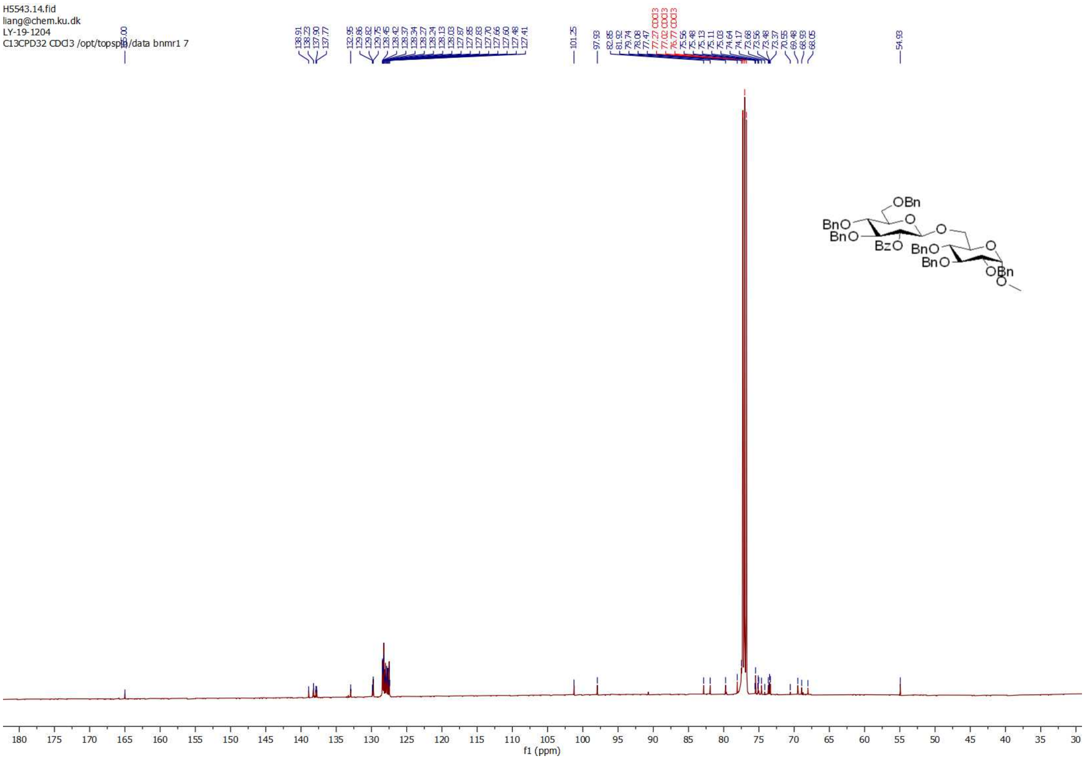

H-H COSY NMR

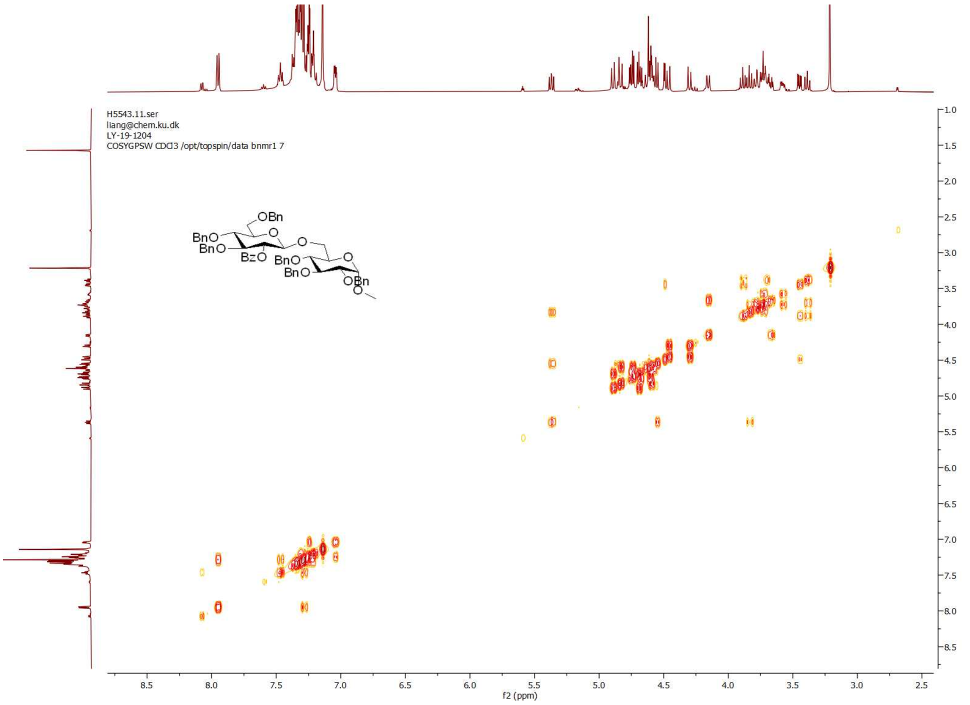

HSQC NMR

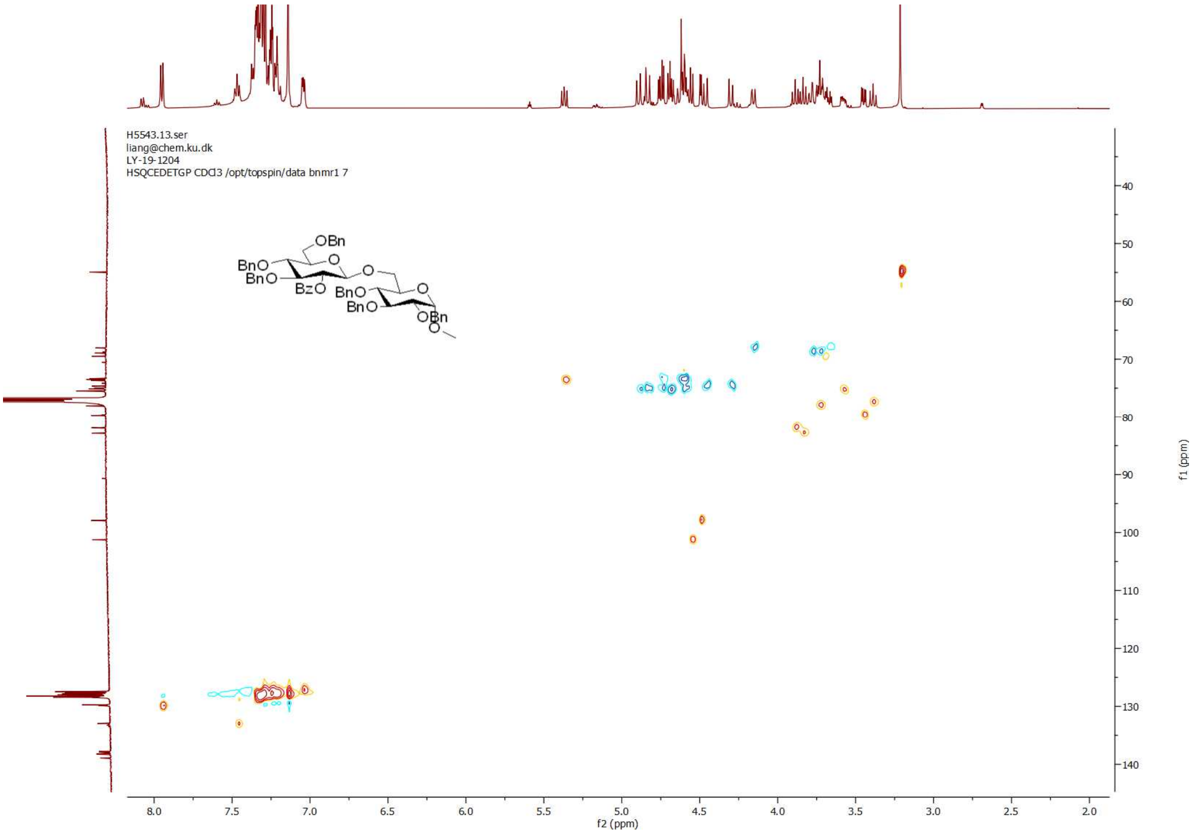

^{1}H NMR (500 MHz, $CDCl_3$)

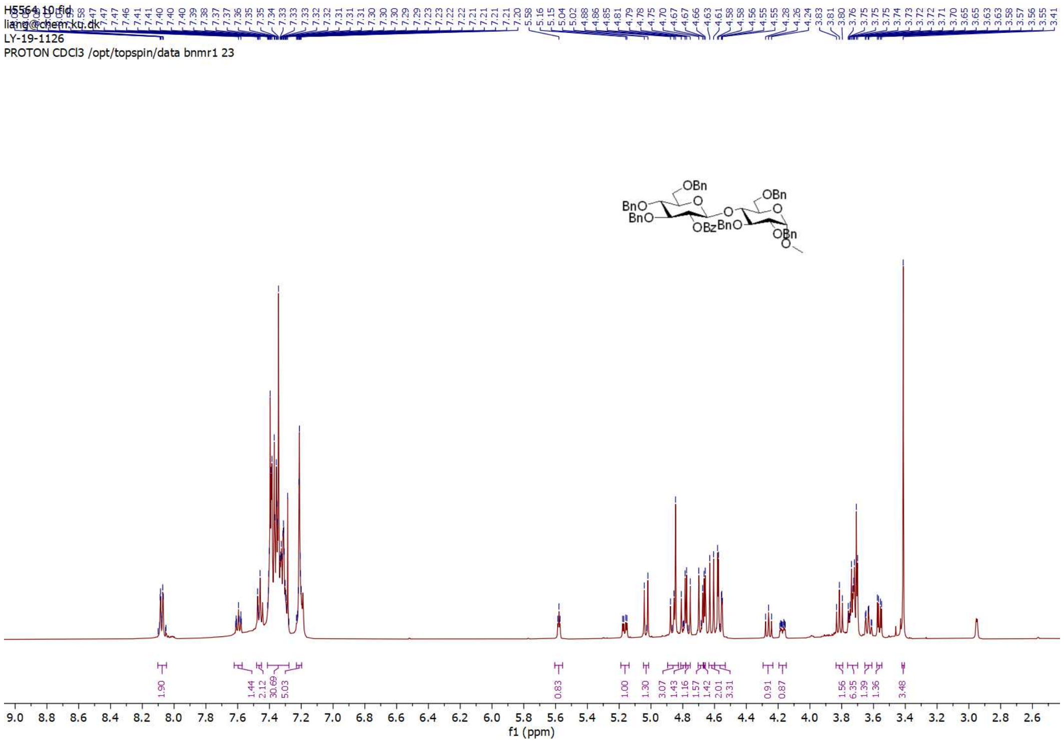

^{13}C NMR (126 MHz, $CDCl_3$)

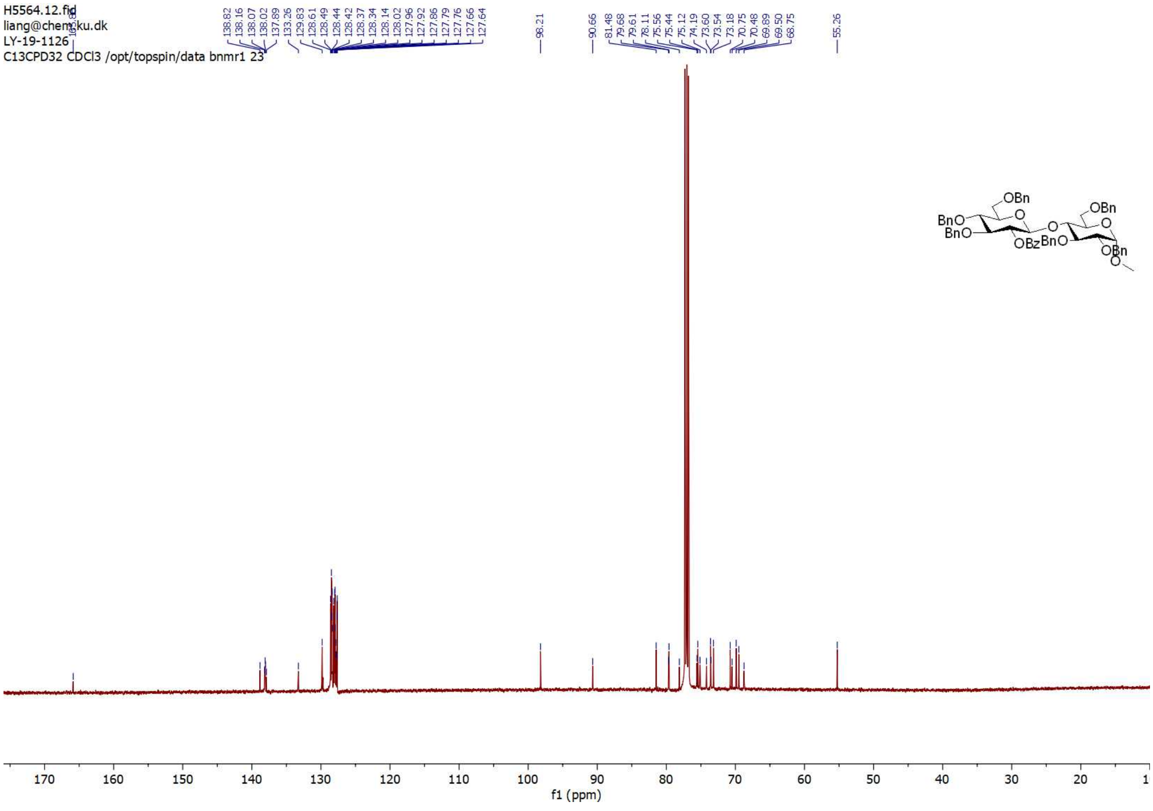

H-H COSY NMR

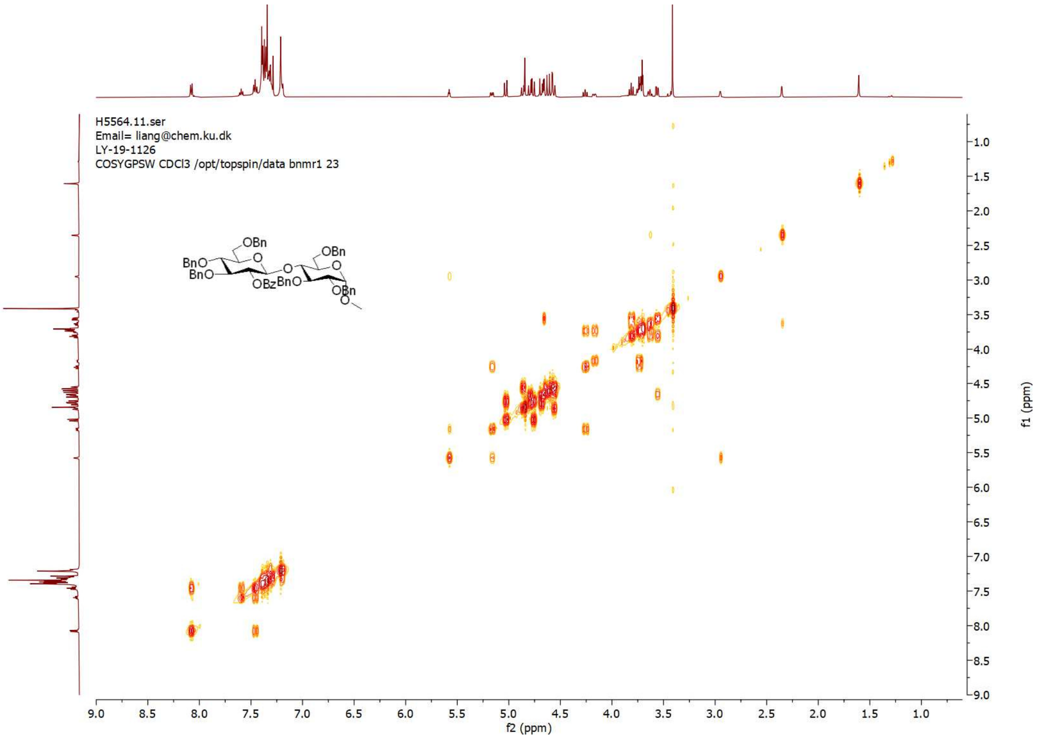

HSQC NMR

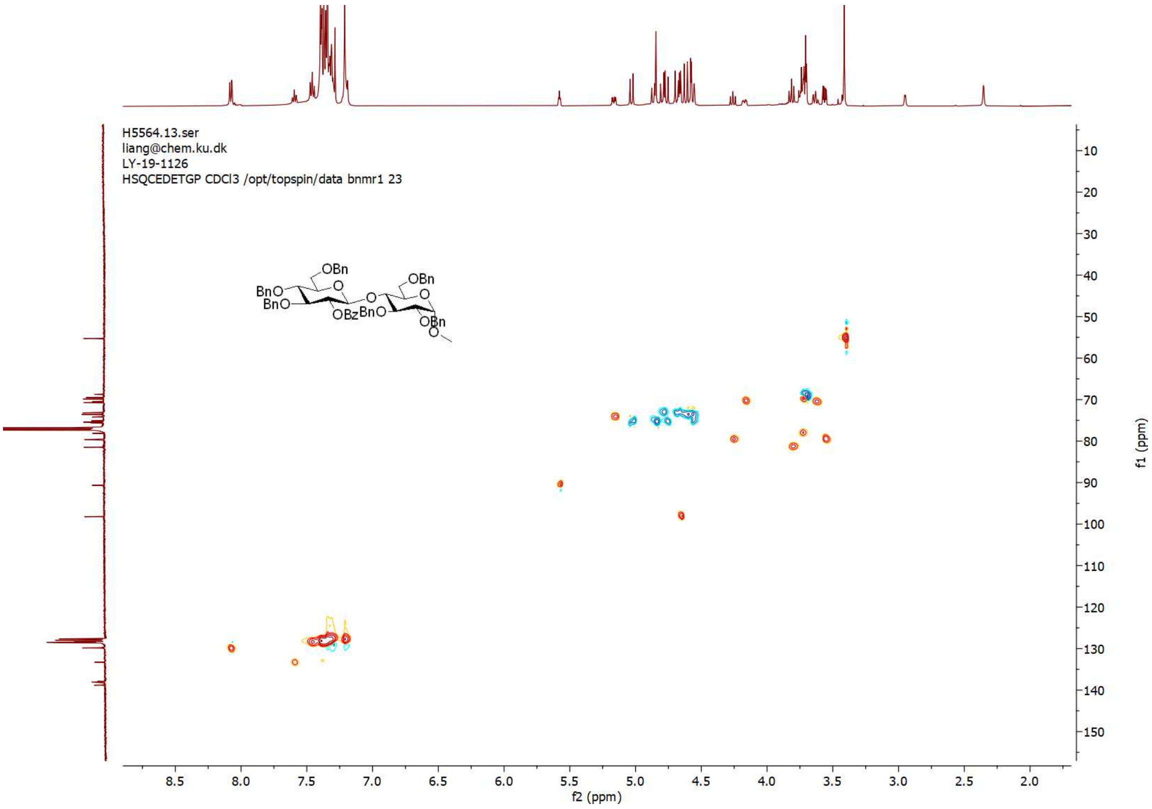

^{1}H NMR (500 MHz, $CDCl_3$)

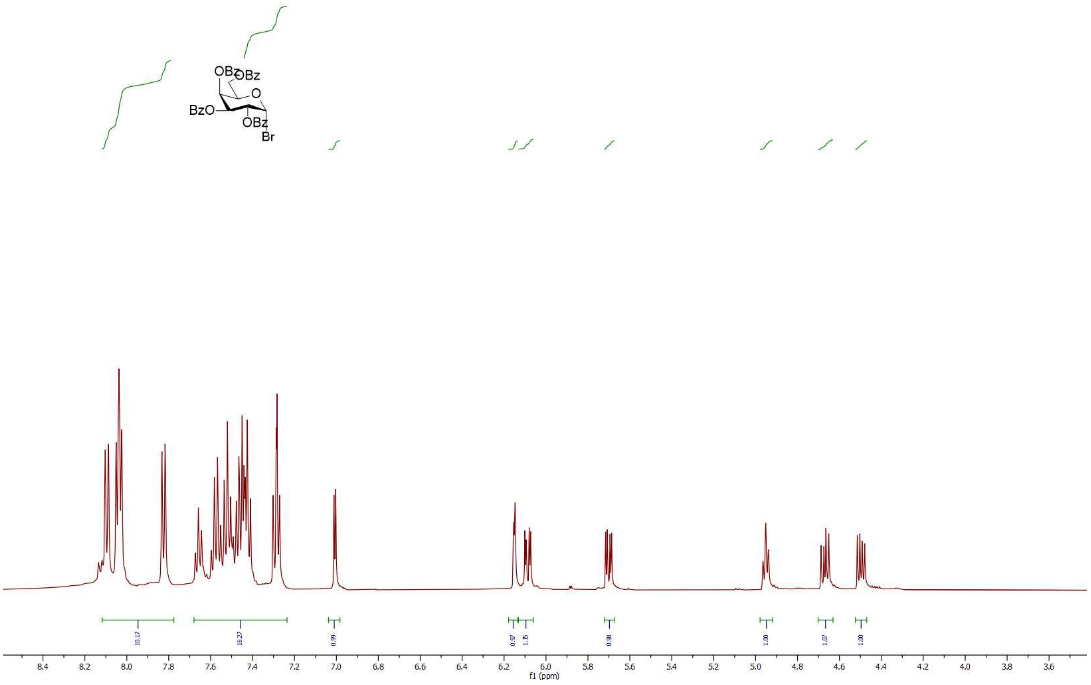

^{13}C NMR (126 MHz, $CDCl_3$)

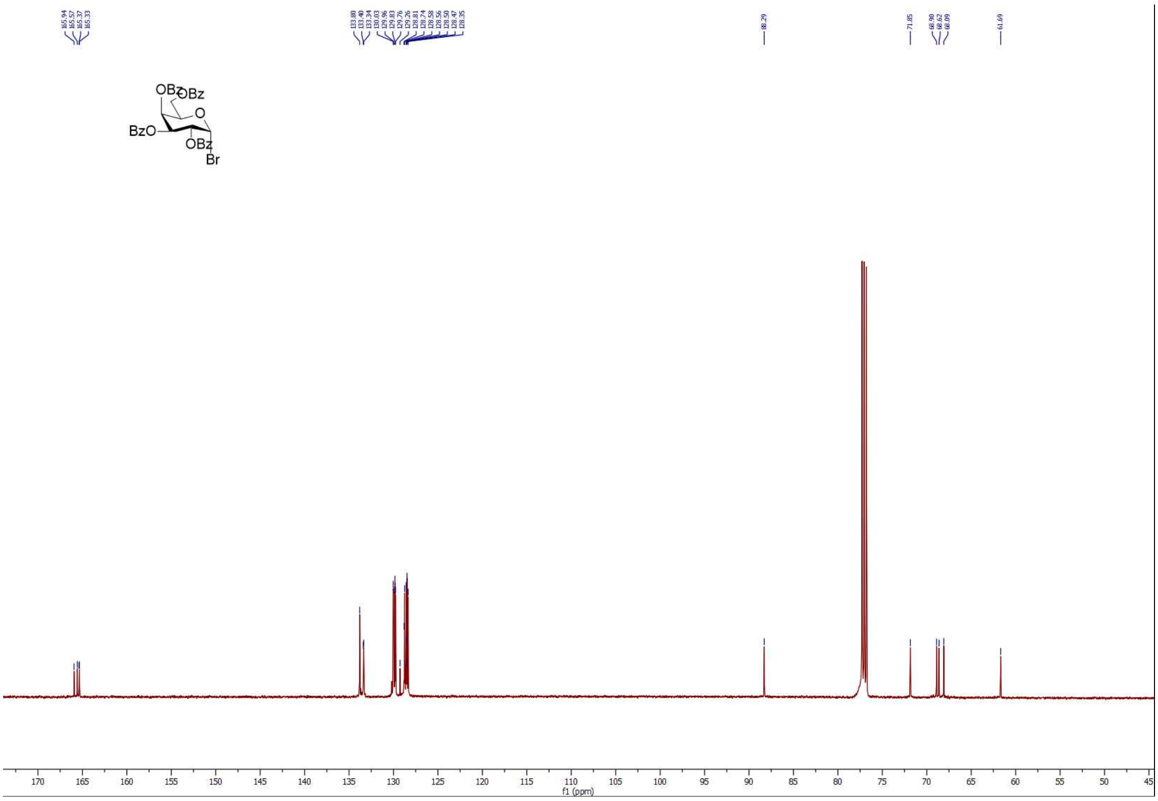

H-H COSY NMR

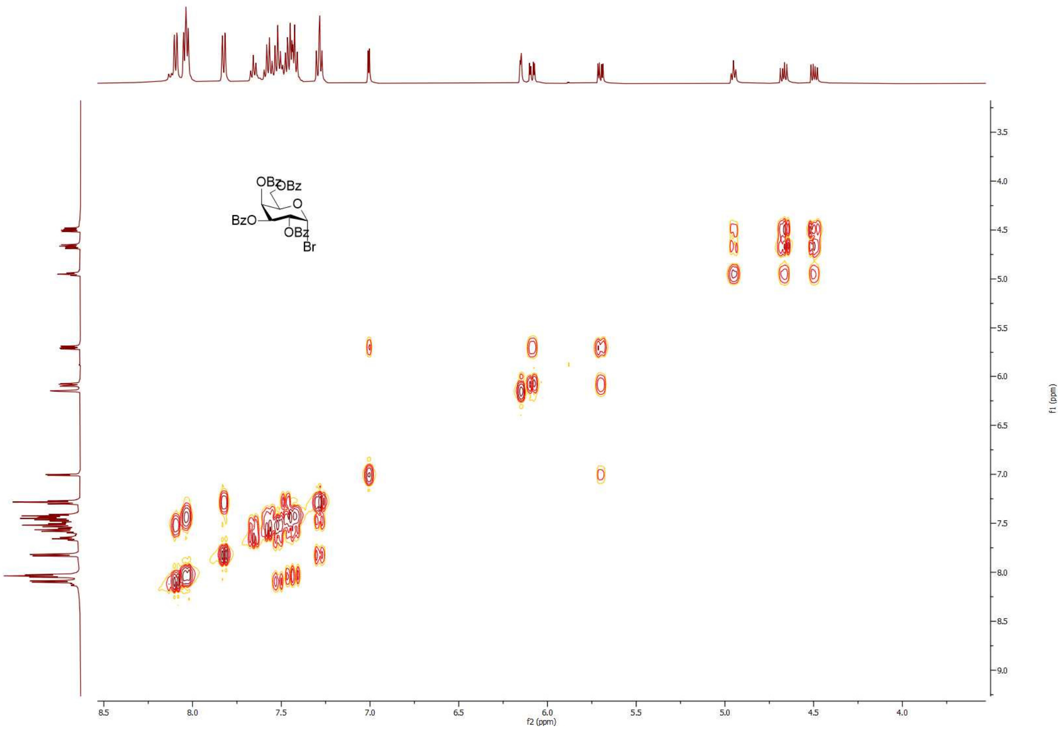

HSQC NMR

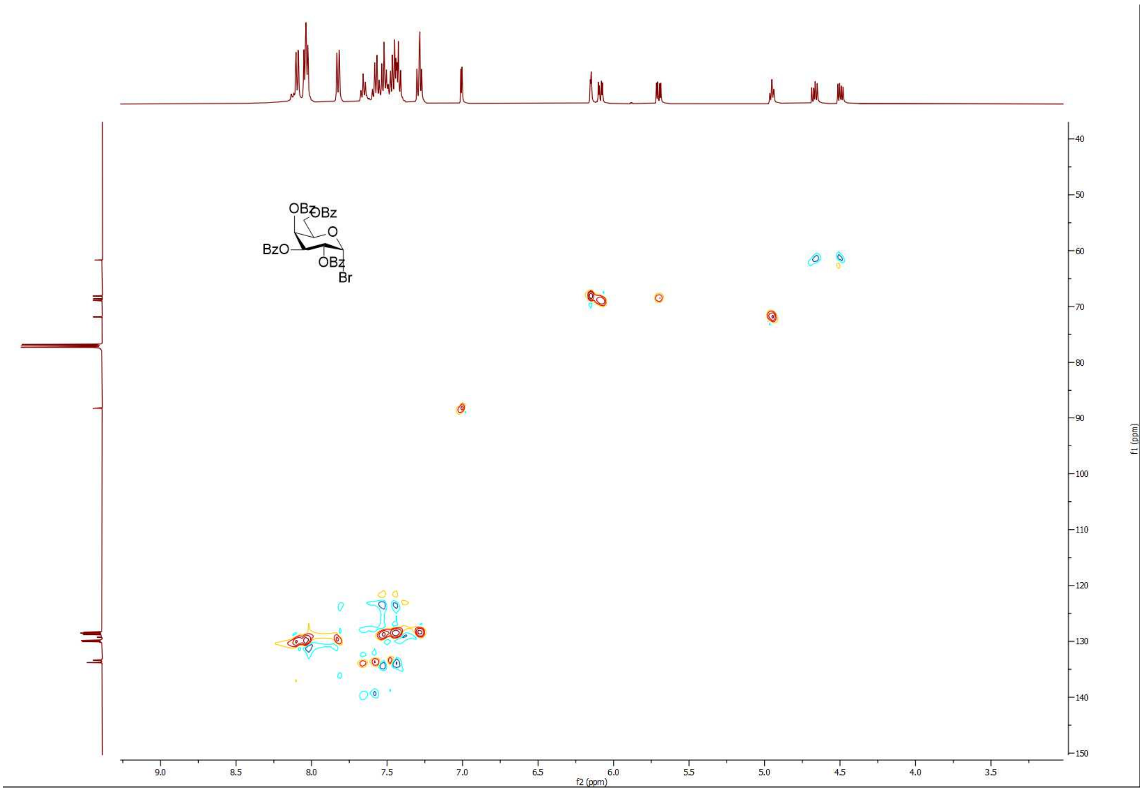

^{1}H NMR (500 MHz, $CDCl_3$)

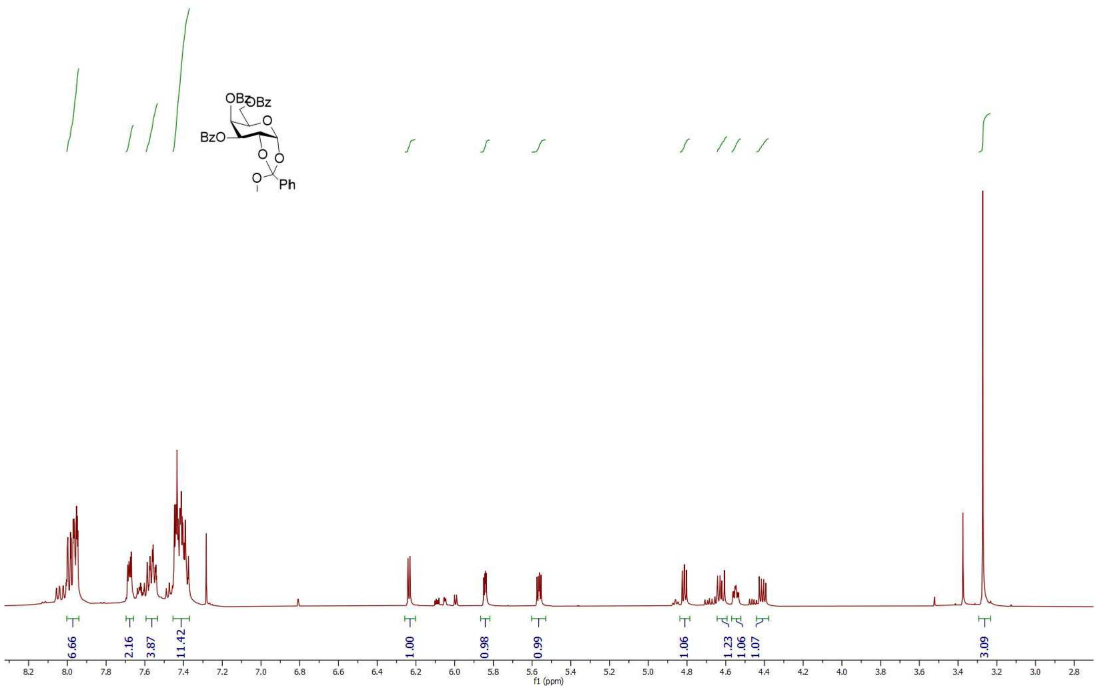

^{13}C NMR (126 MHz, $CDCl_3$)

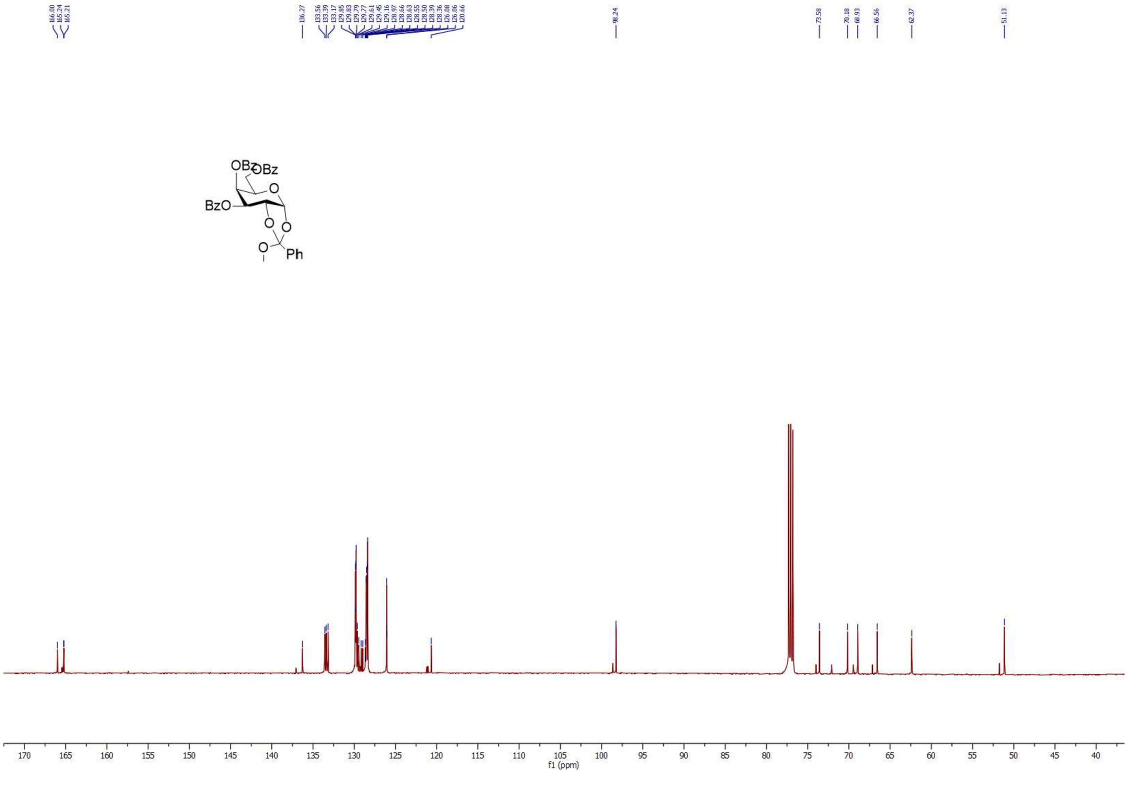

H-H COSY NMR

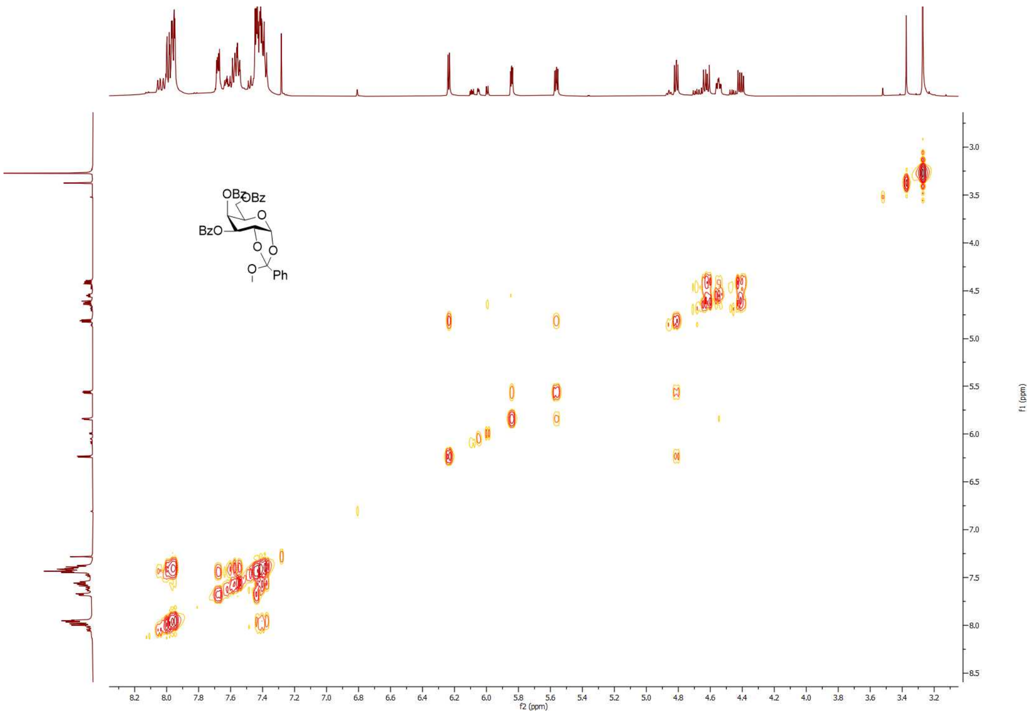

HSQC NMR

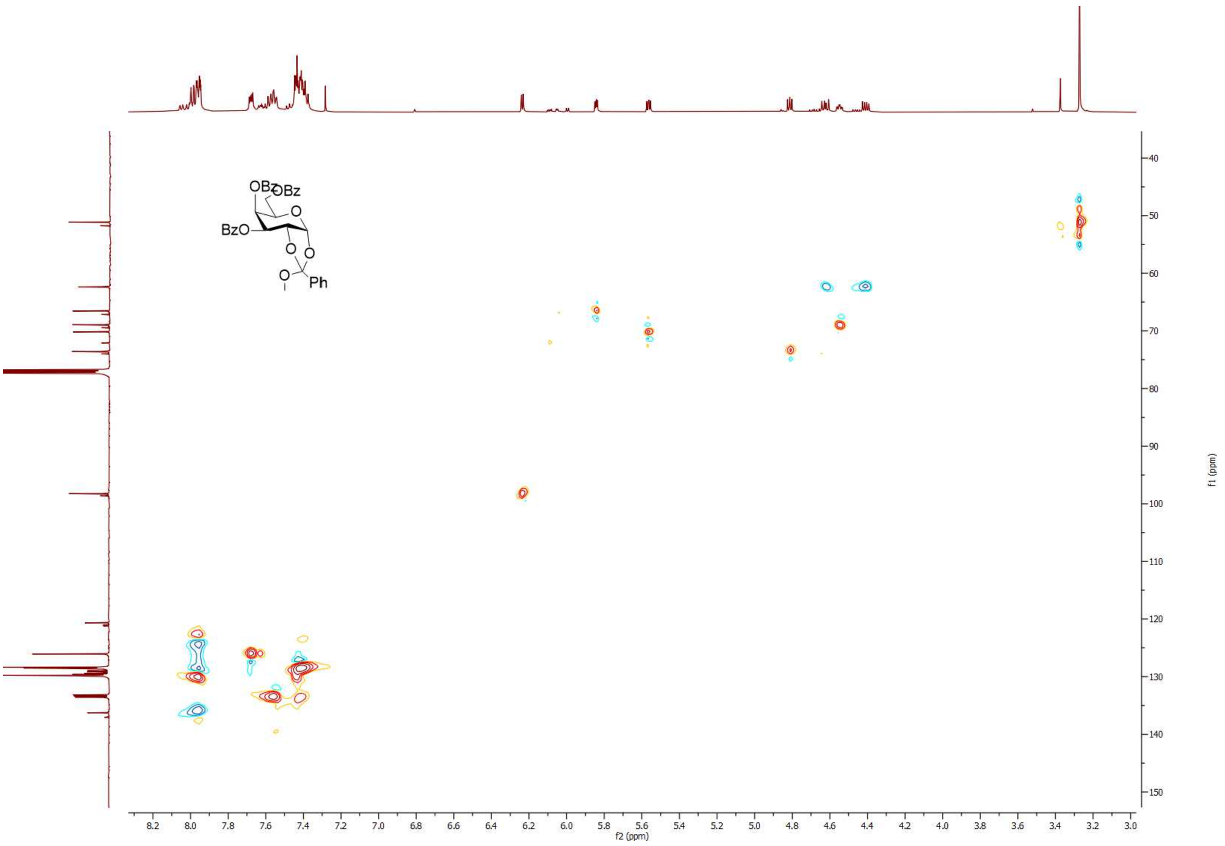

^{1}H NMR (500 MHz, $CDCl_3$)

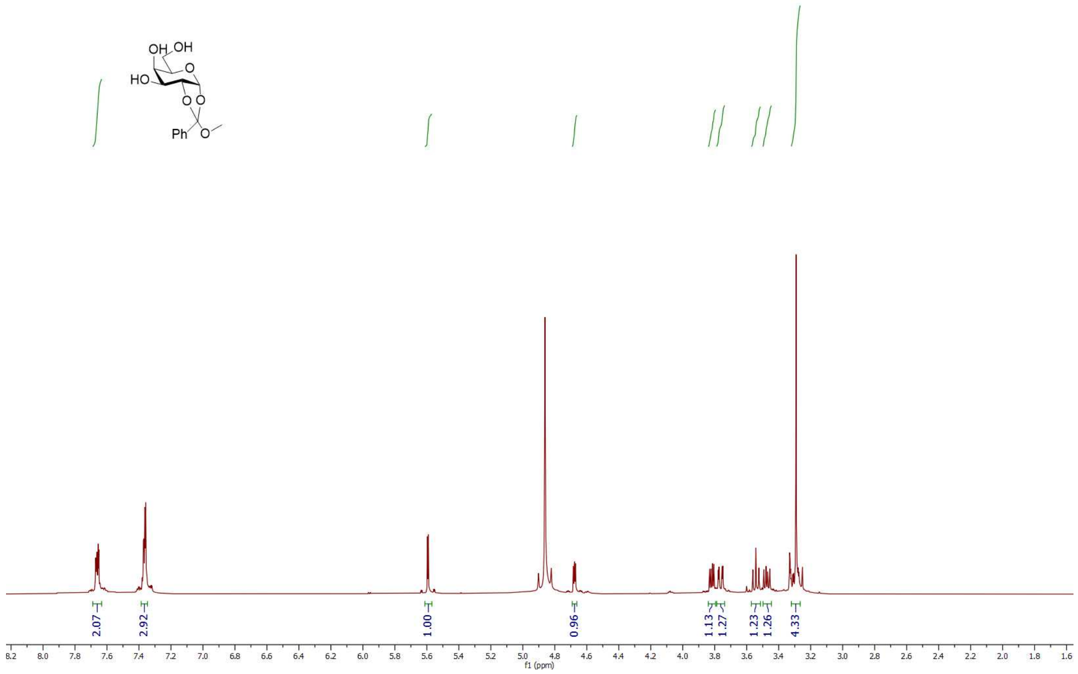

^{13}C NMR (126 MHz, $CDCl_3$)

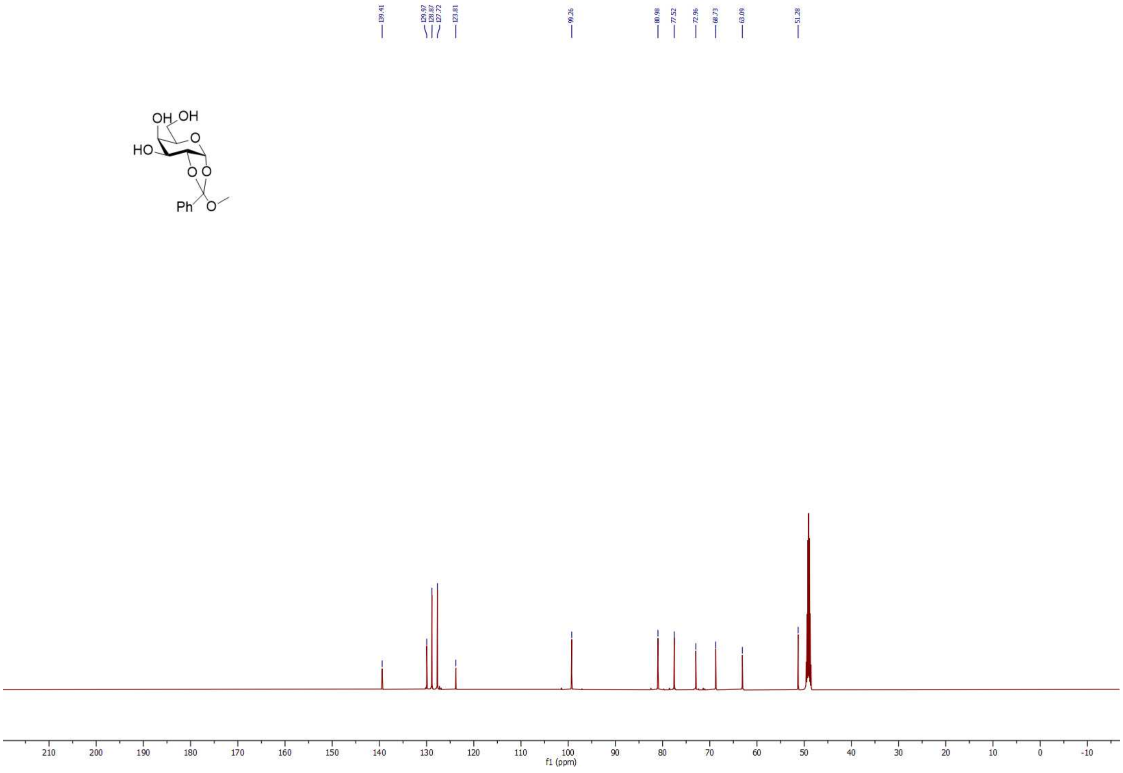

H-H COSY NMR

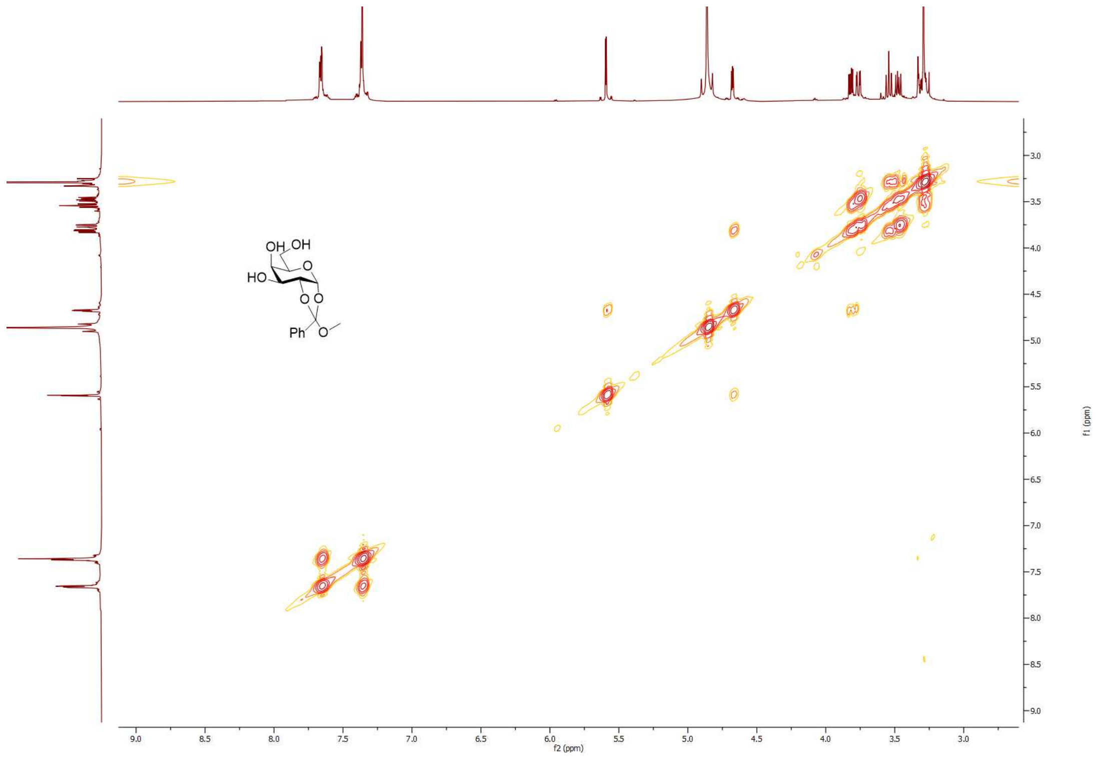

HSQC NMR

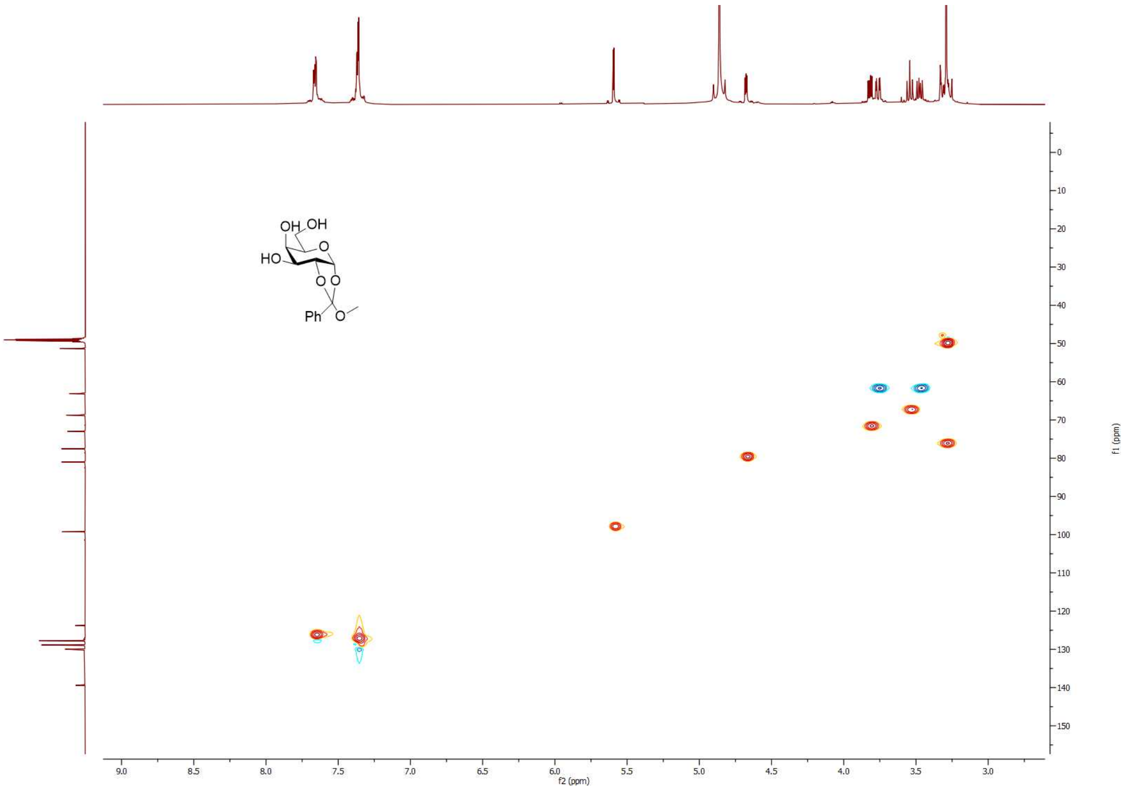

^1H NMR (500 MHz, $CDCl_3$)

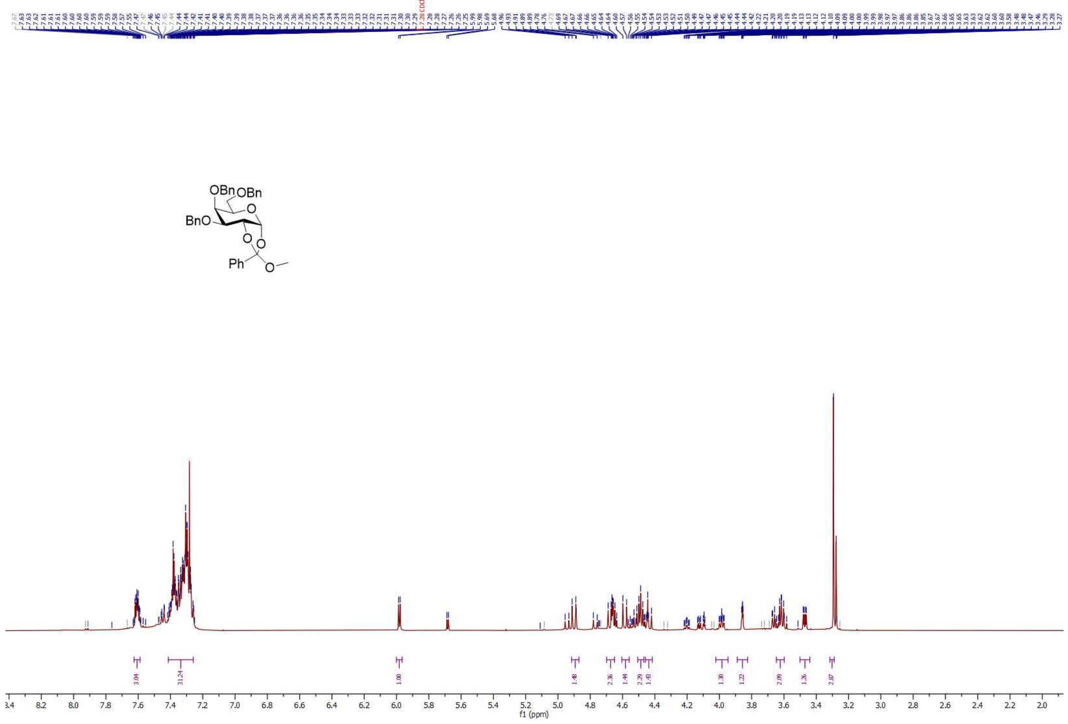

^{13}C NMR (126 MHz, $CDCl_3$)

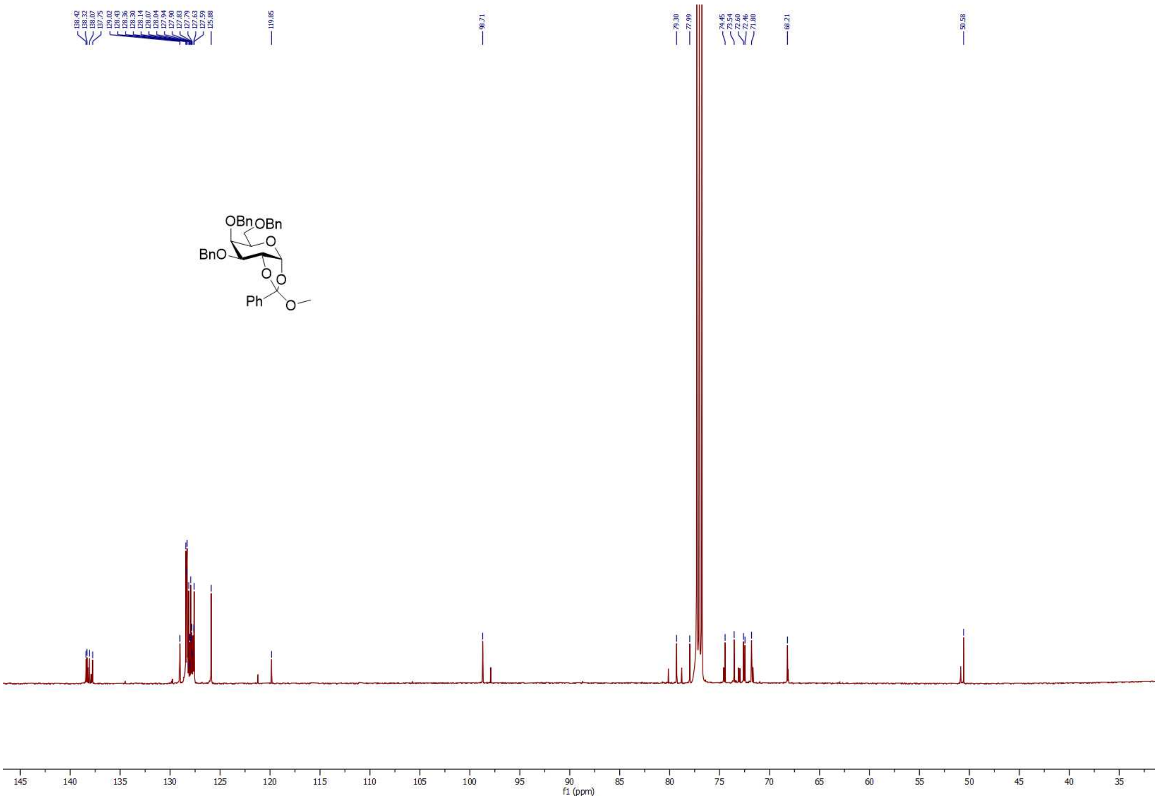

H-H COSY NMR

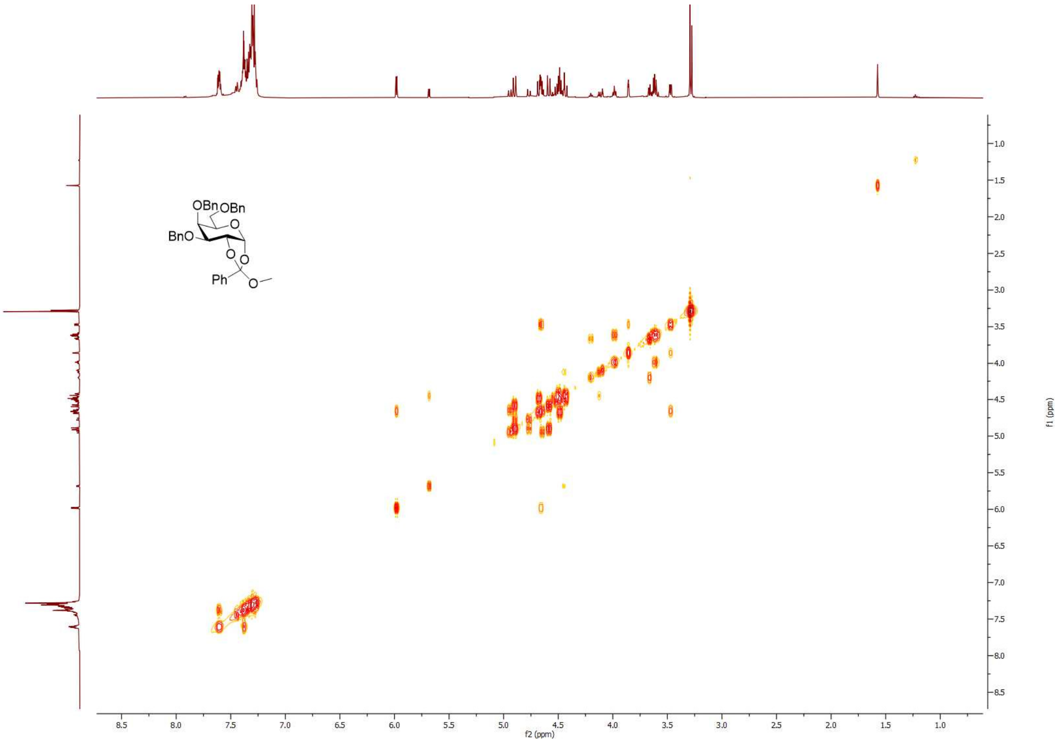

HSQC NMR

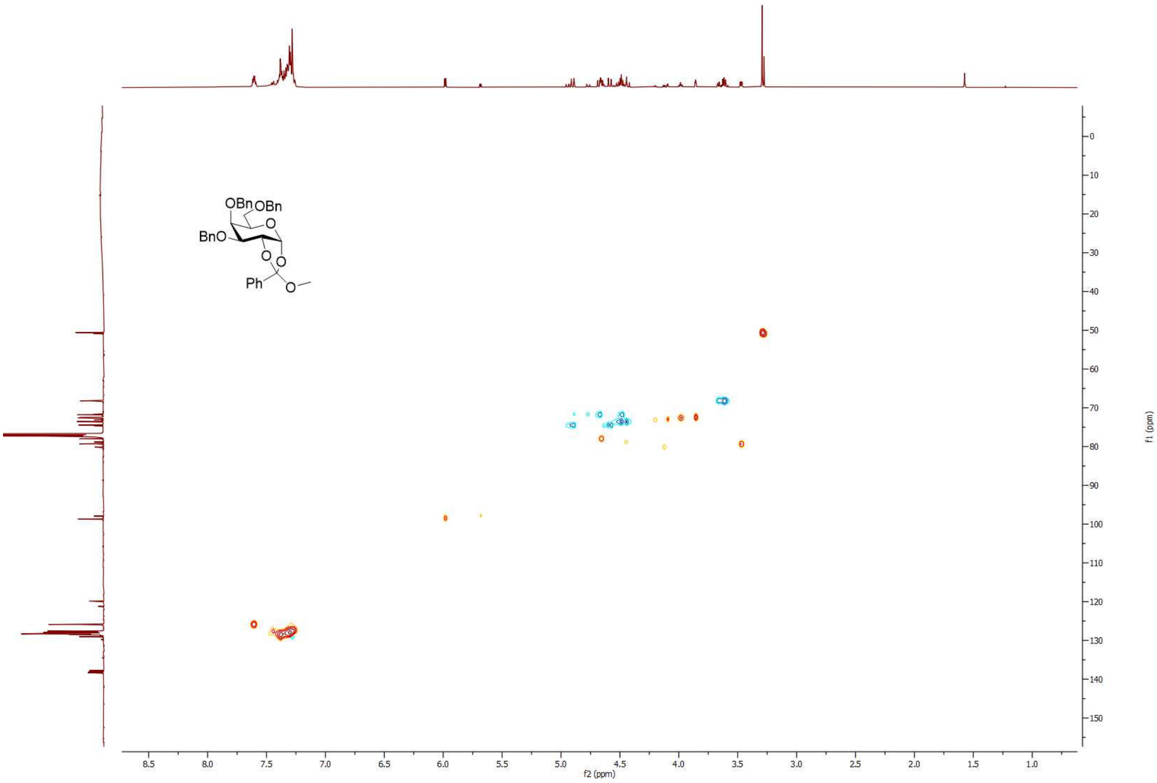

^{1}H NMR (500 MHz, $CDCl_3$)

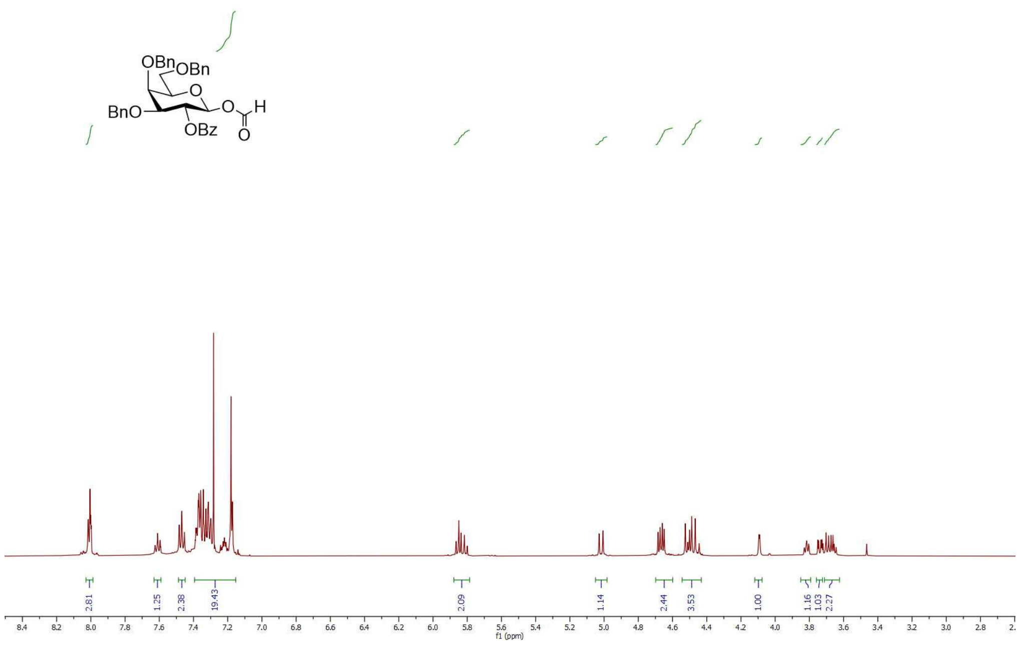

^{13}C NMR (126 MHz, $CDCl_3$)

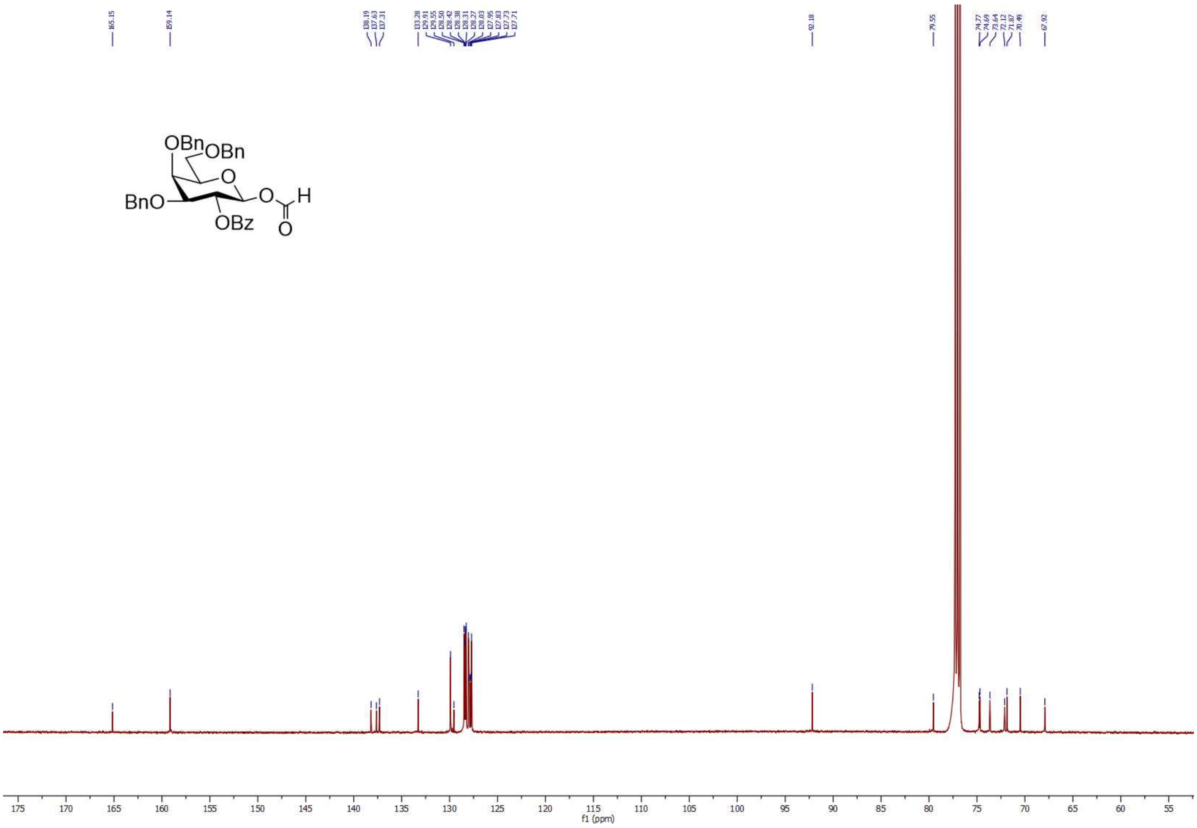

H-H COSY NMR

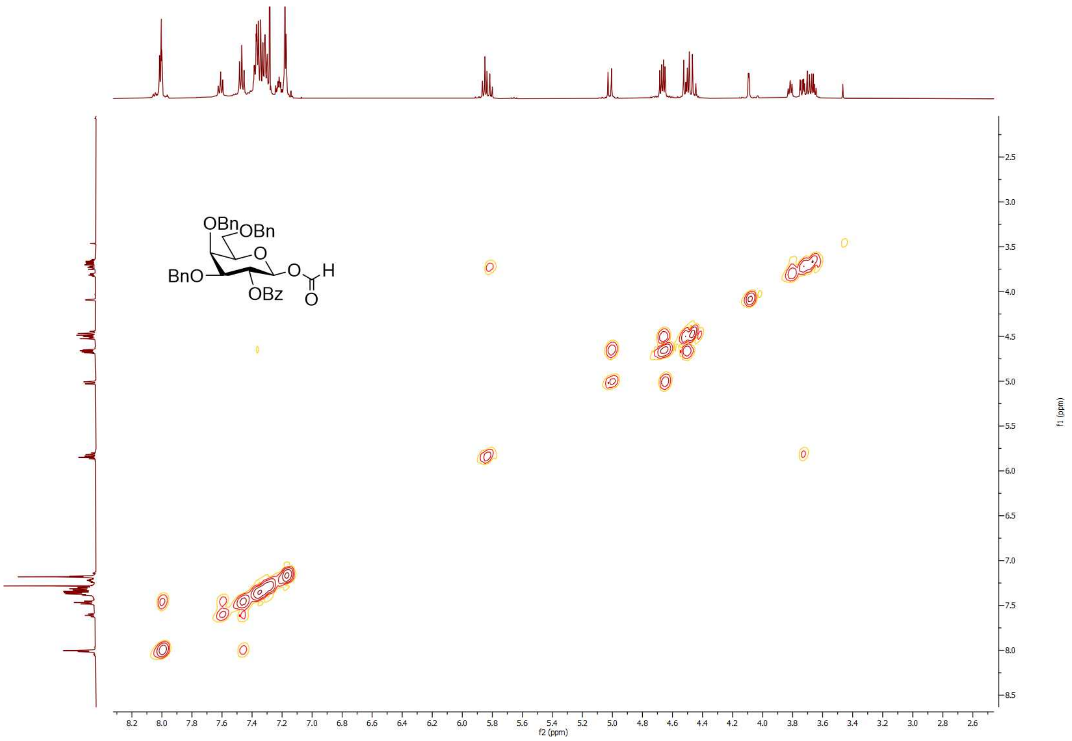

HSQC NMR

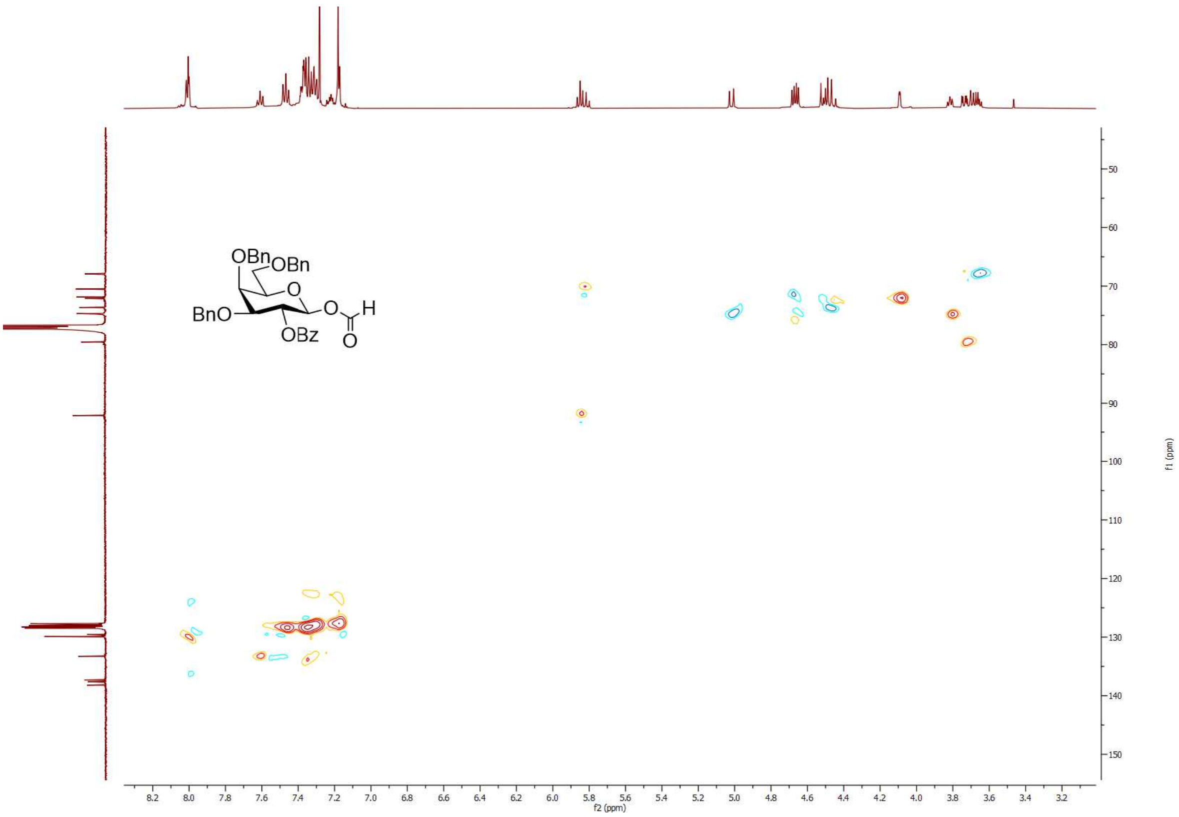

^{1}H NMR (500 MHz, $CDCl_3$)

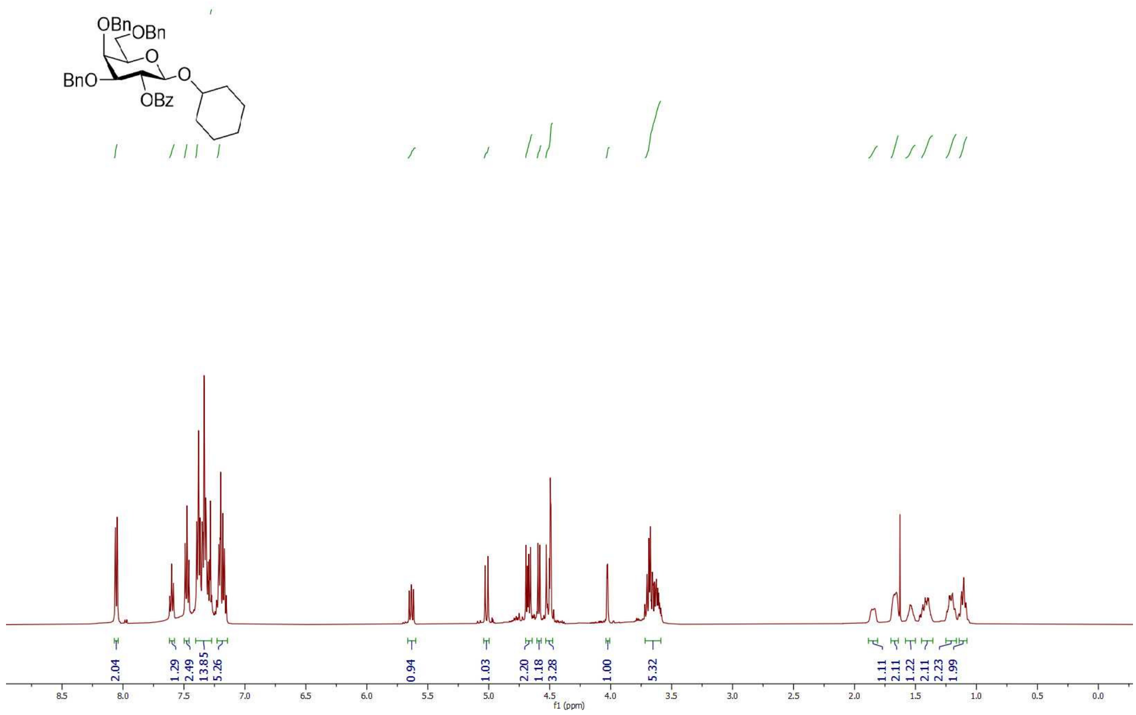

^{13}C NMR (126 MHz, $CDCl_3$)

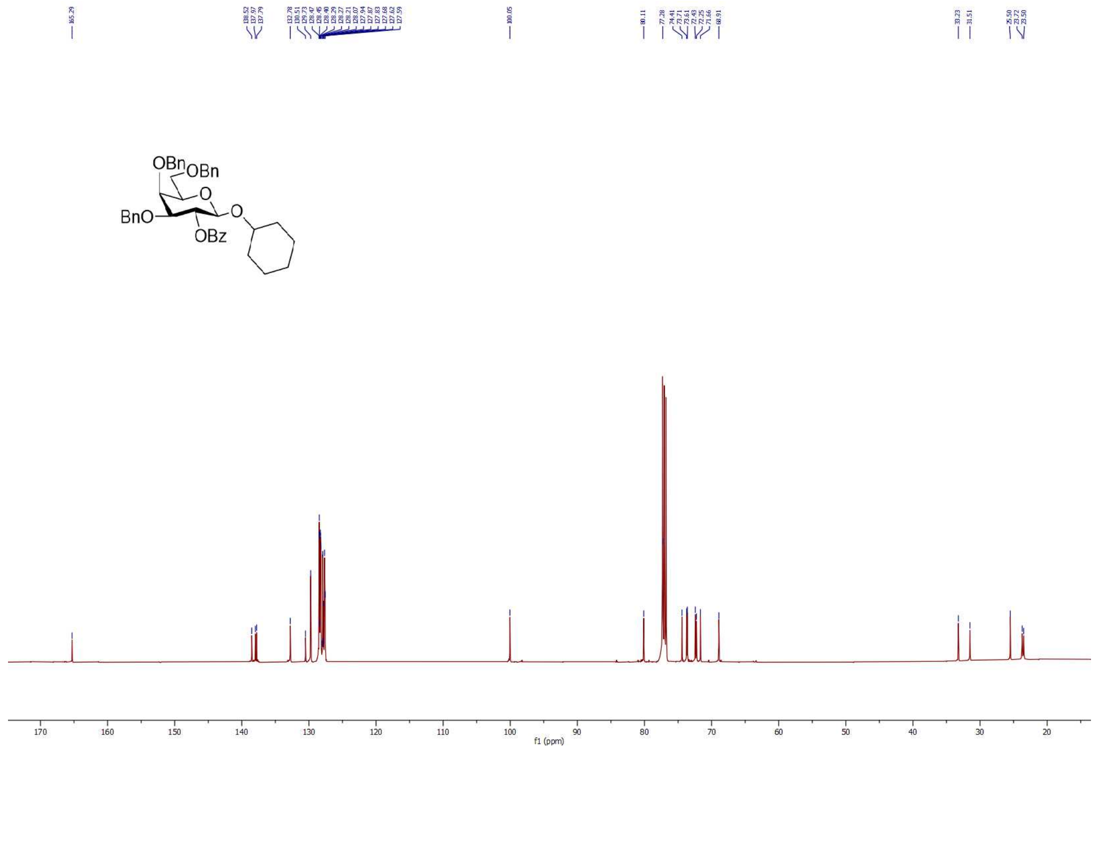

H-H COSY NMR

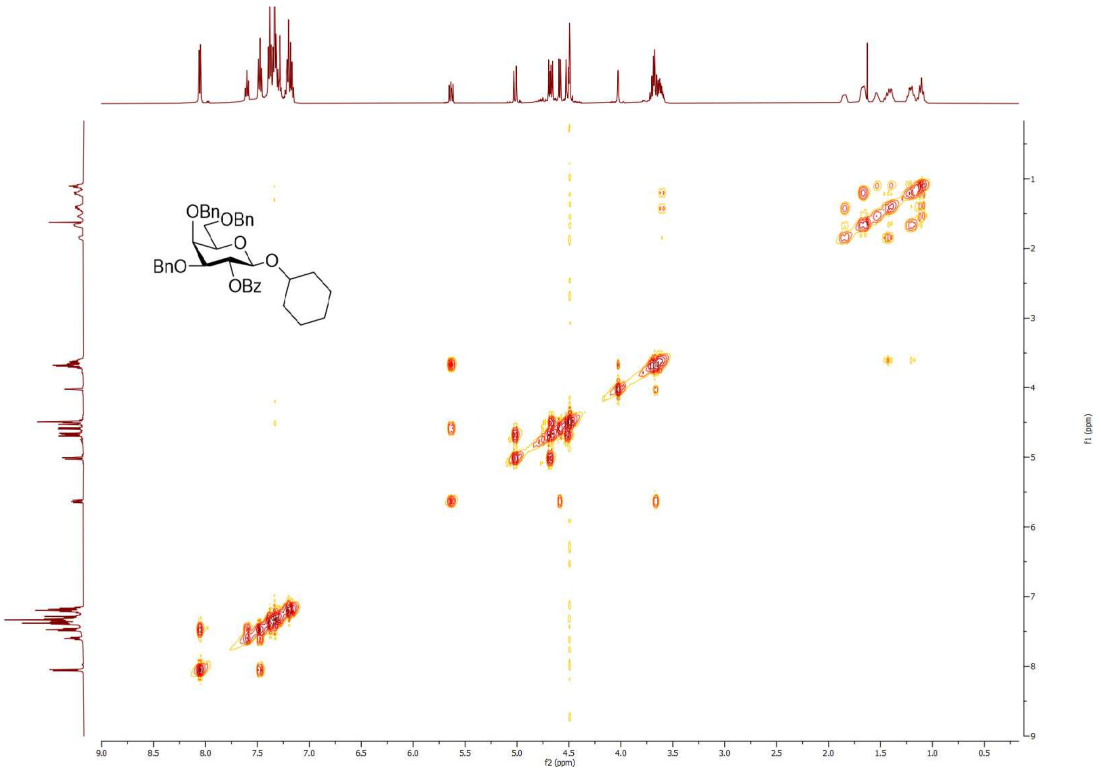

HSQC NMR

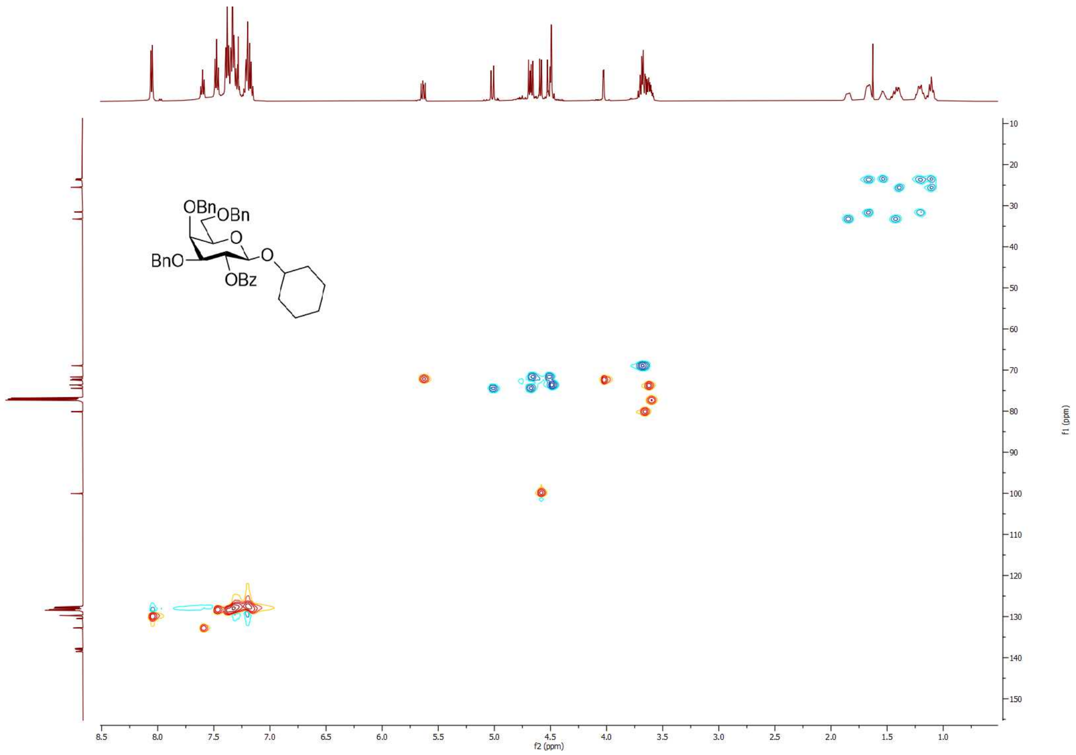

^{1}H NMR (500 MHz, $CDCl_3$)

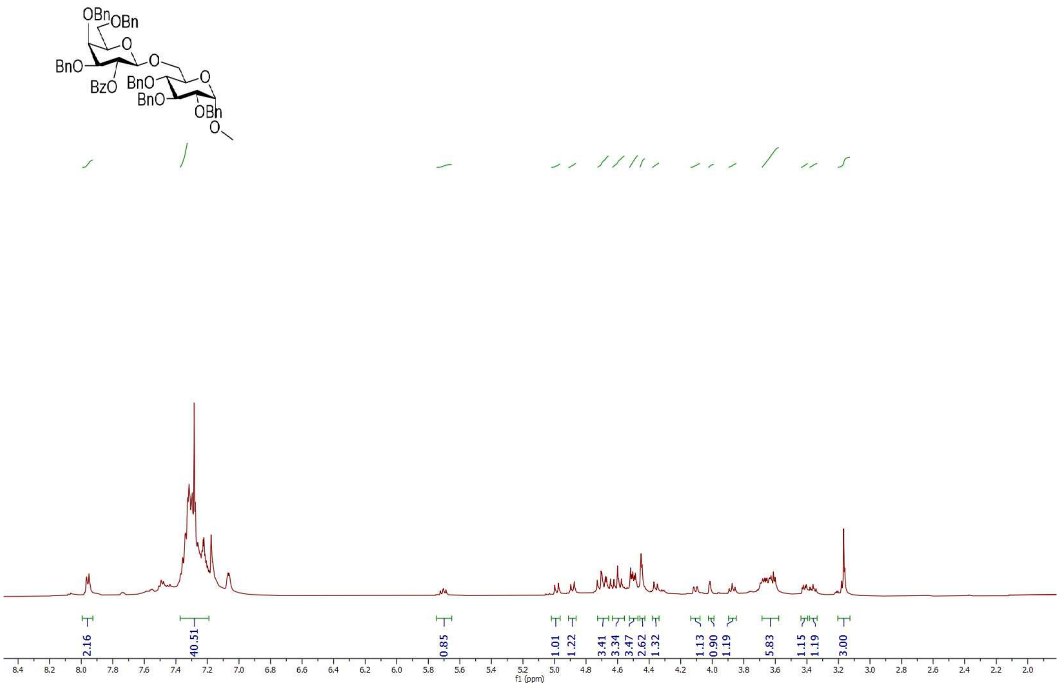

^{13}C NMR (126 MHz, $CDCl_3$)

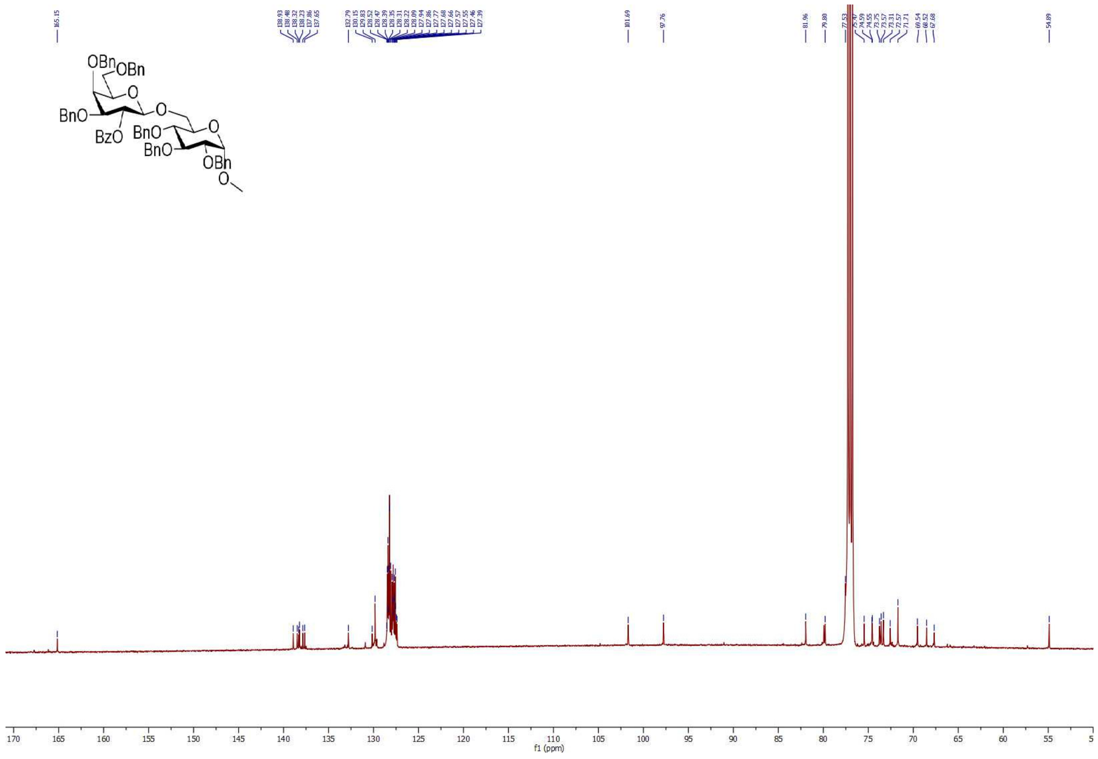

H-H COSY NMR

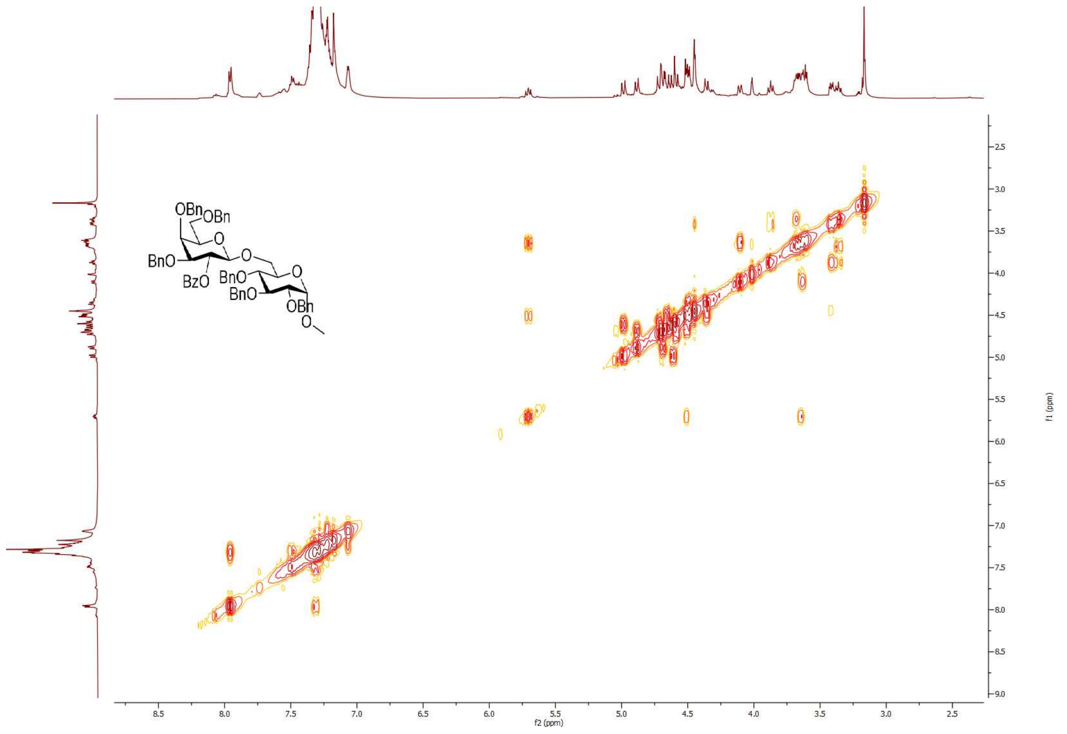

HSQC NMR

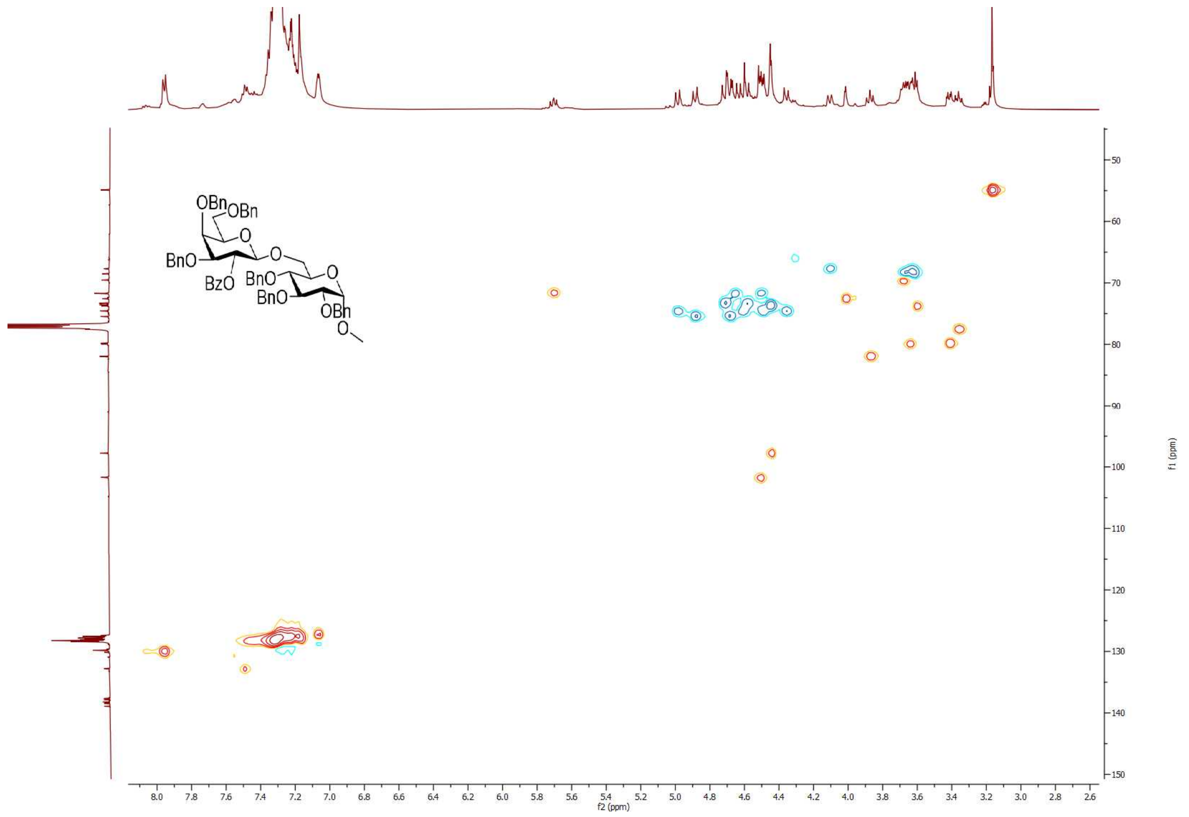

^{1}H NMR (500 MHz, $CDCl_3$)

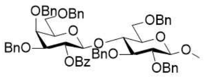

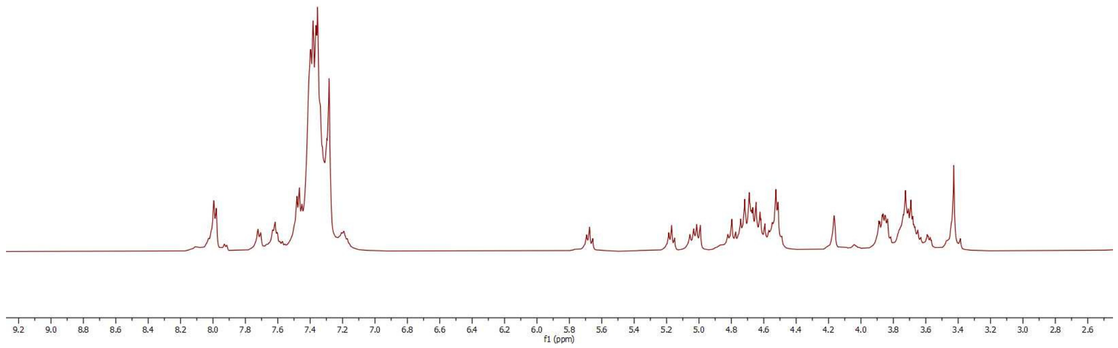

^{13}C NMR (126 MHz, $CDCl_3$)

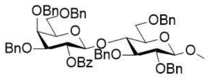

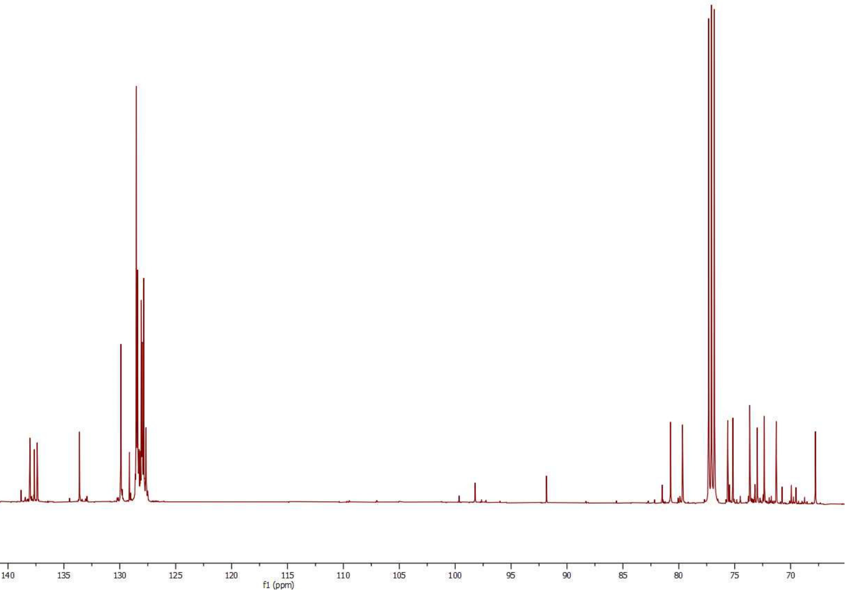

H-H COSY NMR

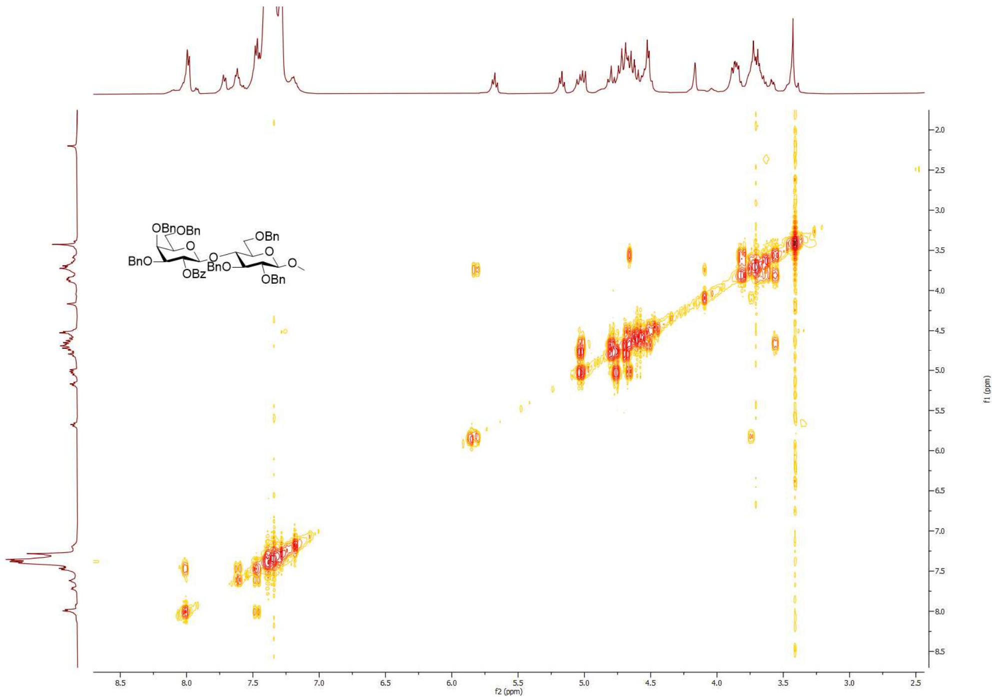

HSQC NMR

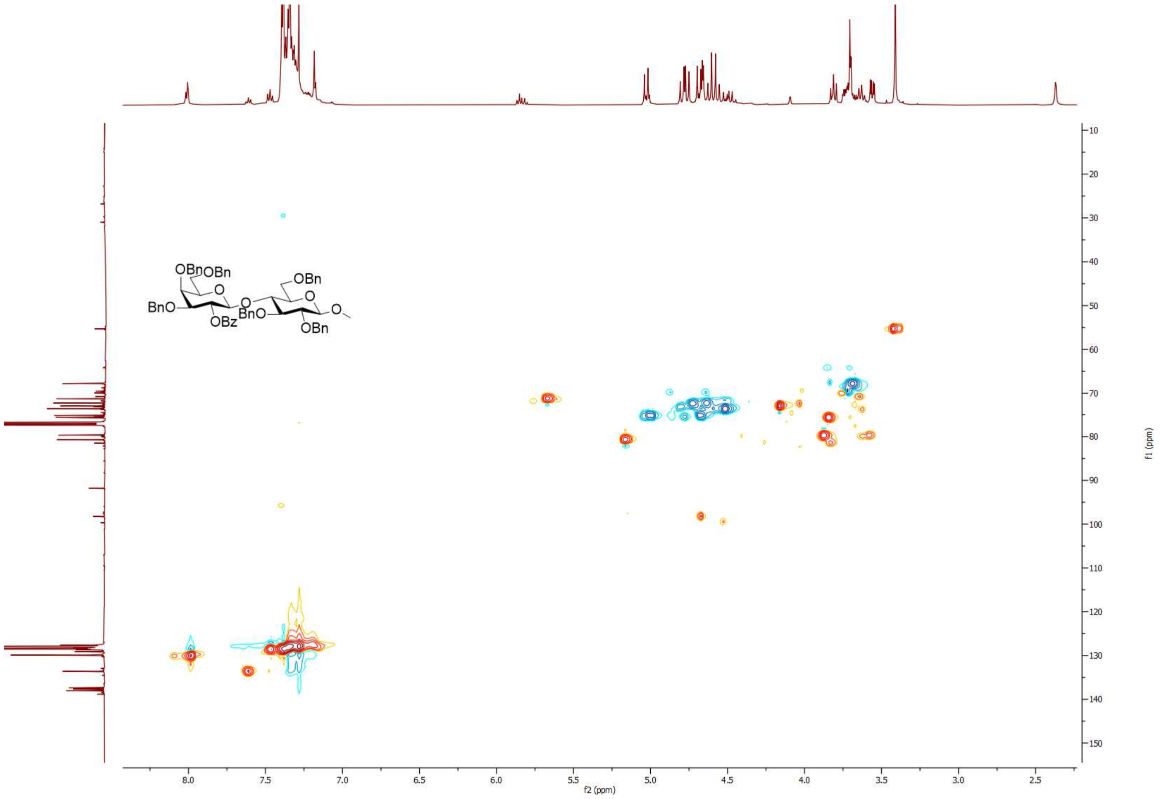

^{1}H NMR (500 MHz, $CDCl_3$)

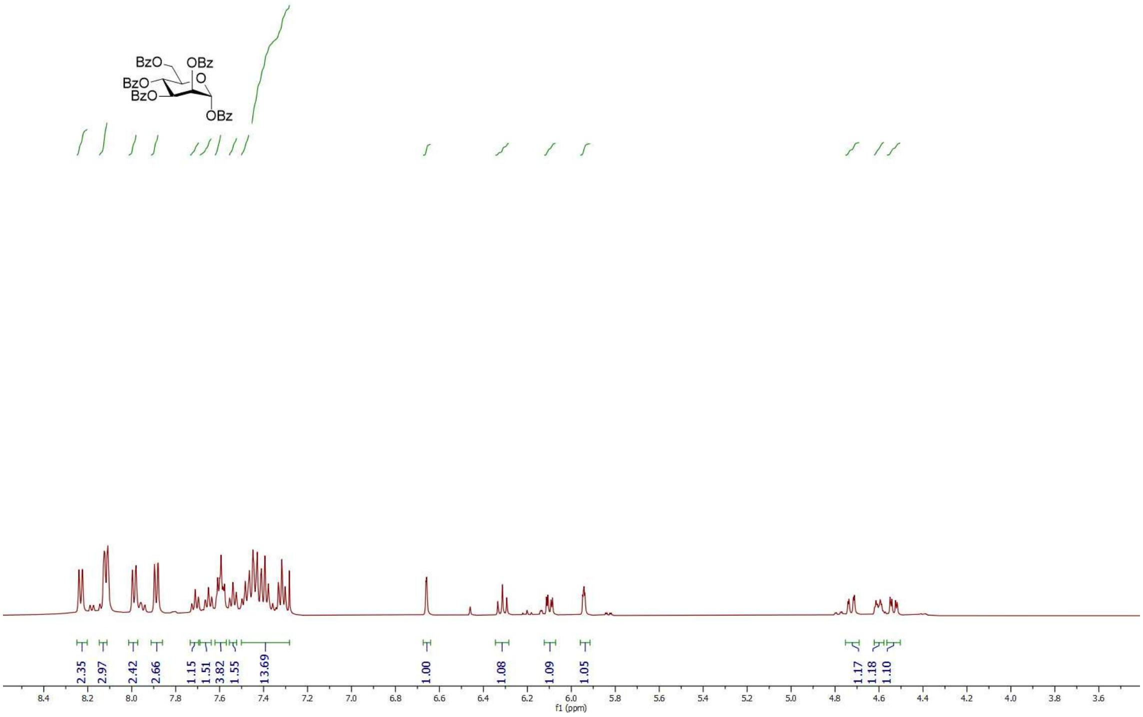

^{13}C NMR (126 MHz, $CDCl_3$)

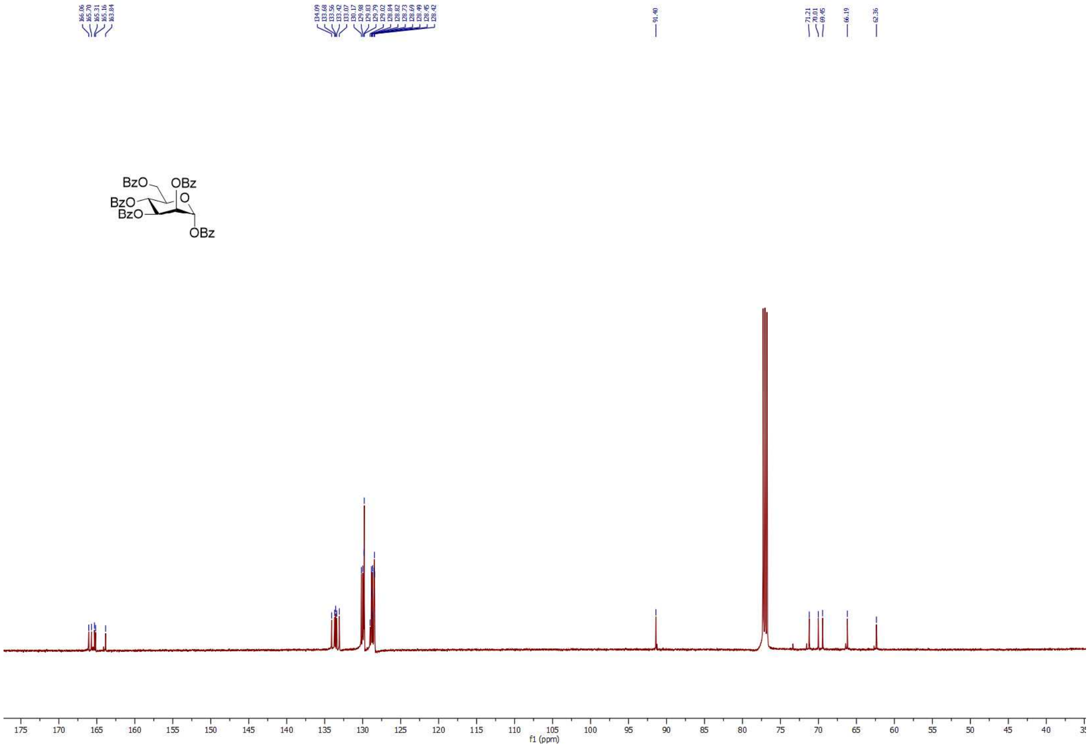

H-H COSY NMR

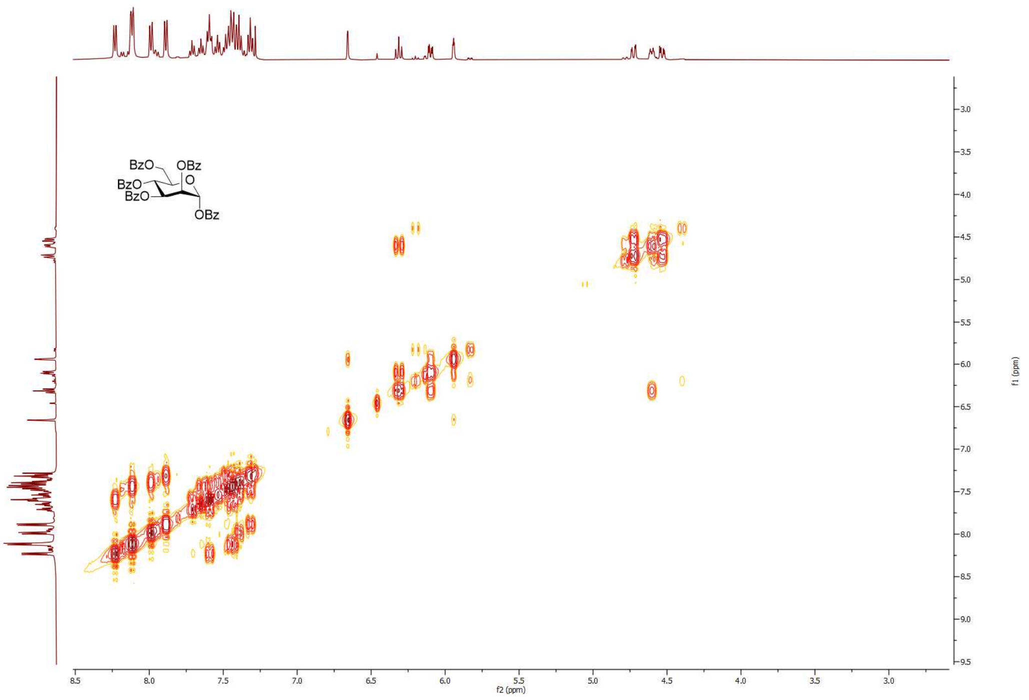

HSQC NMR

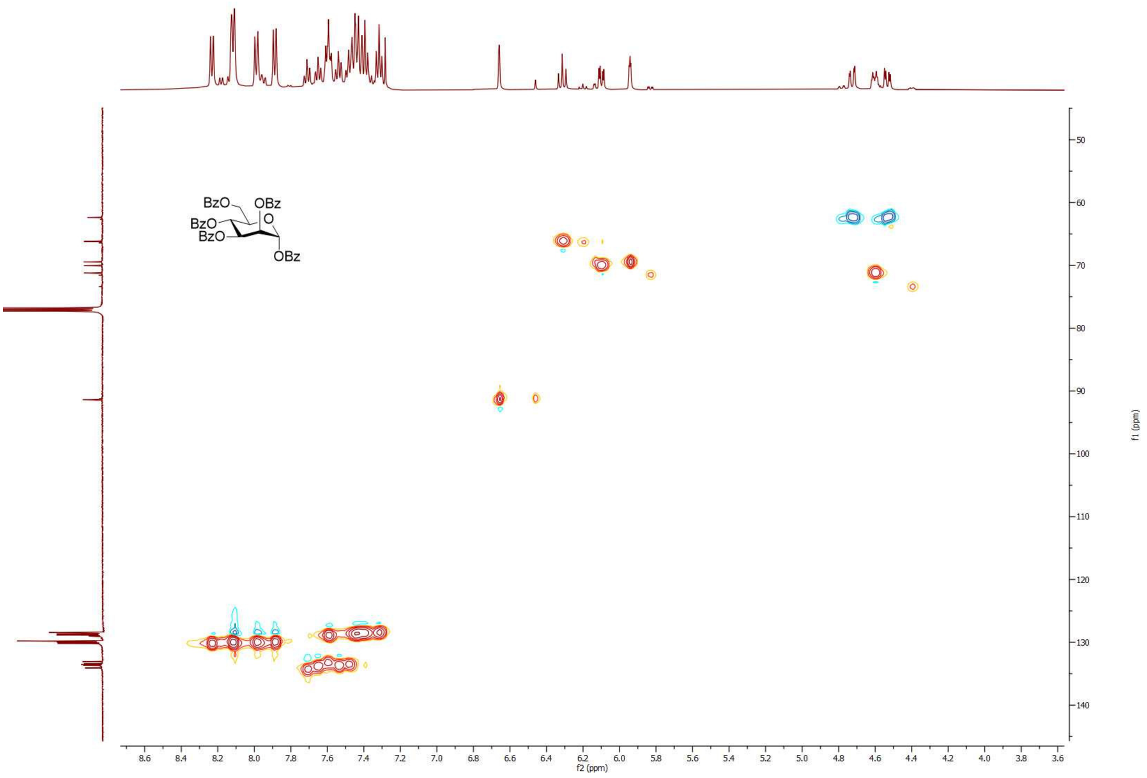

^{1}H NMR (500 MHz, $CDCl_3$)

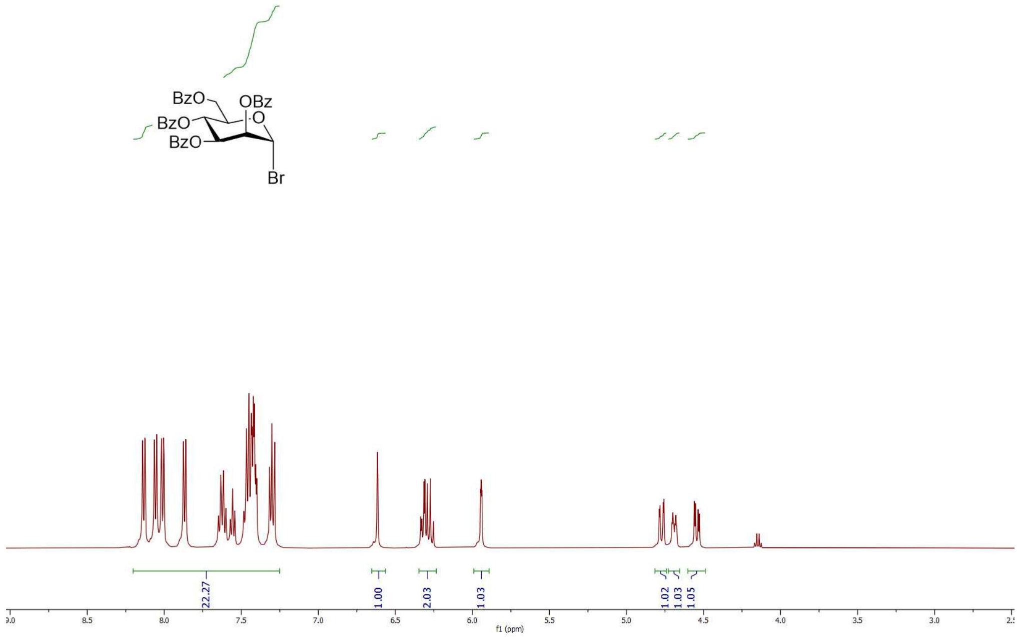

^{13}C NMR (126 MHz, $CDCl_3$)

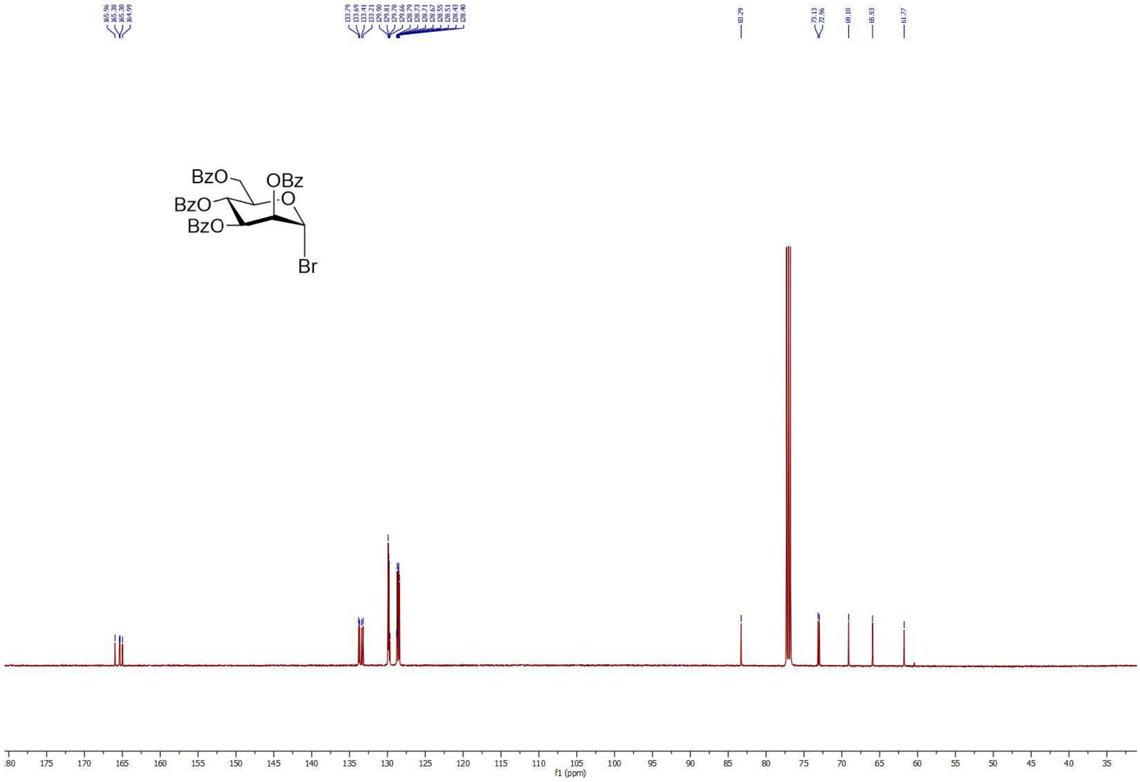

H-H COSY NMR

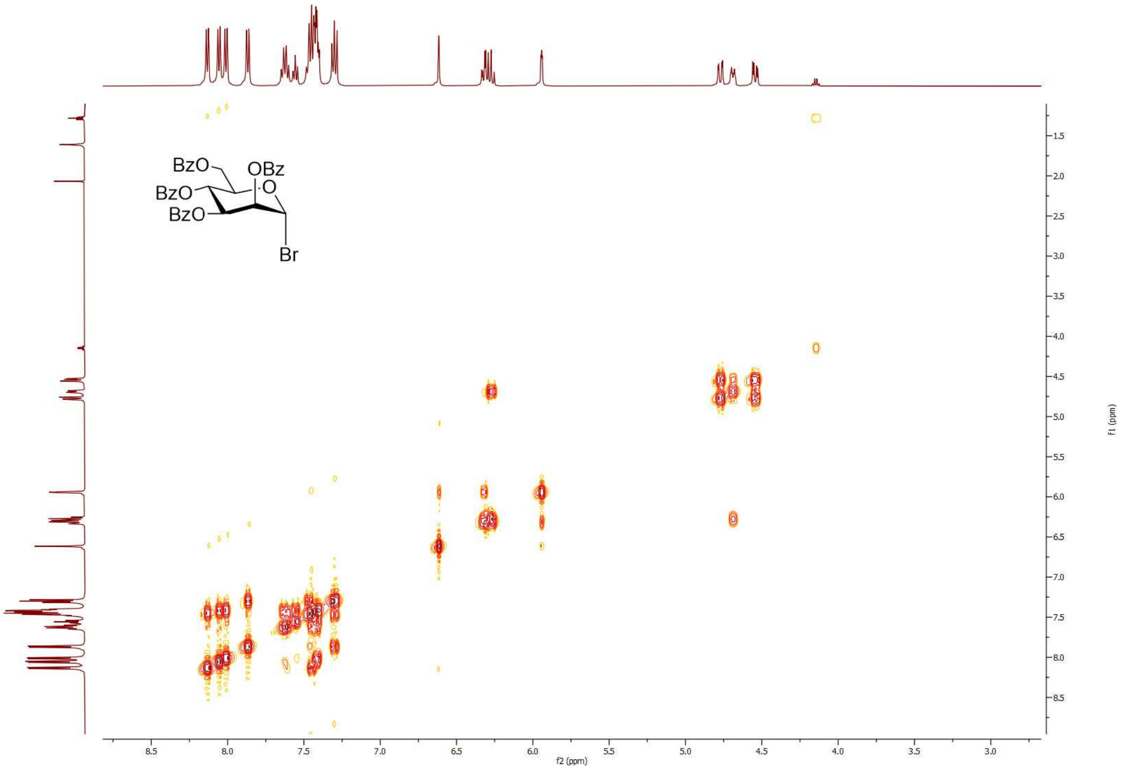

HSQC NMR

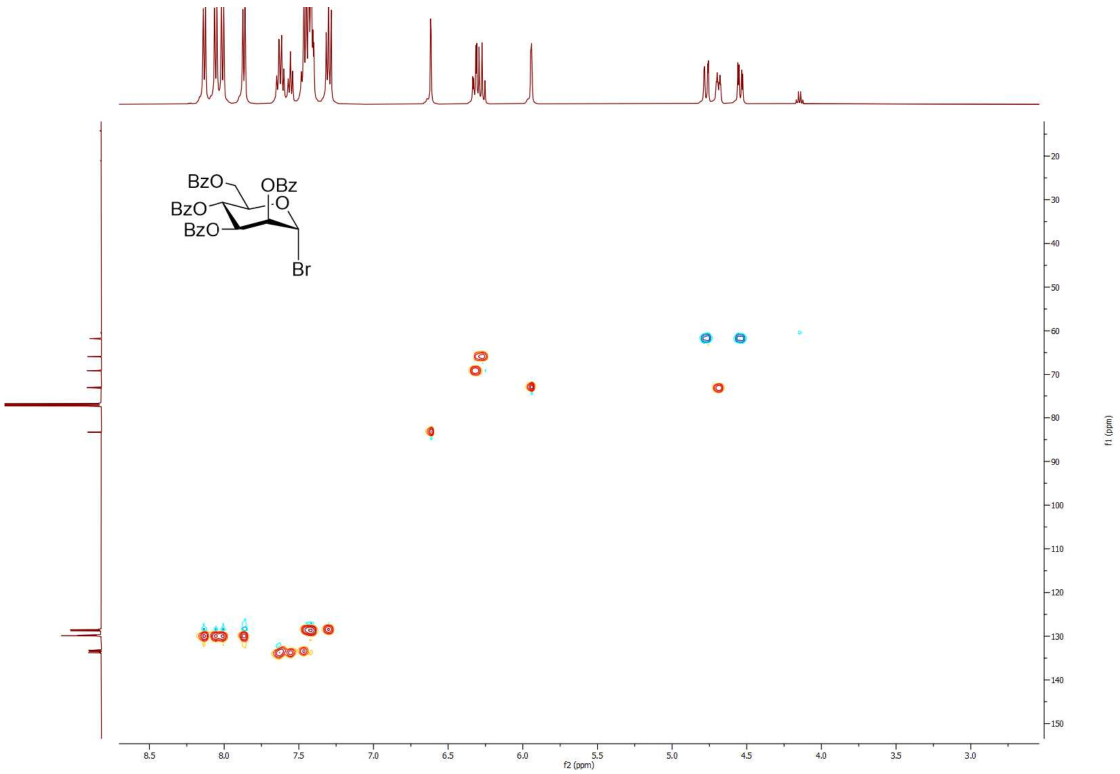

^{1}H NMR (500 MHz, $CDCl_3$)

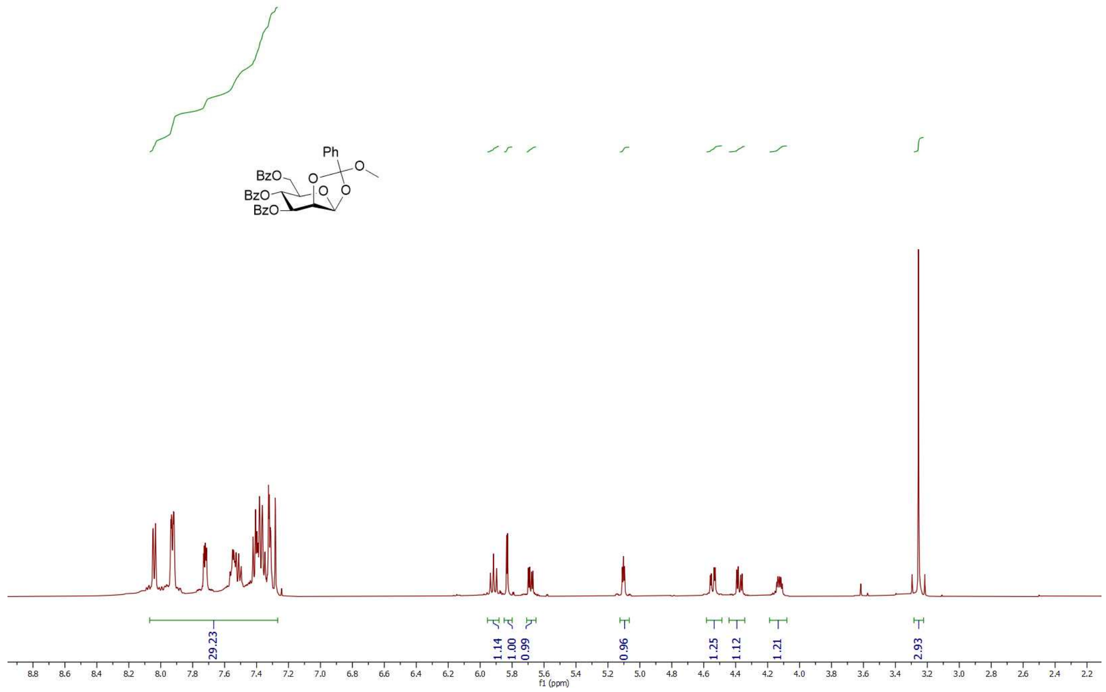

^{13}C NMR (126 MHz, $CDCl_3$)

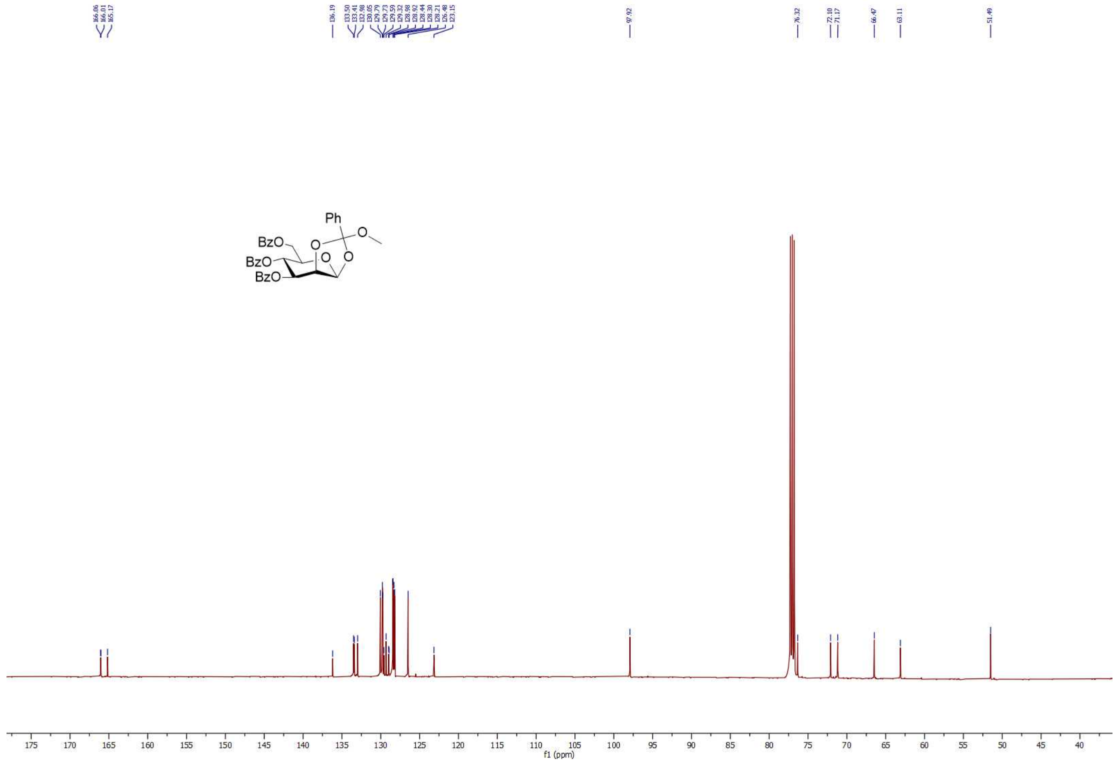

H-H COSY NMR

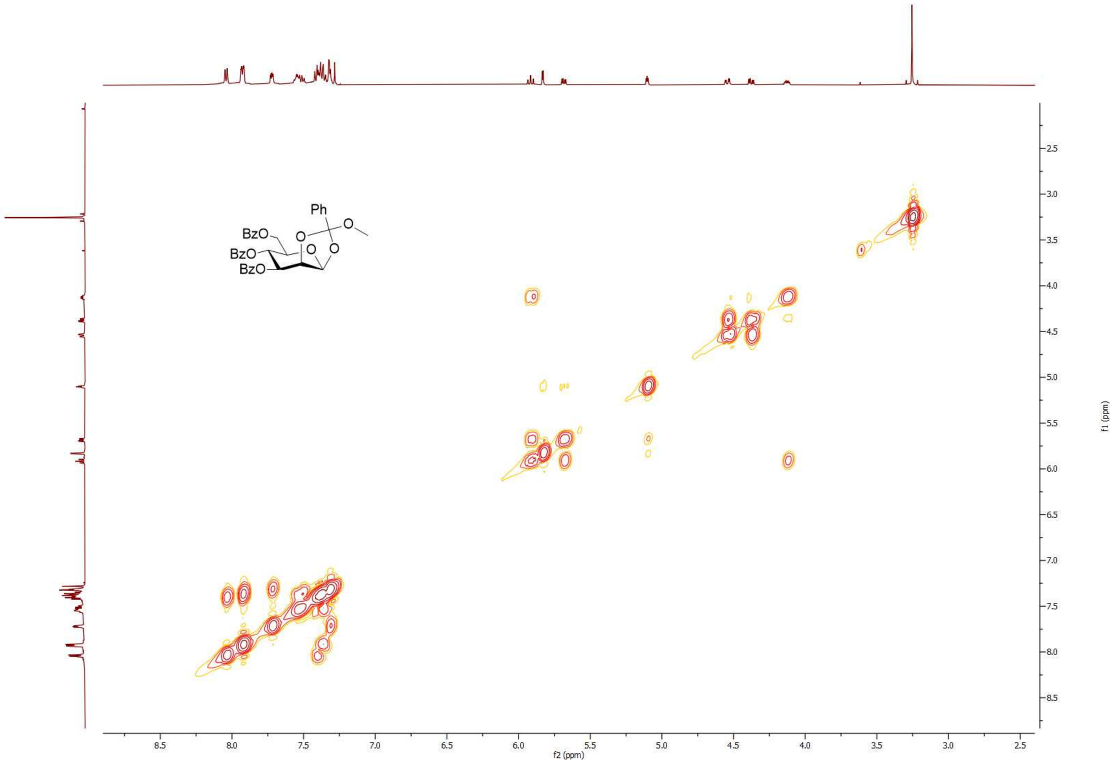

HSQC NMR

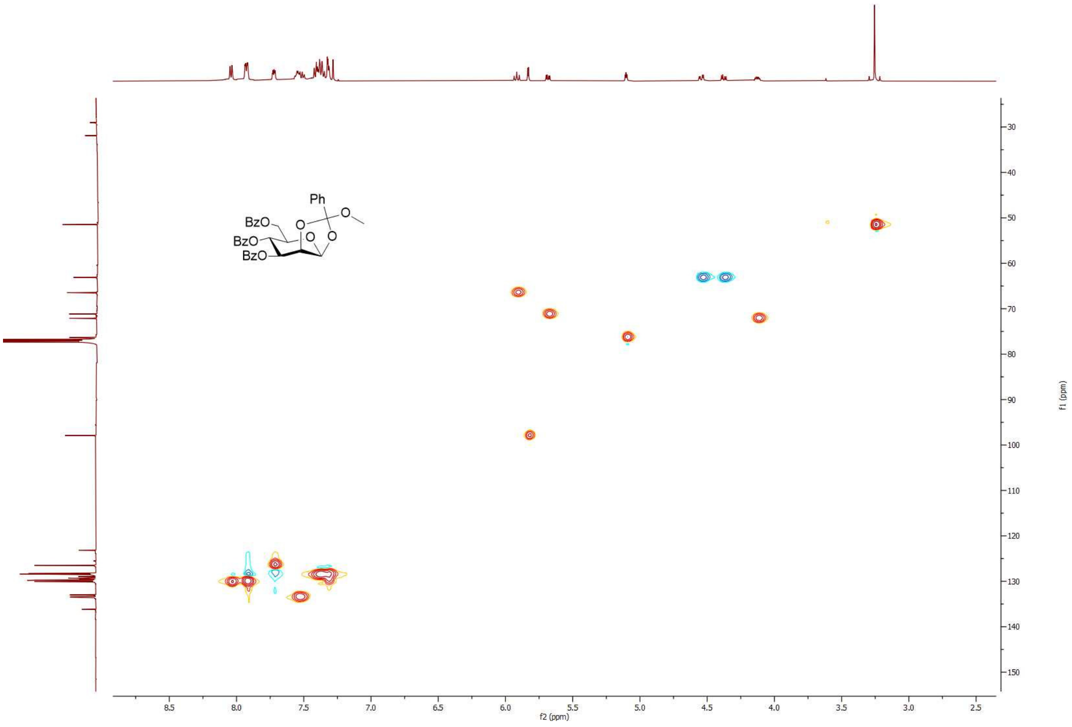

^{1}H NMR (500 MHz, $CDCl_3$)

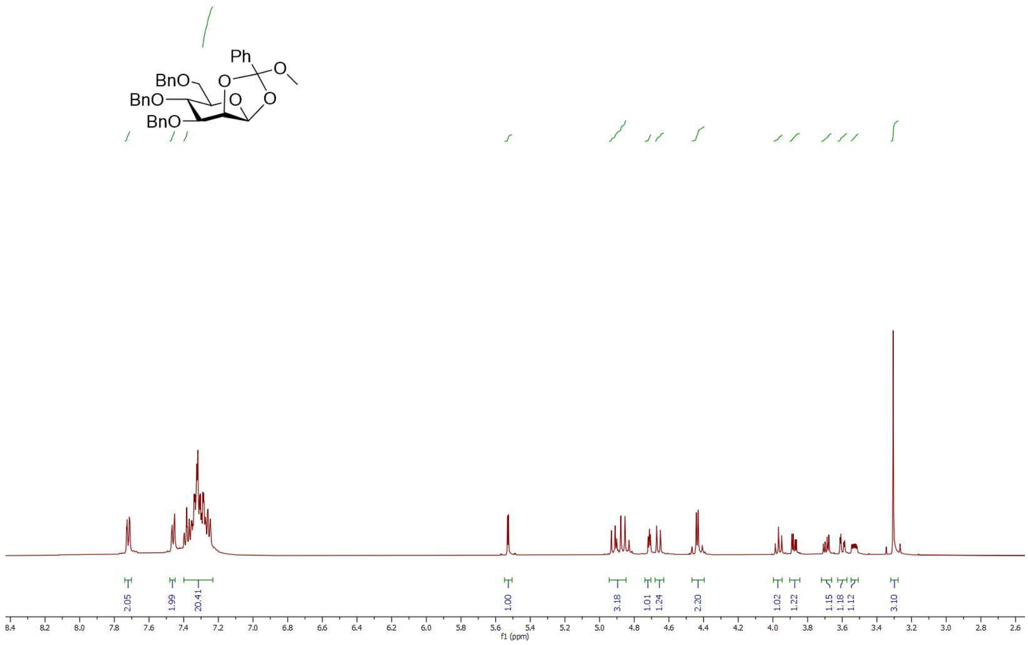

^{13}C NMR (126 MHz, $CDCl_3$)

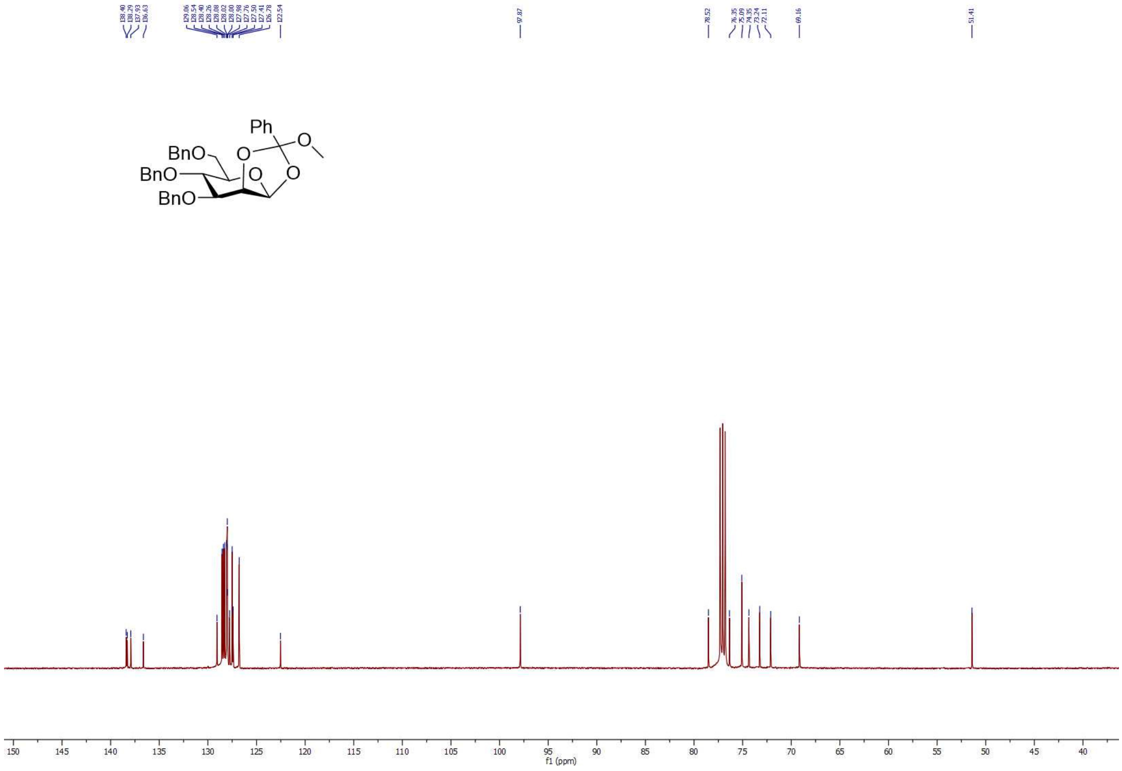

H-H COSY NMR

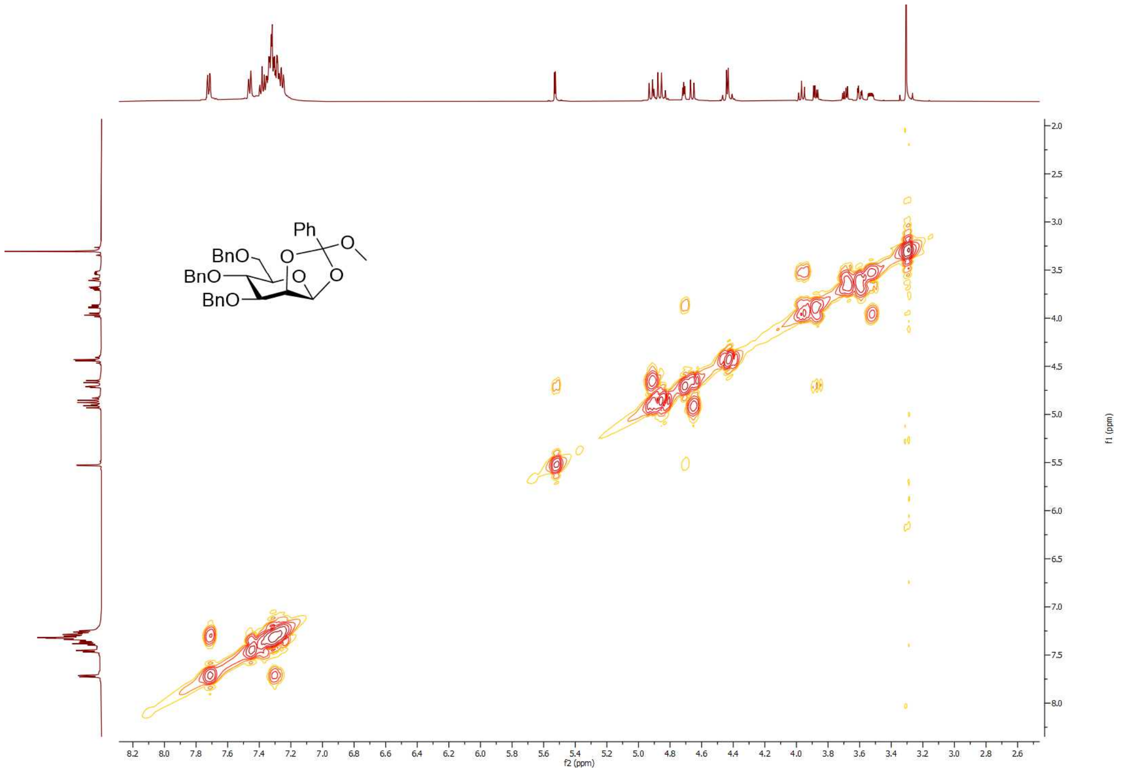
Ph
BnO
BnO
BnO
O
O
O
O
f2 (ppm)
f1 (ppm)

HSQC NMR

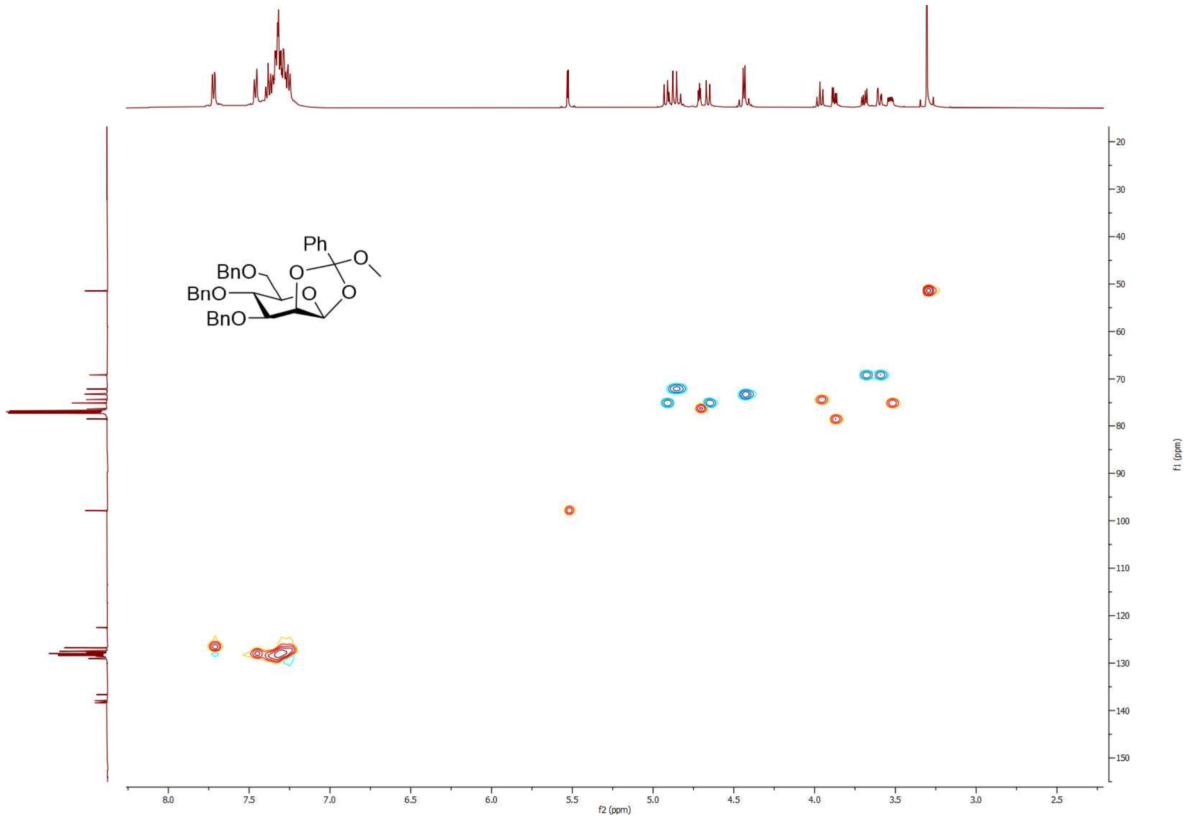
Ph
BnO
BnO
BnO
O
O
O
O
f2 (ppm)
f1 (ppm)

^{1}H NMR (500 MHz, $CDCl_3$)

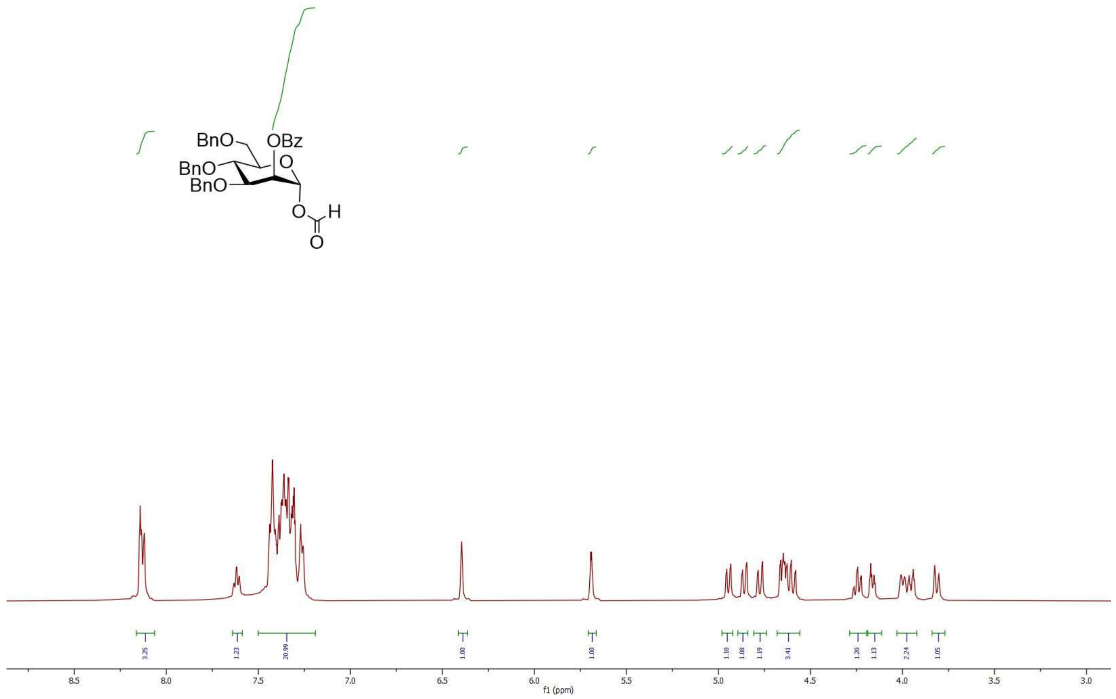

^{13}C NMR (126 MHz, $CDCl_3$)

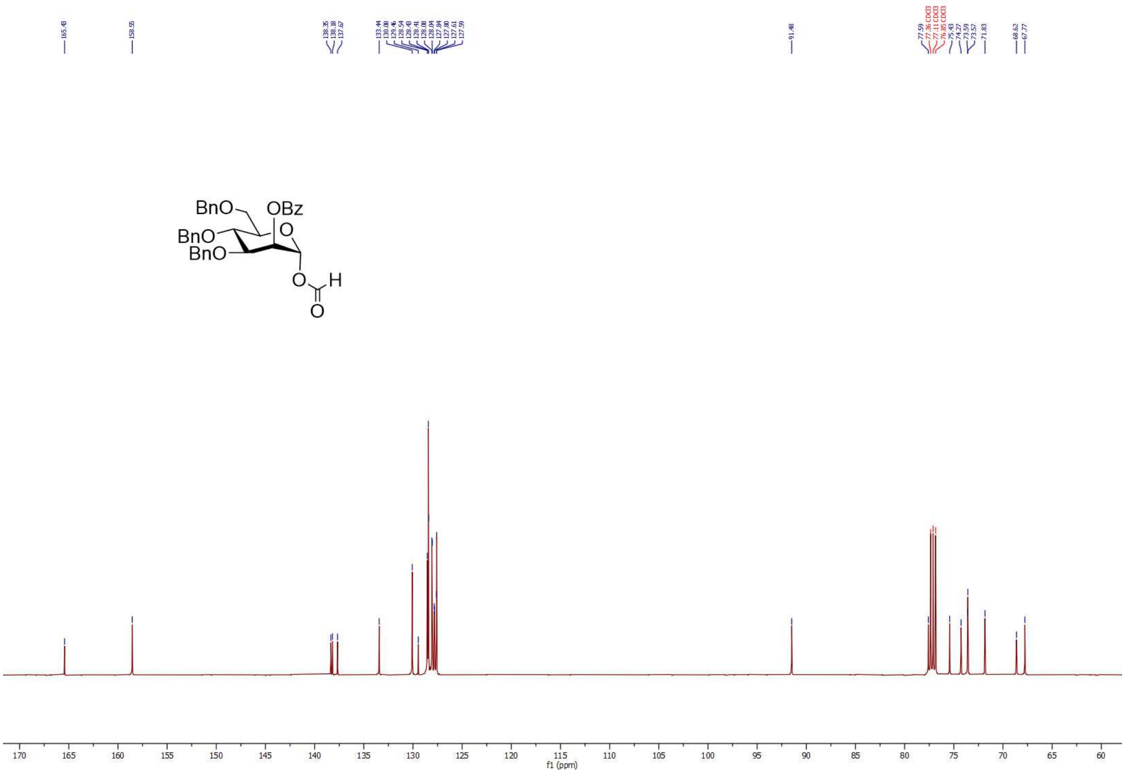

H-H COSY NMR

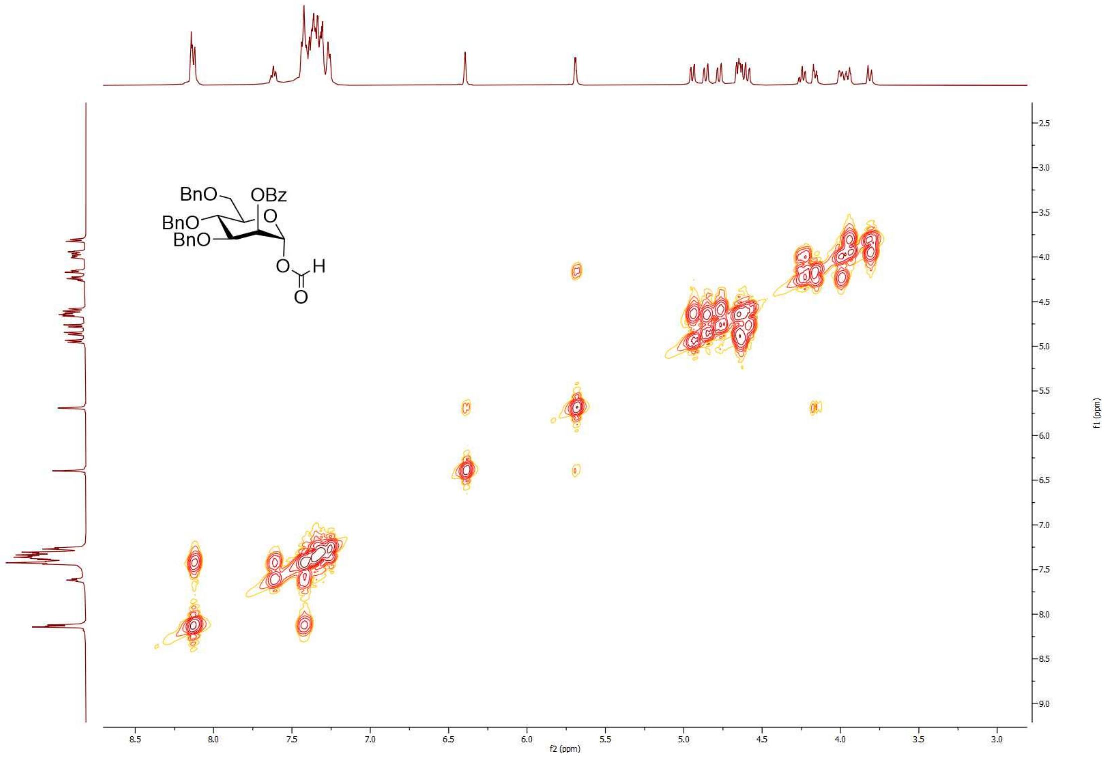

HSQC NMR

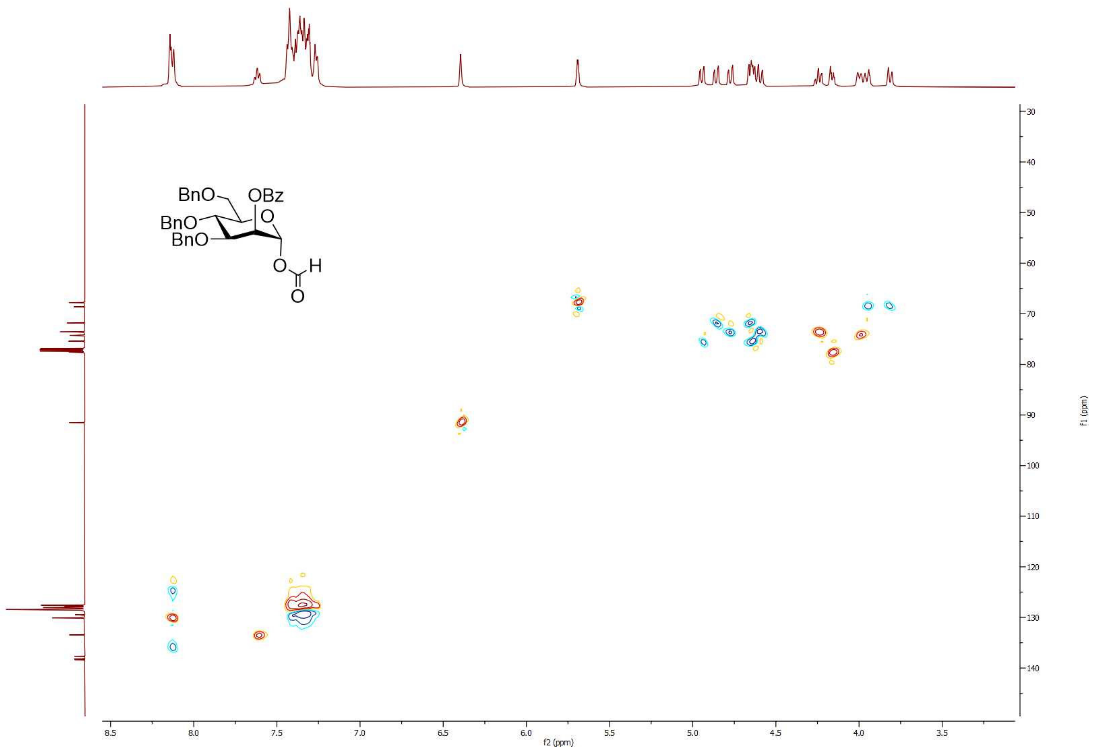

^{1}H NMR (500 MHz, $CDCl_3$)

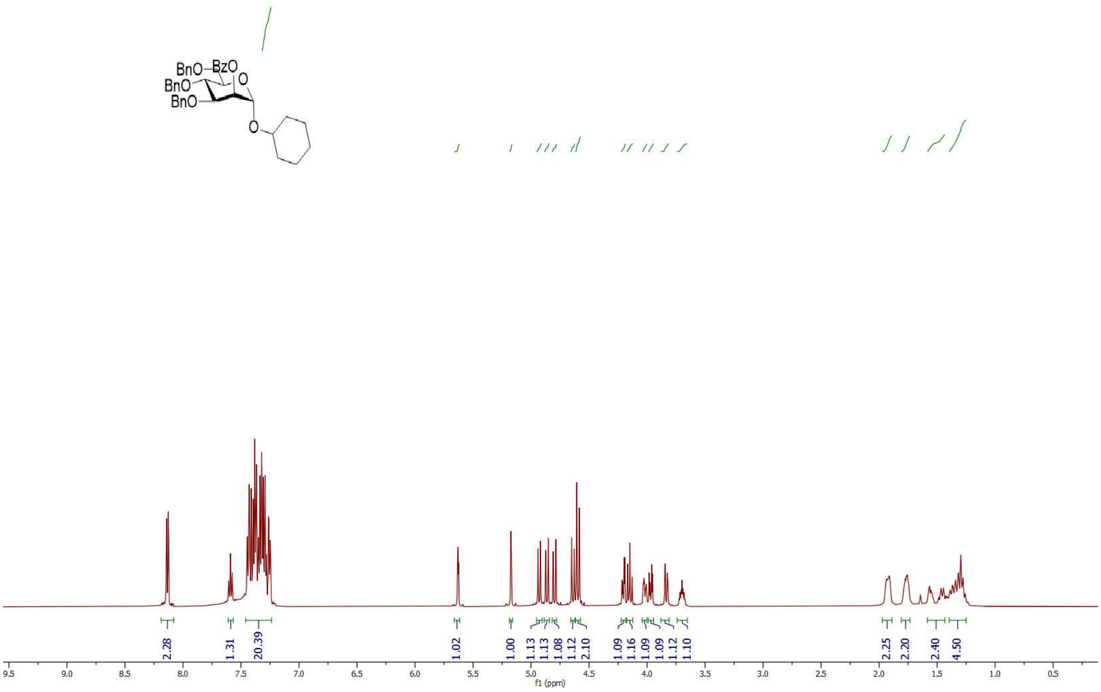

^{13}C NMR (126 MHz, $CDCl_3$)

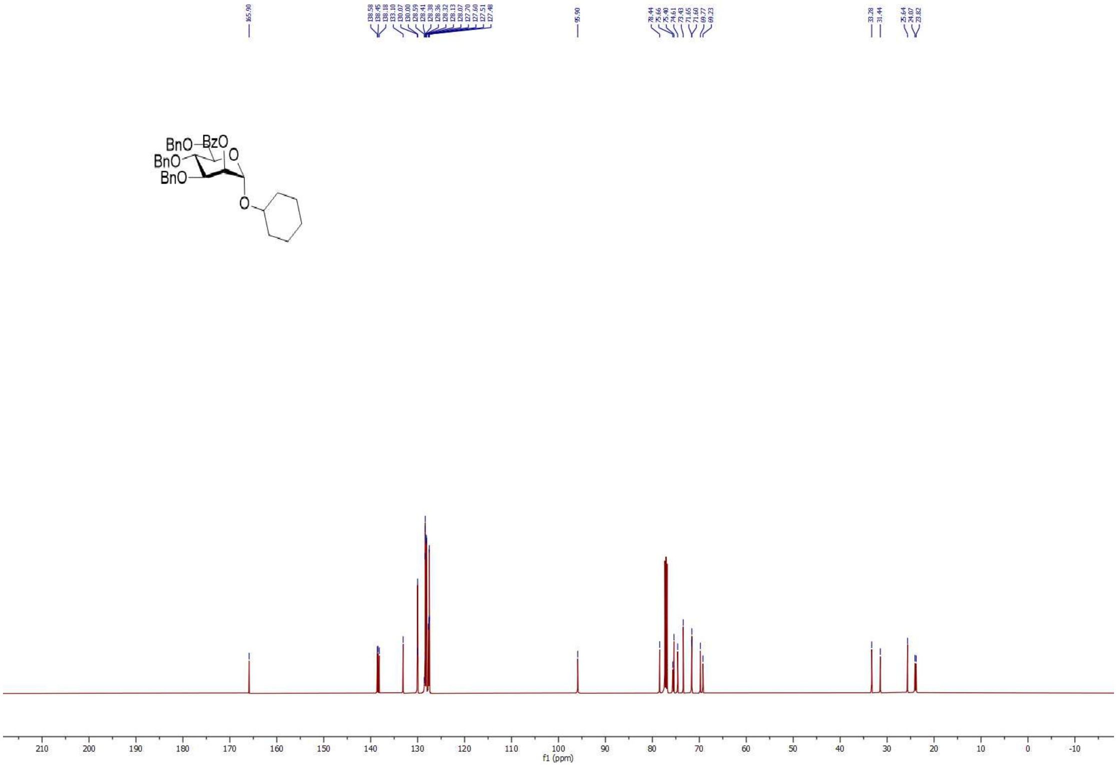

H-H COSY NMR

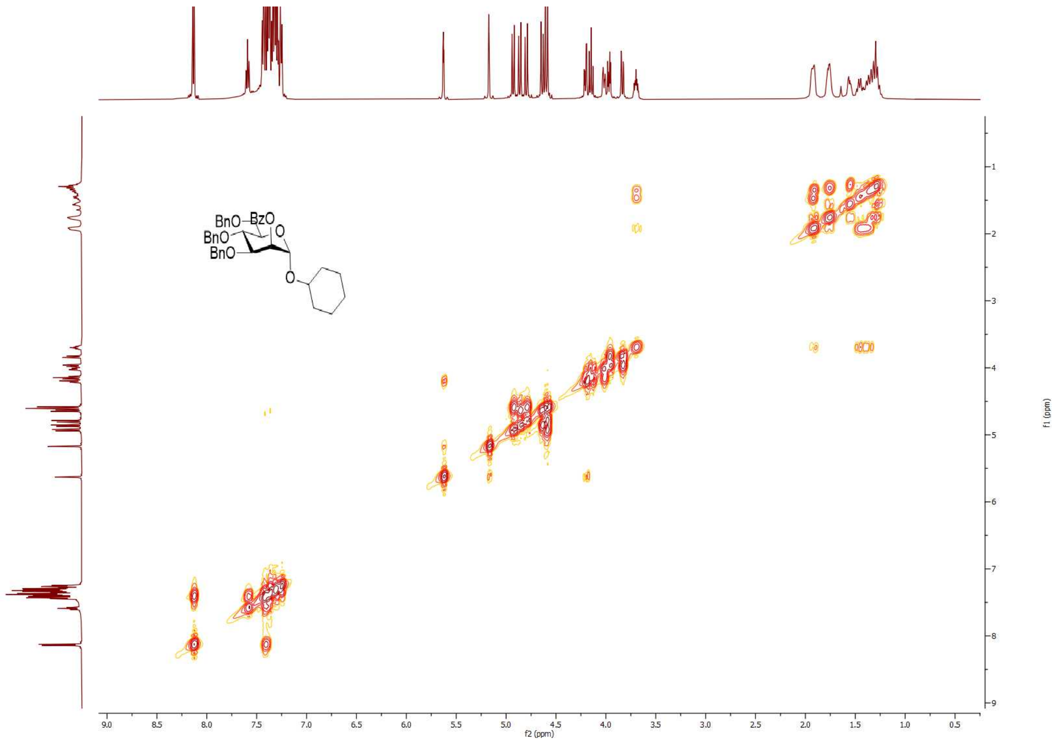

HSQC NMR

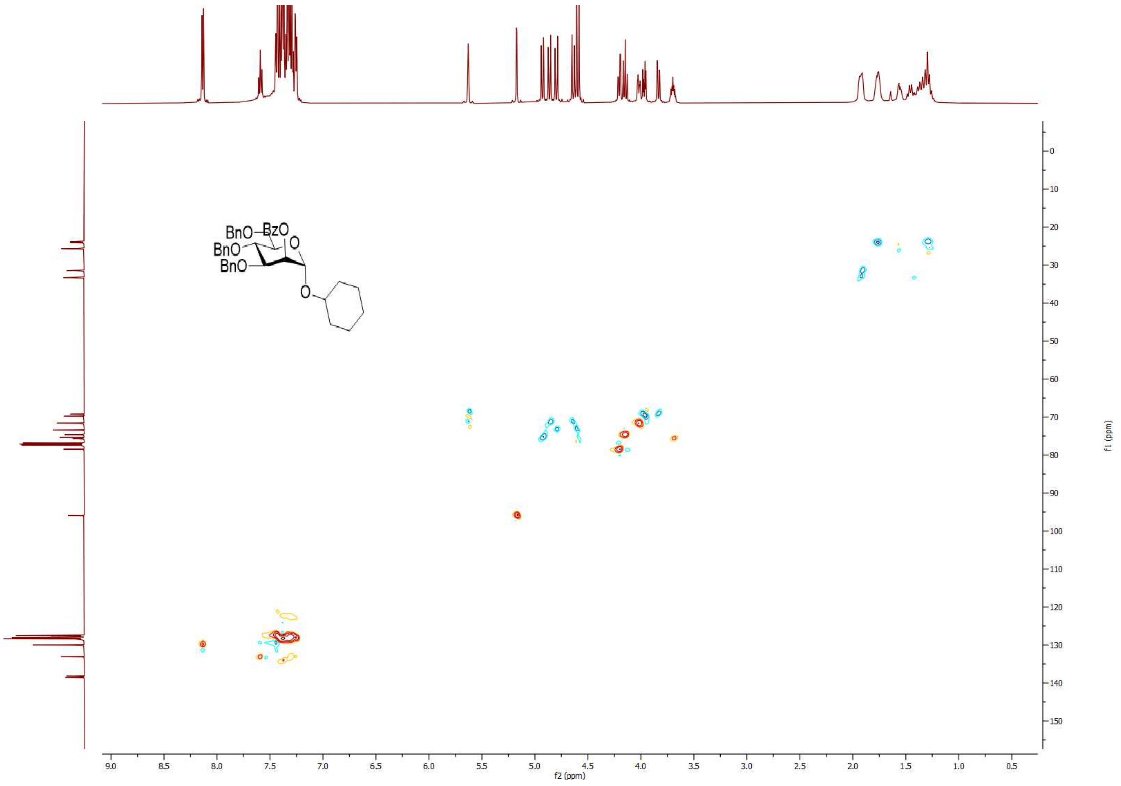

^1H NMR (500 MHz, $CDCl_3$)

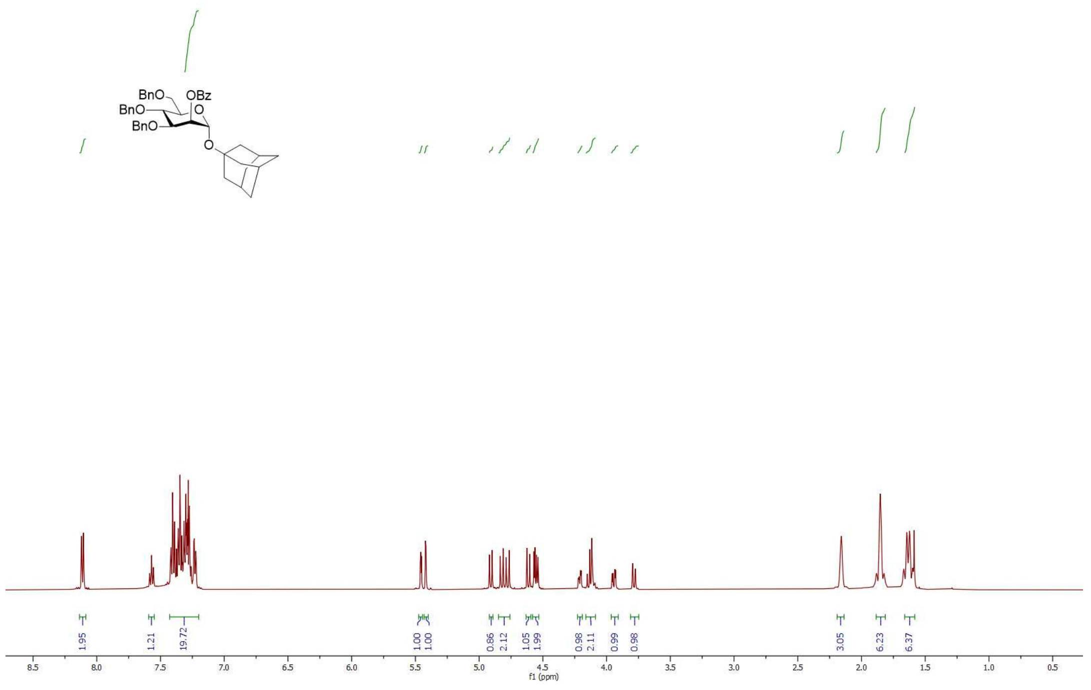

^{13}C NMR (126 MHz, $CDCl_3$)

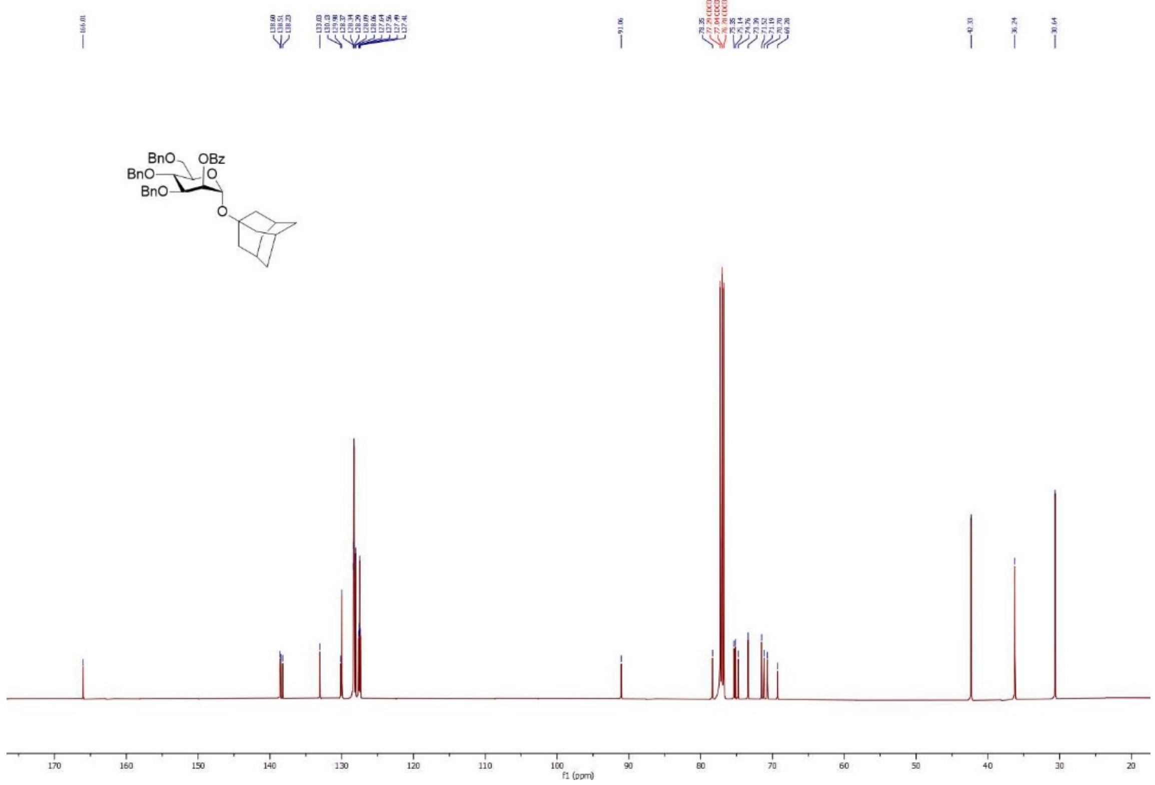

H-H COSY NMR

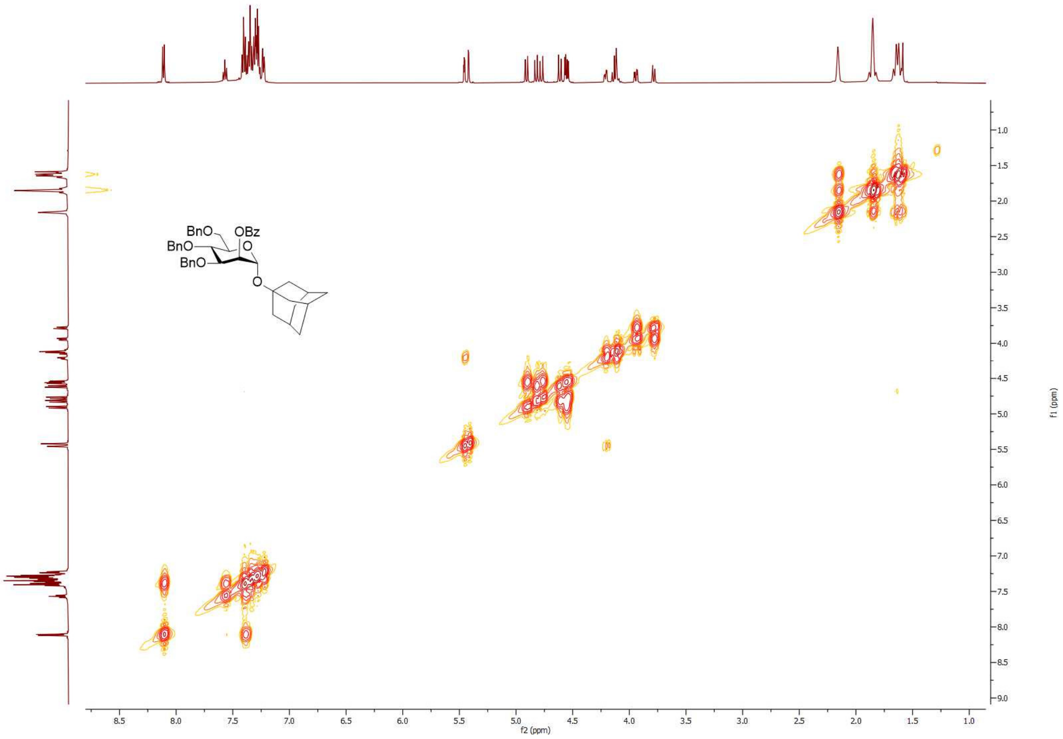

HSQC NMR

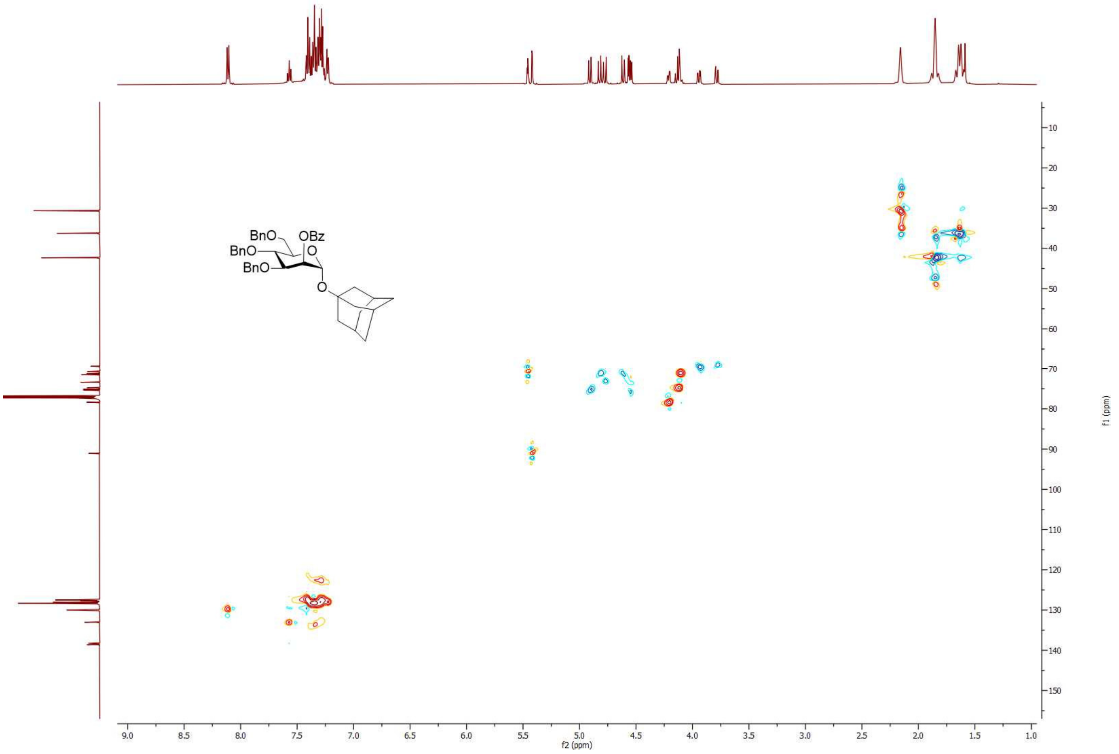

^{1}H NMR (500 MHz, $CDCl_3$)

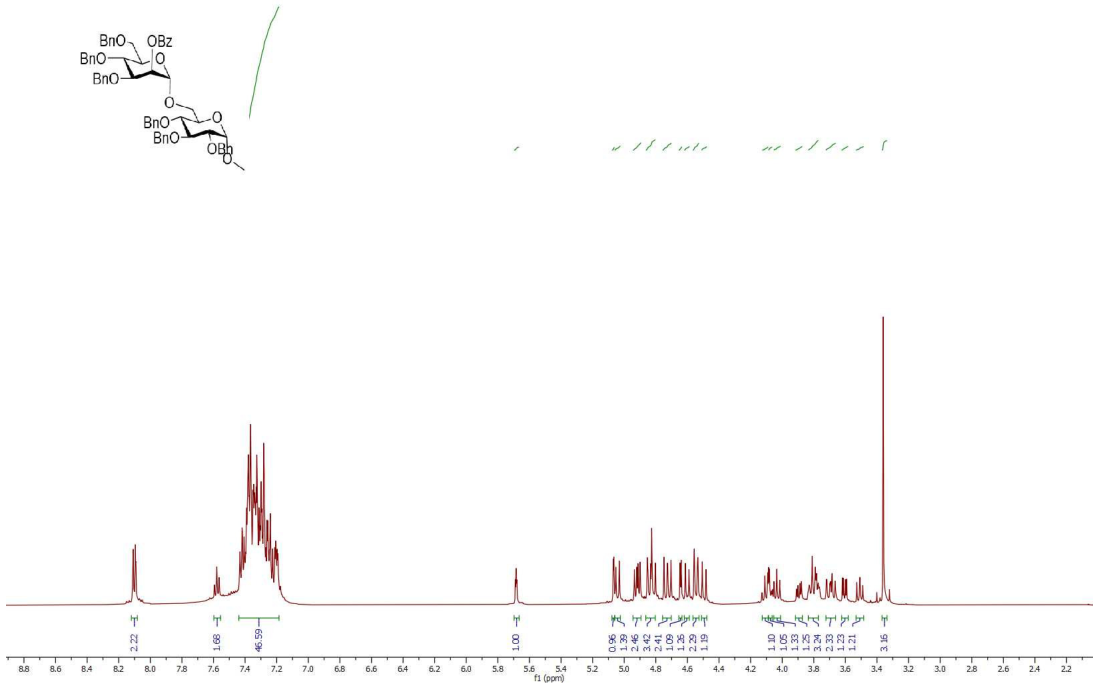

^{13}C NMR (126 MHz, $CDCl_3$)

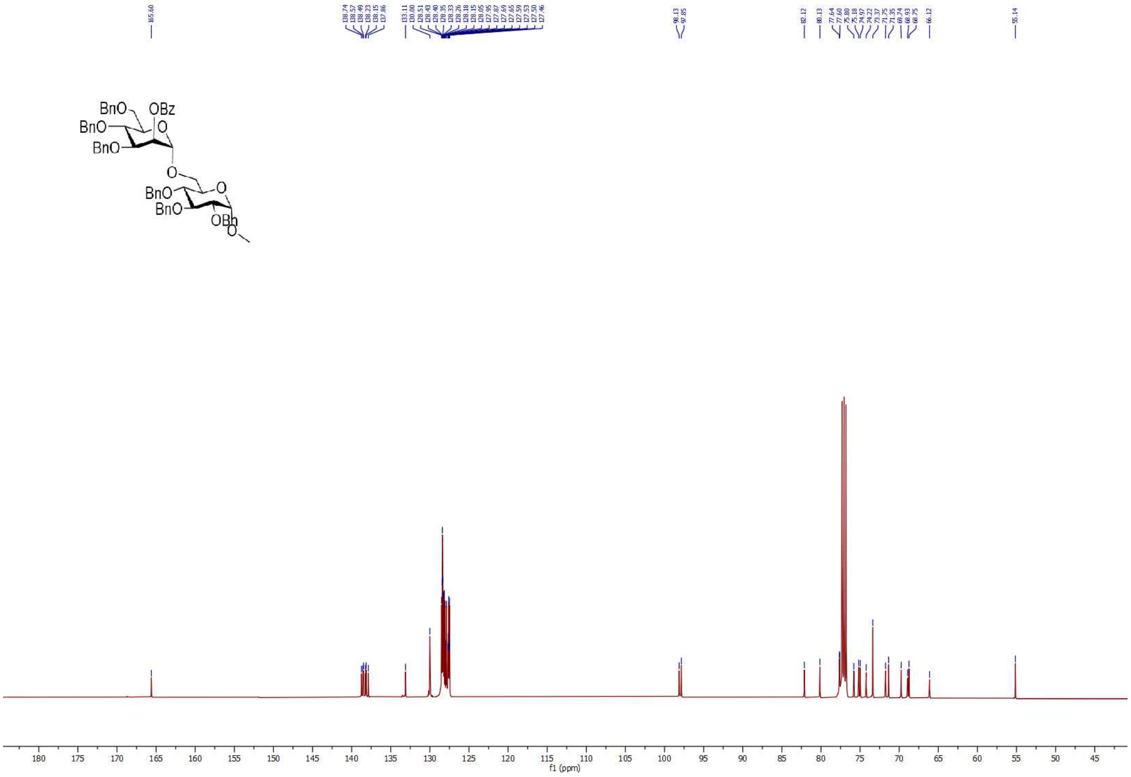

H-H COSY NMR

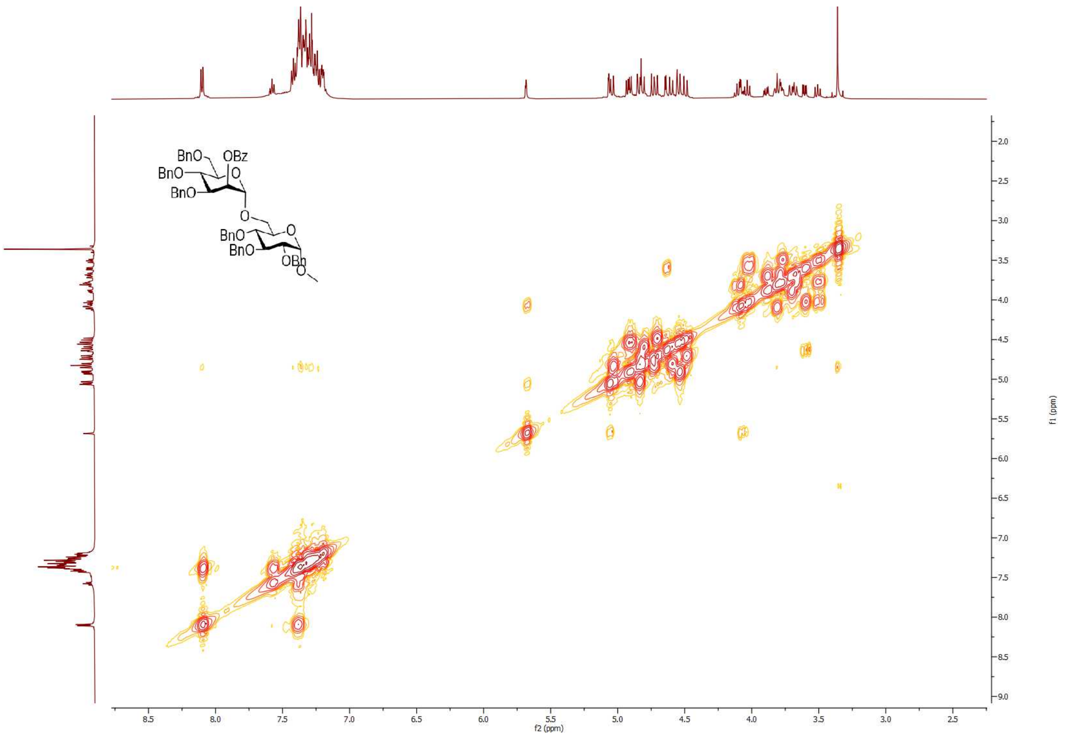

HSQC NMR

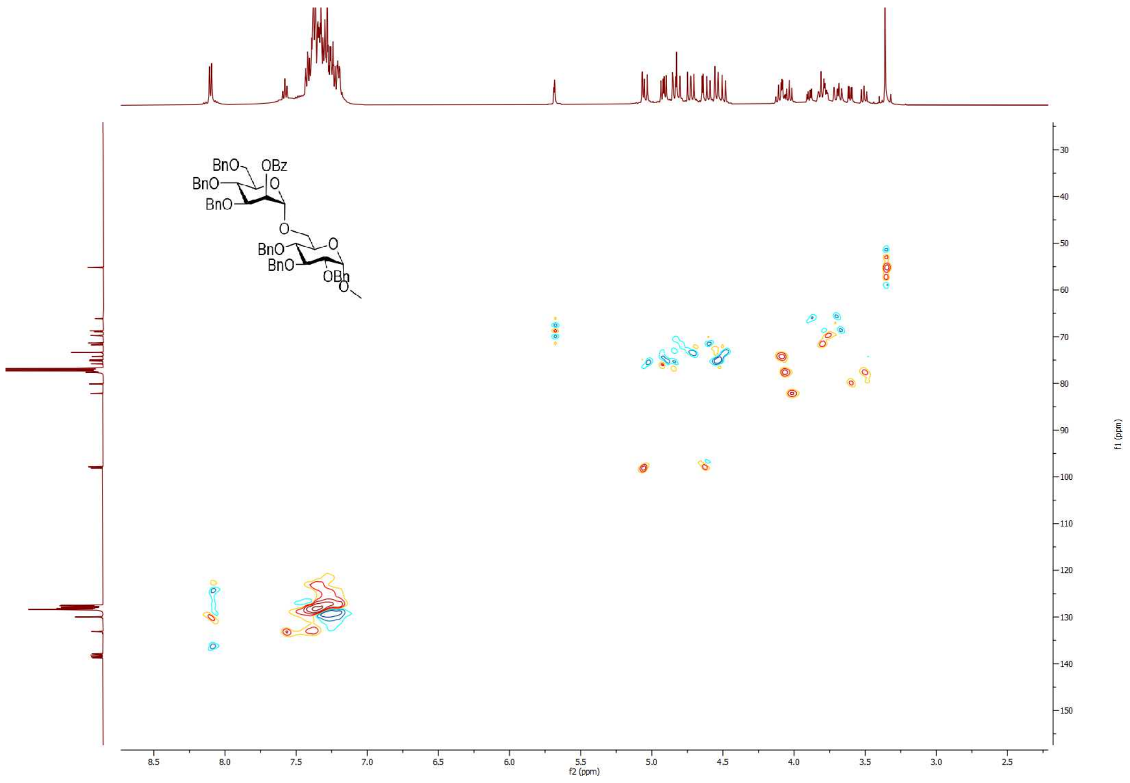

^{1}H NMR (500 MHz, $CDCl_3$)

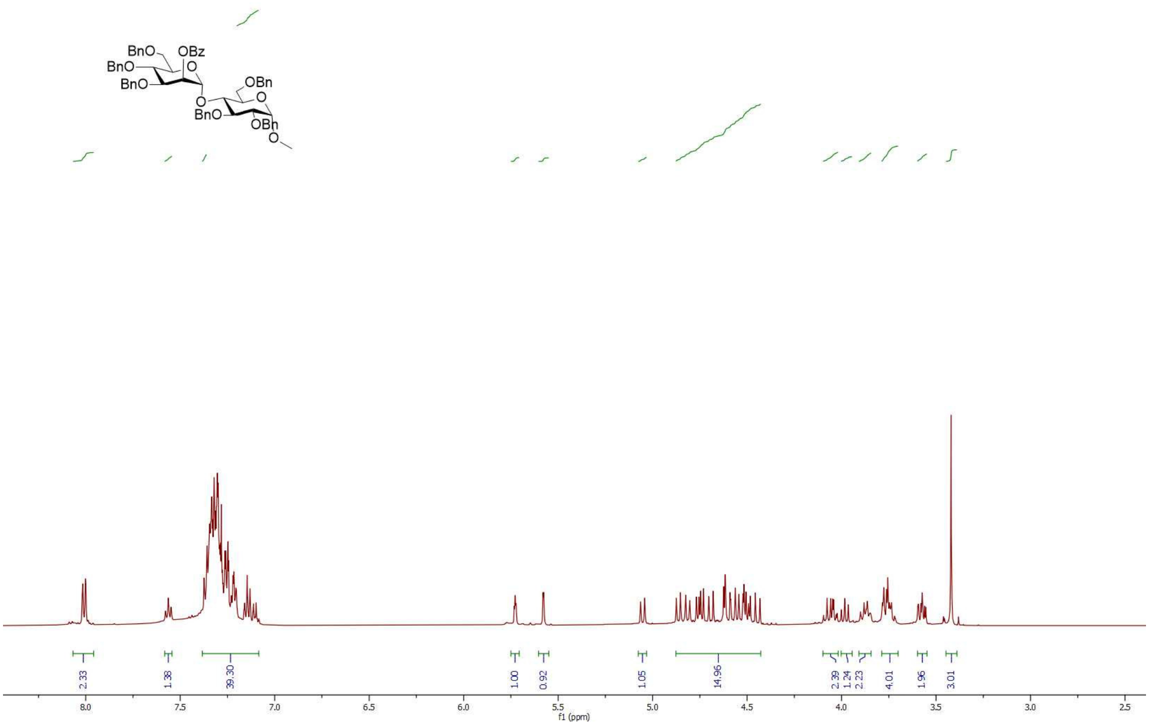

^{13}C NMR (126 MHz, $CDCl_3$)

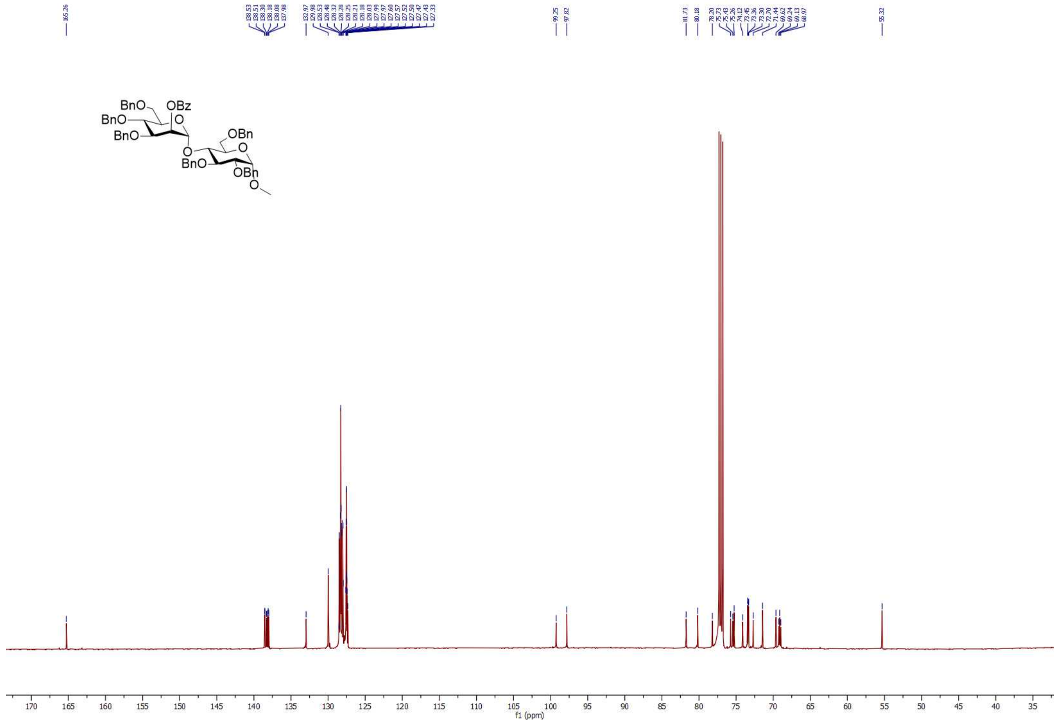

H-H COSY NMR

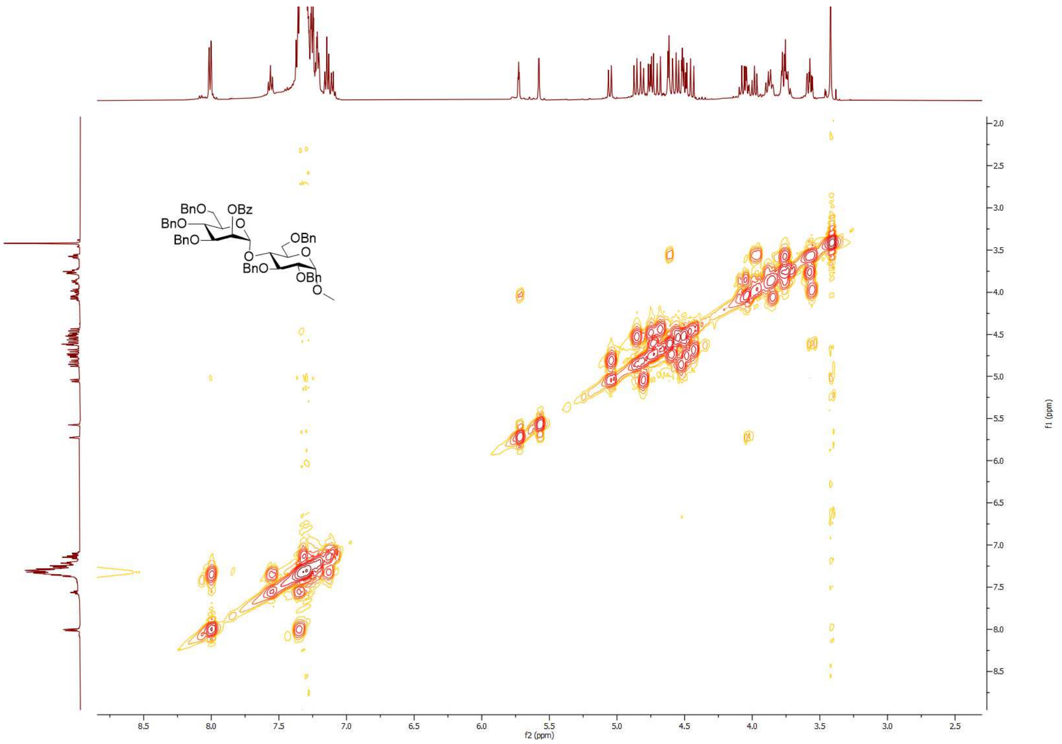

HSQC NMR

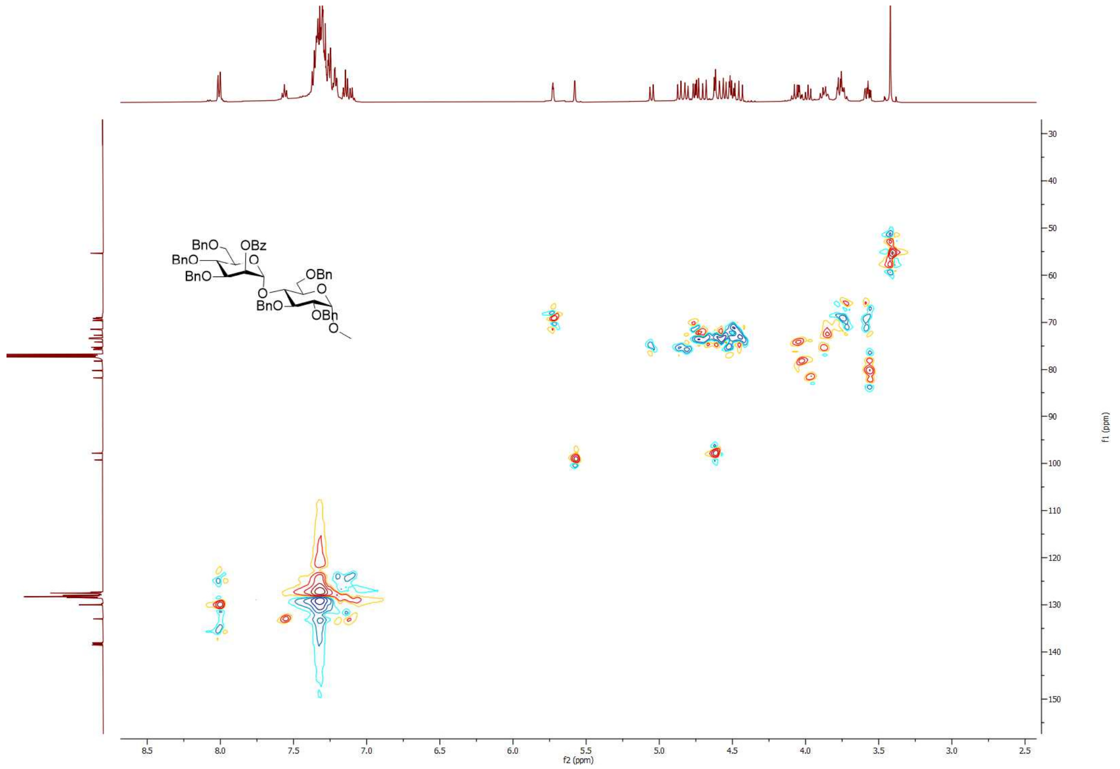

^{1}H NMR (500 MHz, $CDCl_3$)

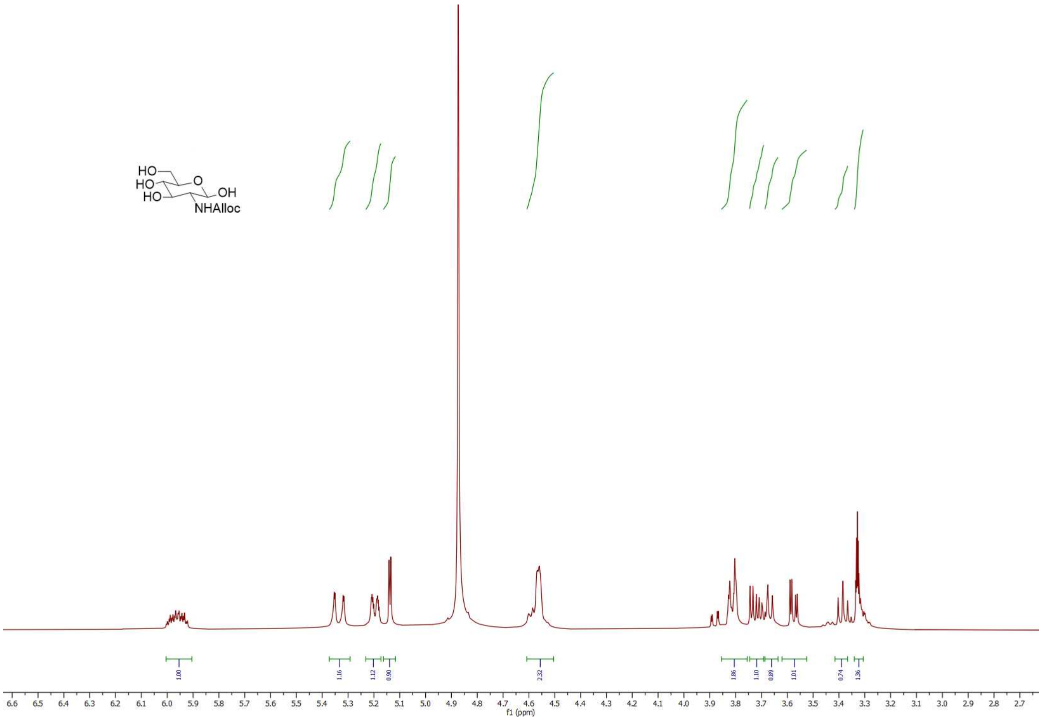

^{13}C NMR (126 MHz, $CDCl_3$)

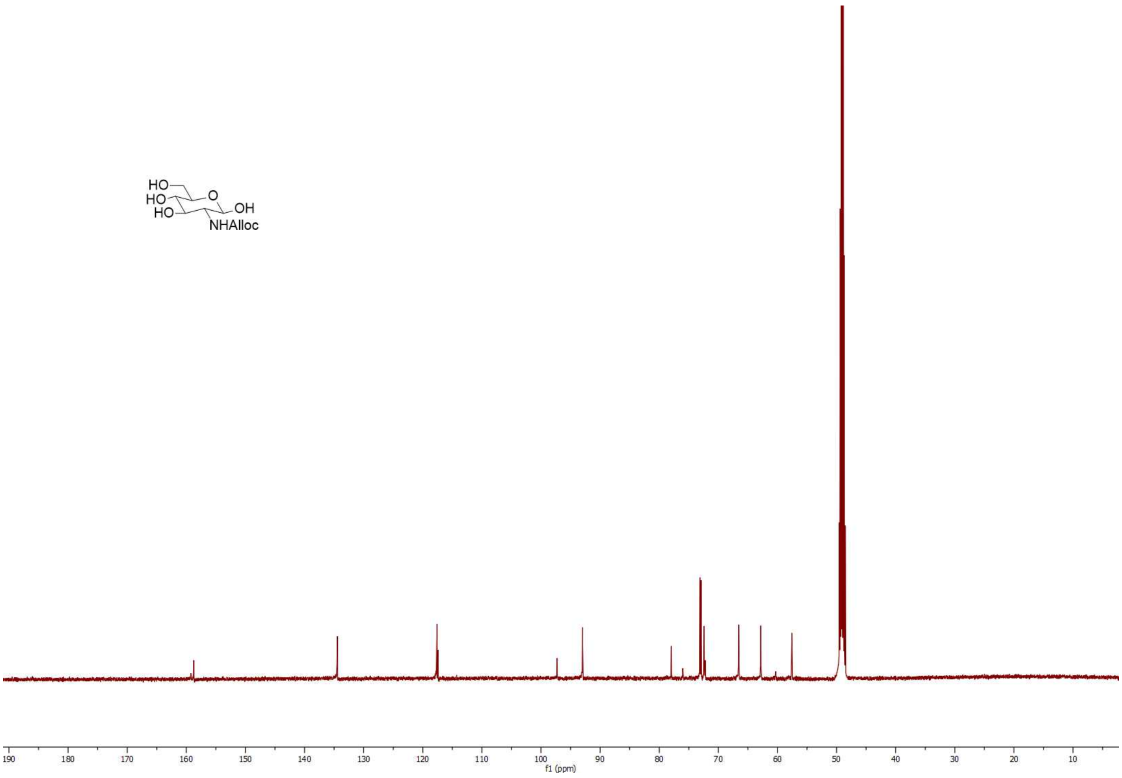

H-H COSY NMR

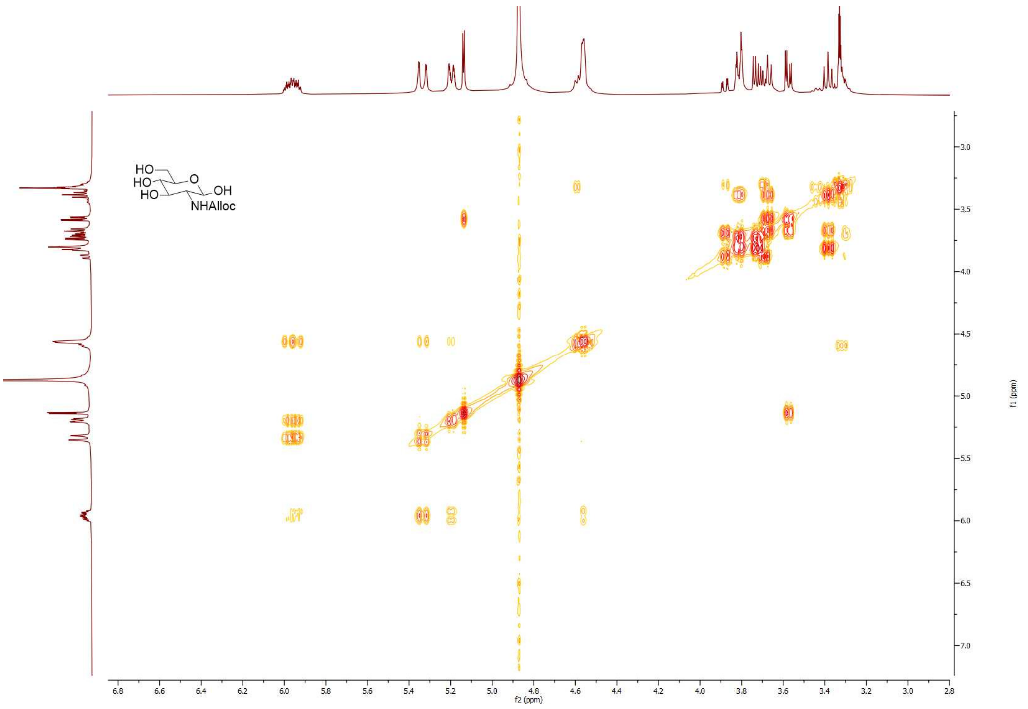

HSQC NMR

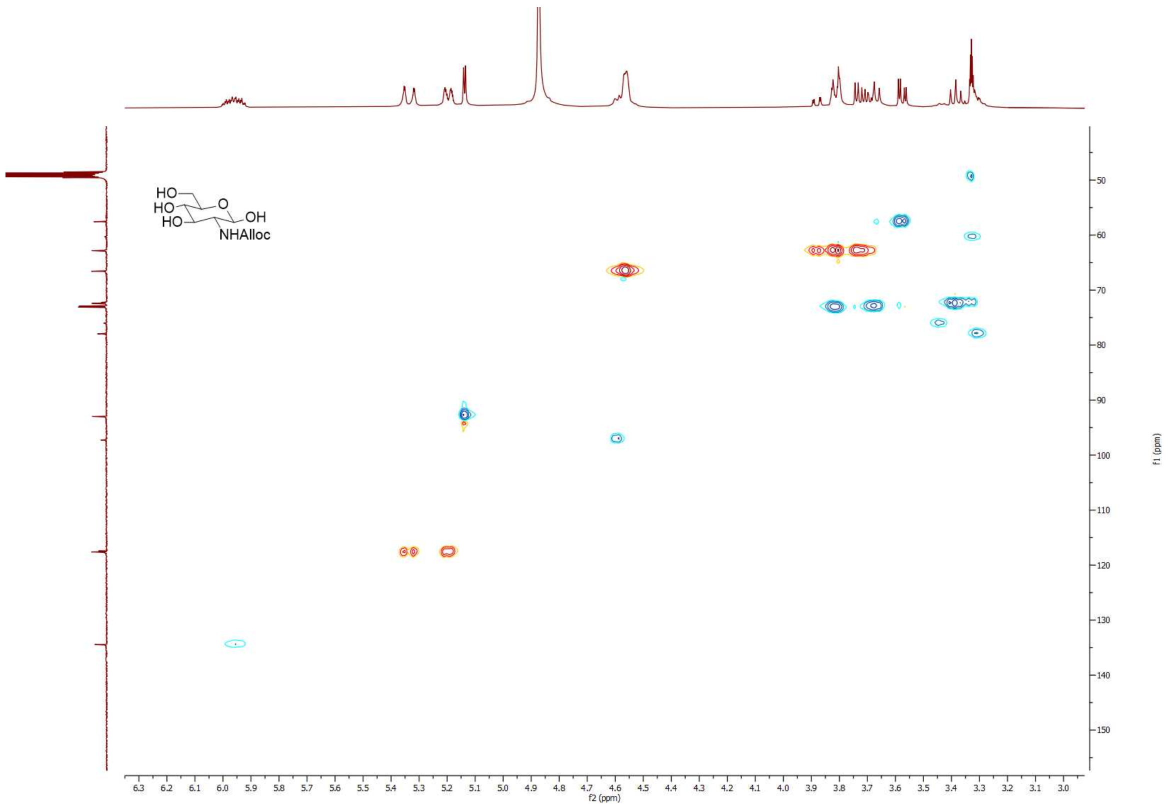

^{1}H NMR (500 MHz, $CDCl_3$)

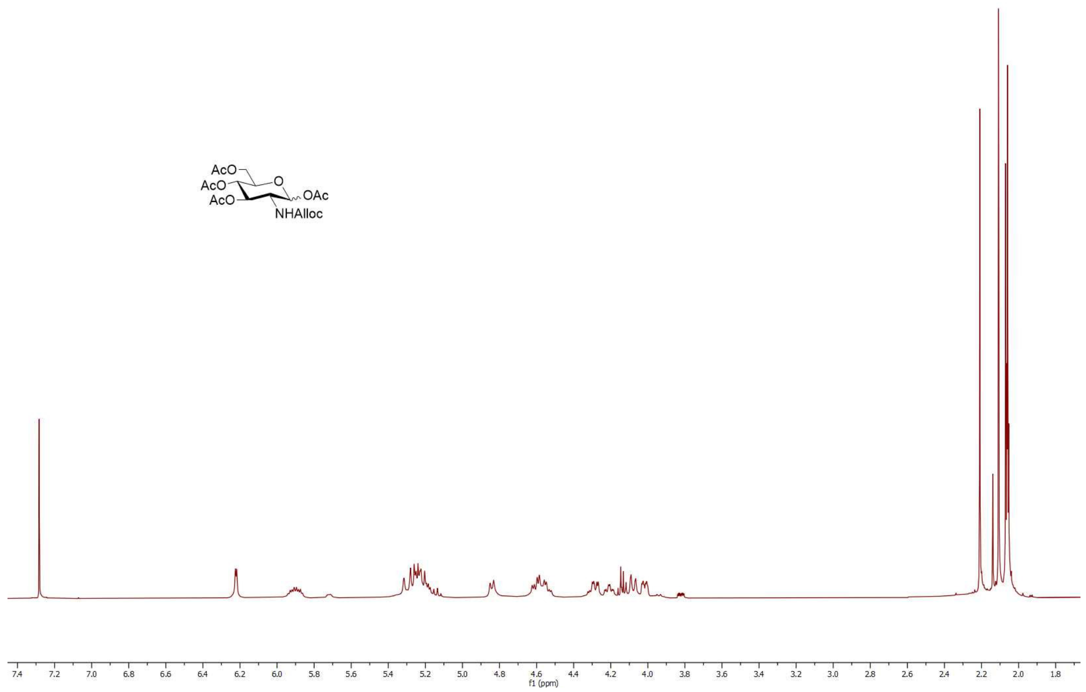

^{13}C NMR (126 MHz, $CDCl_3$)

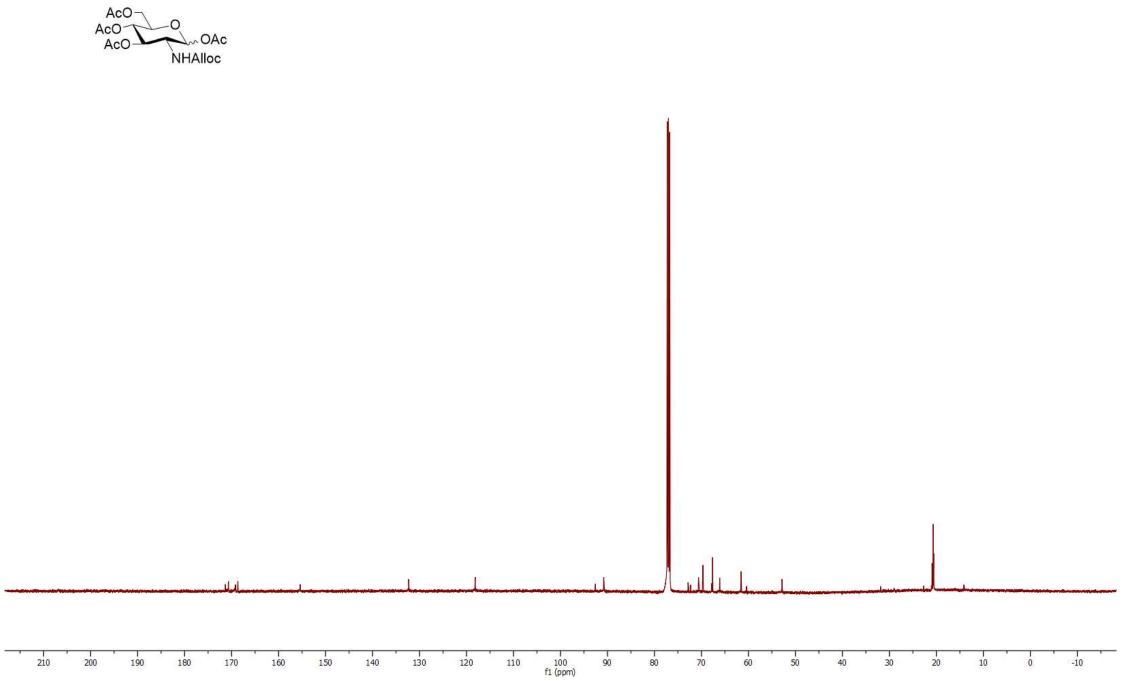

H-H COSY NMR

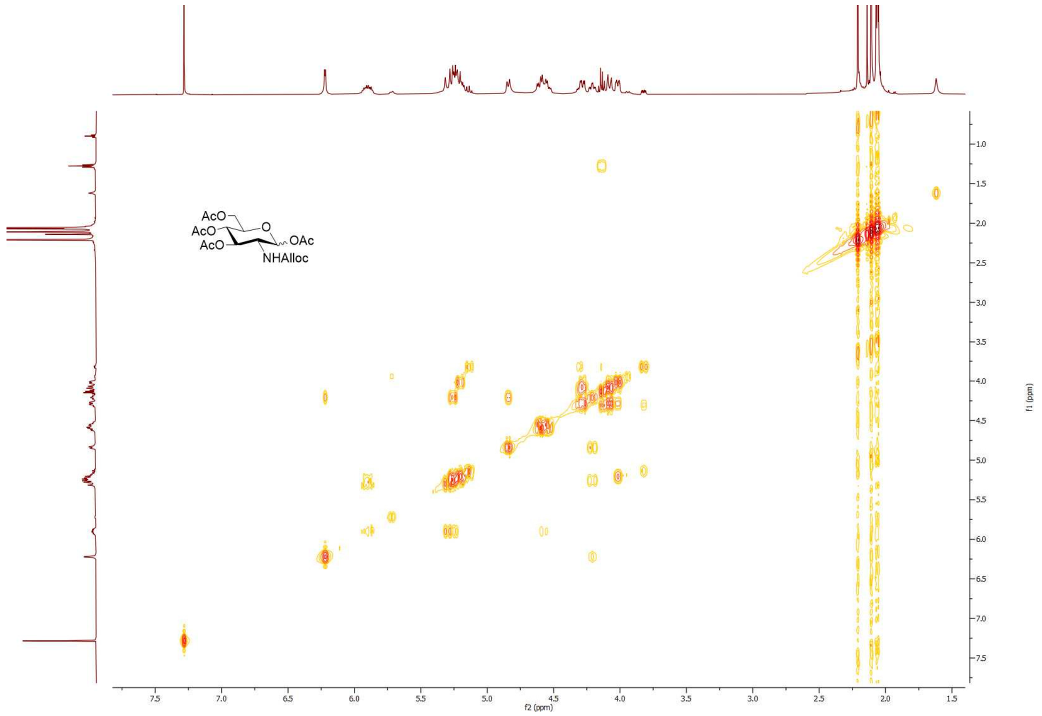
AcO
AcO
AcO
O
OAc
NHAlloc
f2 (ppm)
f1 (ppm)

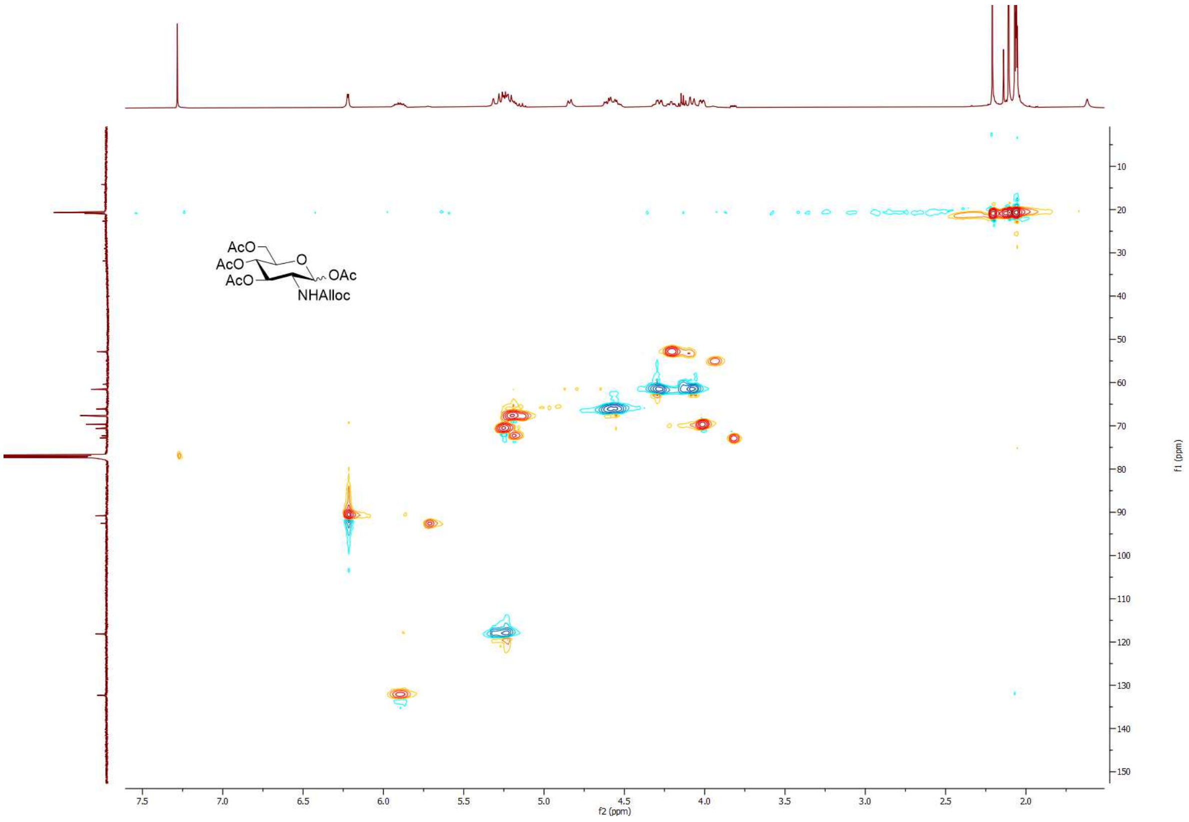
AcO
AcO
AcO
O
OAc
NHAlloc
f2 (ppm)
f1 (ppm)

^{1}H NMR (500 MHz, $CDCl_3$)

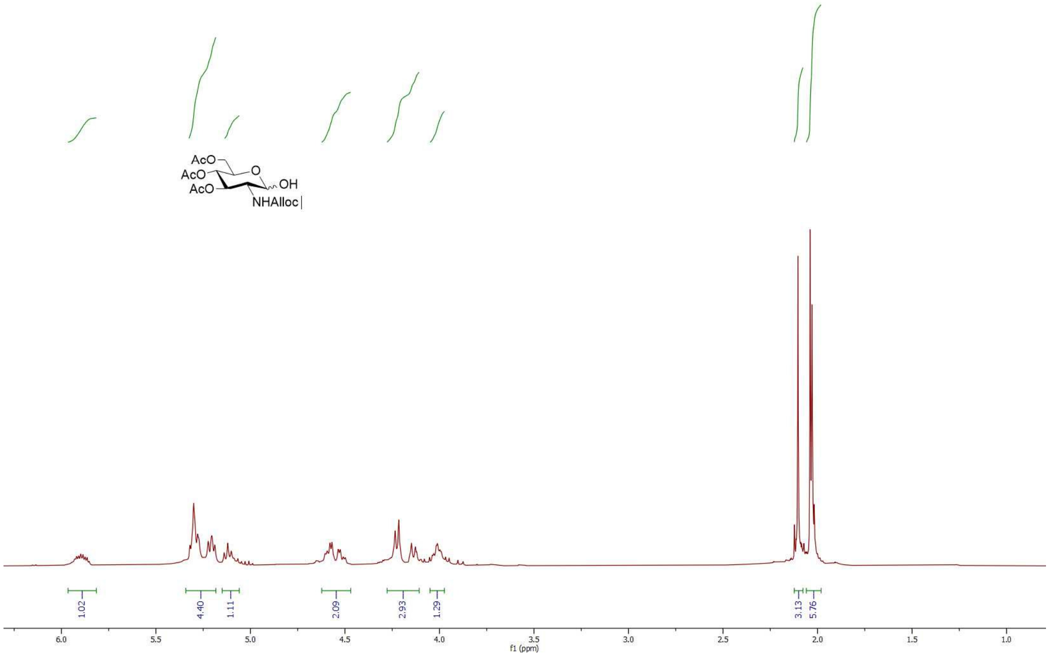

^{13}C NMR (126 MHz, $CDCl_3$)

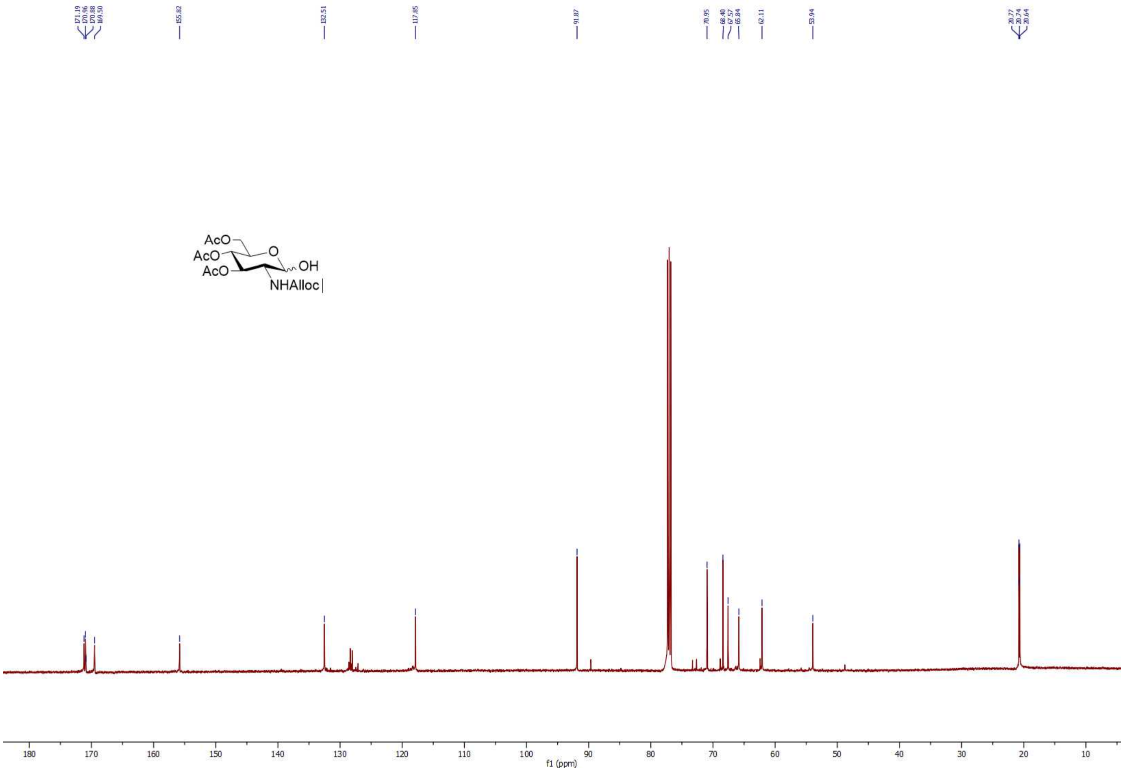

H-H COSY NMR

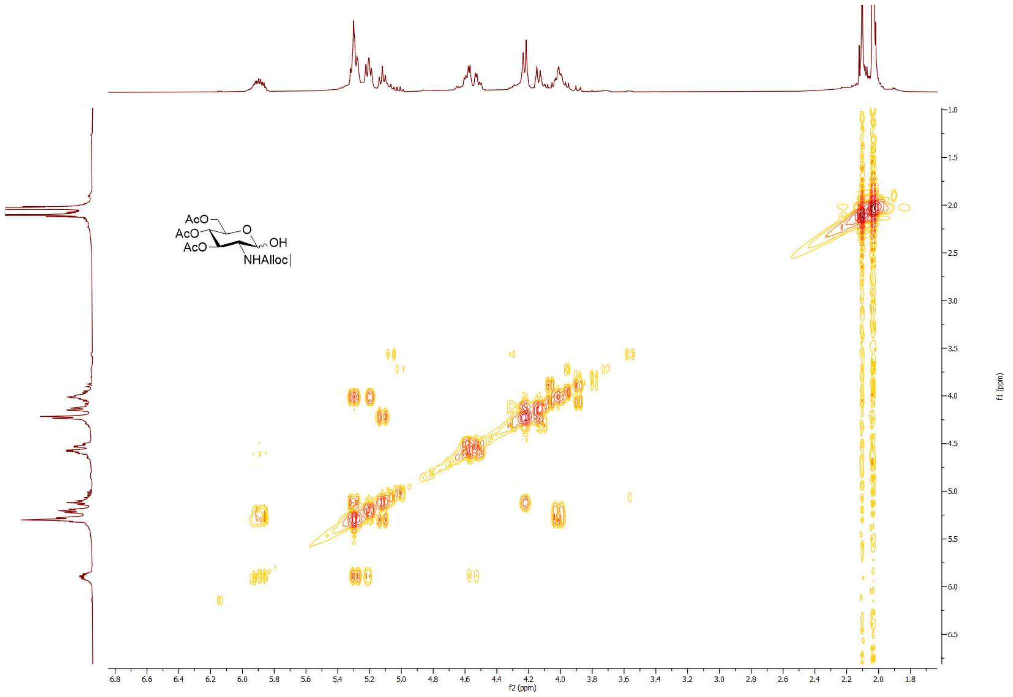

HSQC NMR

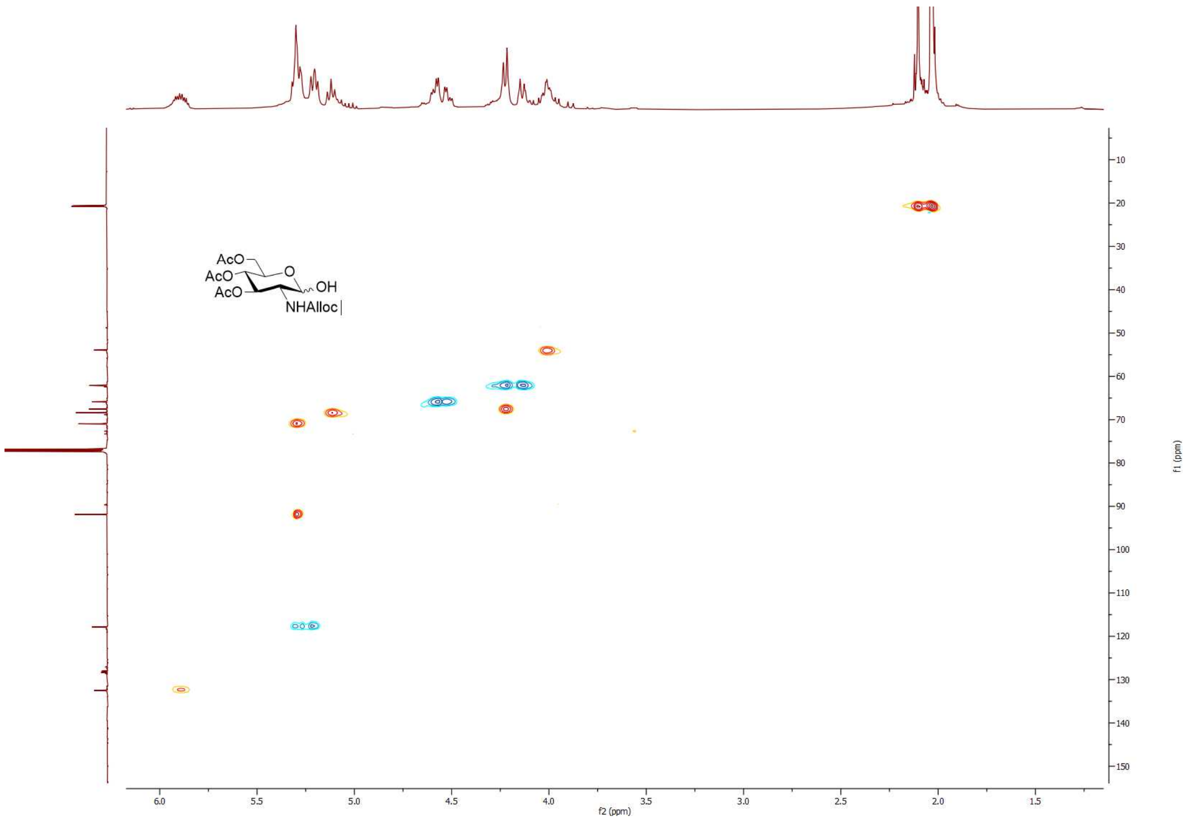

^{1}H NMR (500 MHz, $CDCl_3$)

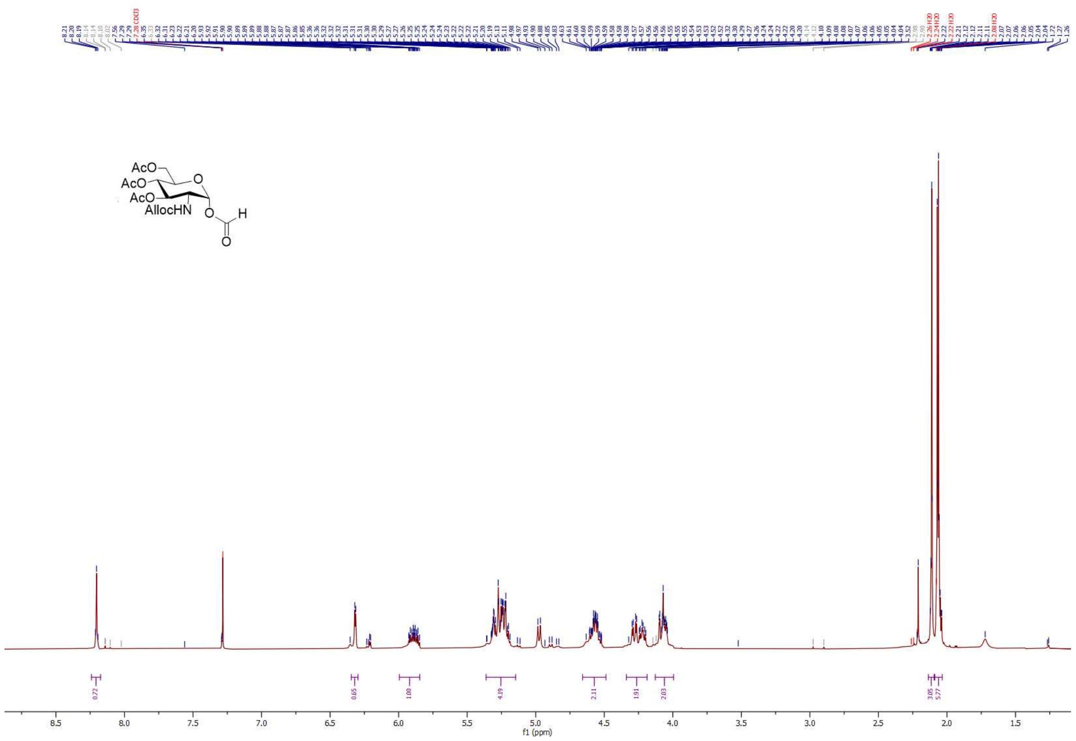

^{13}C NMR (126 MHz, $CDCl_3$)

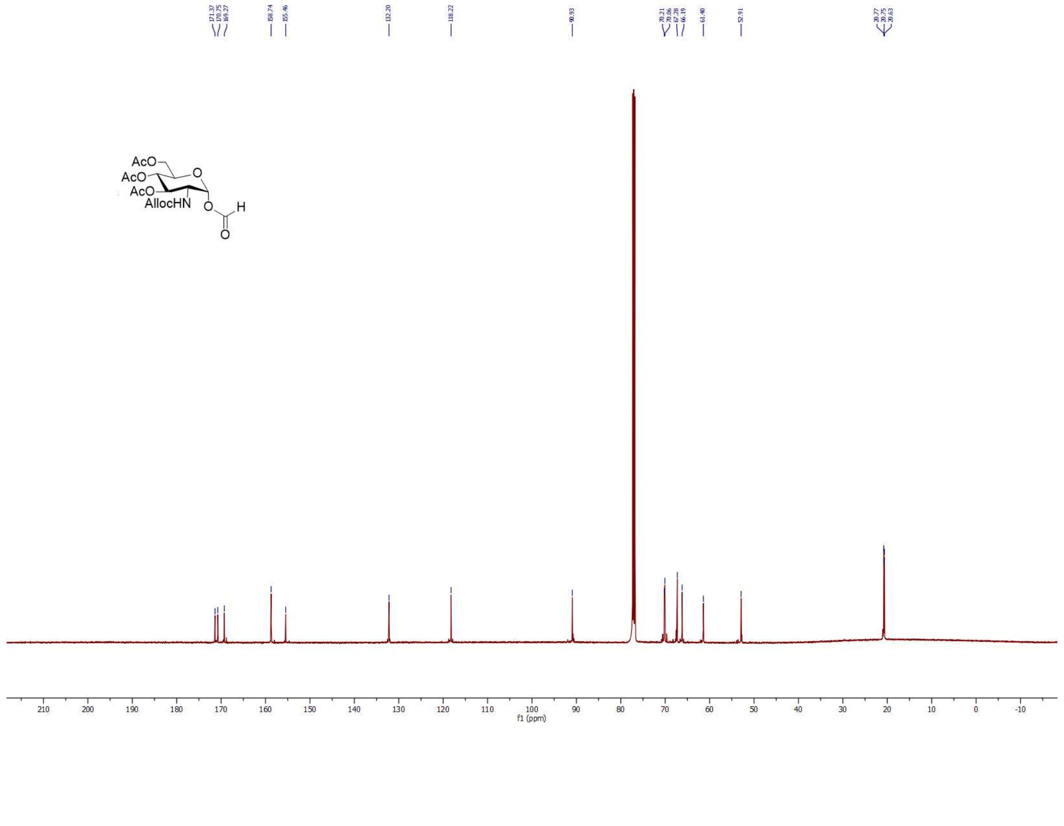

H-H COSY NMR

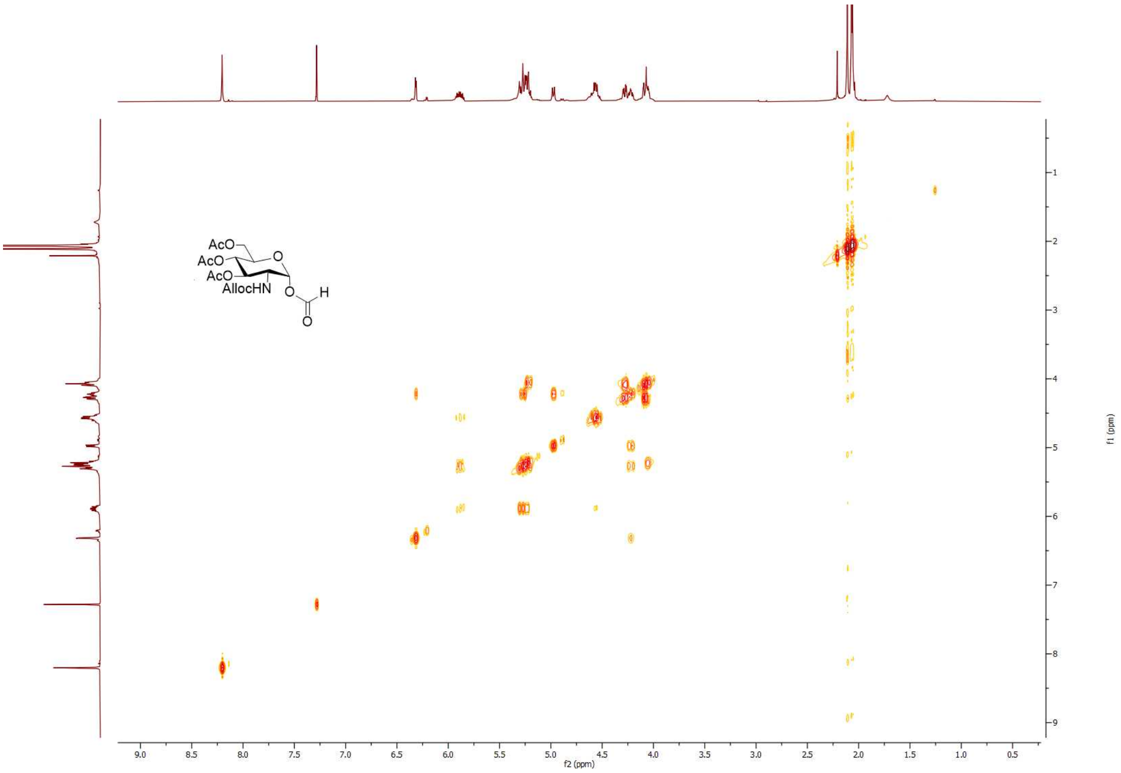

HSQC NMR

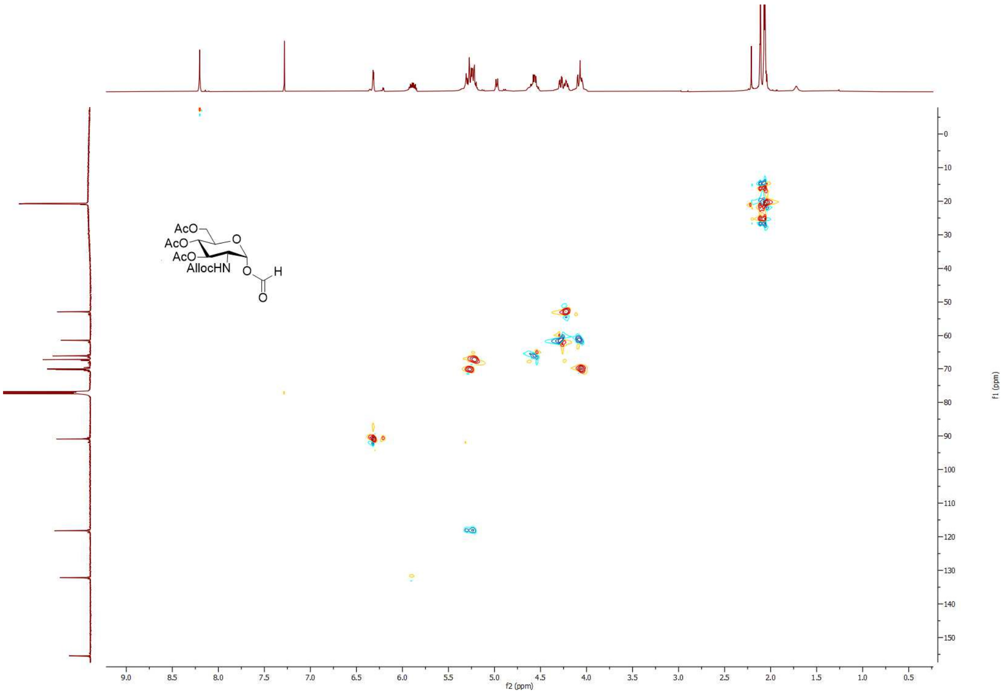

Supplement: Supplementary file 1 [file molecules-27-06244-s001.zip › molecules-1922060-supplementary.pdf]
